# Detection of horizontal gene transfer in the genome of the choanoflagellate *Salpingoeca rosetta*

**DOI:** 10.1038/s41598-021-85259-6

**Published:** 2021-03-16

**Authors:** Danielle M. Matriano, Rosanna A. Alegado, Cecilia Conaco

**Affiliations:** 1 Marine Science Institute, University of the Philippines, Diliman, Quezon City, Philippines; 2 Department of Oceanography, Hawaiʻi Sea Grant, Daniel K. Inouye Center for Microbial Oceanography: Research and Education, University of Hawaiʻi at Manoa, Honolulu, USA

**Keywords:** Evolution, Evolutionary genetics

## Abstract

Horizontal gene transfer (HGT), the movement of heritable materials between distantly related organisms, is crucial in eukaryotic evolution. However, the scale of HGT in choanoflagellates, the closest unicellular relatives of metazoans, and its possible roles in the evolution of animal multicellularity remains unexplored. We identified at least 175 candidate HGTs in the genome of the colonial choanoflagellate *Salpingoeca rosetta* using sequence-based tests. The majority of these were orthologous to genes in bacterial and microalgal lineages, yet displayed genomic features consistent with the rest of the *S. rosetta* genome—evidence of ancient acquisition events. Putative functions include enzymes involved in amino acid and carbohydrate metabolism, cell signaling, and the synthesis of extracellular matrix components. Functions of candidate HGTs may have contributed to the ability of choanoflagellates to assimilate novel metabolites, thereby supporting adaptation, survival in diverse ecological niches, and response to external cues that are possibly critical in the evolution of multicellularity in choanoflagellates.

## Introduction

In Bacteria and Archaea, several mechanisms, such as transformation, conjugation, and transduction^1–8^, facilitate the introduction of novel genes between neighboring strains and species, a process known as horizontal (or lateral) gene transfer (HGT)^9,10^. Horizontal transfers into eukaryote genomes, however, are thought to be low frequency events, as genetic material must enter the recipient cell’s nucleus to be incorporated into the genome and vertically transmitted^11^. While HGT has been difficult to demonstrate in eukaryotic lineages^12^, conflicting branching patterns between individual gene histories and species phylogenies^4^ has garnered a number of explanations: a gene transfer ratchet to fix prey-derived genes (“you are what you eat”)^13^, movement of DNA from organelles to the nucleus through endosymbiosis (e.g. endosymbiotic gene transfer)^14,15^, the presence of “weak-links” or unprotected windows during unicellular or early developmental stages that may enable integration of foreign DNA into eukaryotic genomes^11^, and horizontal transposon transfers^16–18^. Indeed, HGT may have accelerated genome evolution and innovation in microbial eukaryotes by contributing to species divergence^19–21^, metabolic diversity and versatility^22,23^, and the establishment of interkingdom genetic exchange^10,19,24,25^.

Recently, Choanoflagellatea, a clade of aquatic, free-living, heterotrophic nanoflagellates^26,27^, has emerged as a model for understanding the unicellular origins of animals. Choanoflagellates are globally distributed in marine, brackish, and freshwater environments^28^. All choanoflagellates have a life history stage characterized by an ovoid unicell capped by a single posterior flagellum that functions to propel the cell in the water column and to generate currents that sweep food items toward an actin microvilli collar^26,27,29^. Captured food items, such as detritus and a diverse array of microorganisms, are phagocytosed by the organism. The choanoflagellate cellular architecture bears striking similarity to sponge choanocytes (or “collar cells”), leading to speculation on the evolutionary relationship between choanoflagellates and animals^27^. Abundant molecular phylogenetic evidence support choanoflagellates as the closest extant unicellular relatives of metazoans^30–35^.

Choanoflagellates were historically grouped into two distinct orders based on taxonomy^28,32,33,36^: Acanthoecida (families Stephanoecidae and Acanthoecidae), which produce a siliceous cage-like basket exoskeleton or lorica; and non-loricate Craspedida (families Codonosigidae and Salpingoecidae), which produce an organic extracellular sheath or theca^32,33^. Previous studies suggest that silicate transport genes and transposable elements in loricate choanoflagellates may have been acquired from microalgal prey^16,37^. At least 1000 genes, including entire enzymatic pathways in extant choanoflagellates, may have resulted from HGT events^38–46^.

The colonial craspedid *Salpingoeca rosetta* has been established as a model organism for investigating the origins of animal multicellularity^27,47^. In this study, we used sequence-based methods previously employed to identify HGT events in other eukaryotes^48,49^ to detect putative horizontally acquired genes in the *S. rosetta* genome. *S. rosetta* has a rich life history entwined with affiliated environmental bacteria^26,27,50–52^. Transient differentiation from the solitary morphotype into chain or rosette colonies, as well as sexual mating in choanoflagellates, are triggered by distinct prey bacteria^50^. We hypothesized that sources of novel horizontal transfers in *S. rosetta* are primarily from food sources, as has been demonstrated in other phagocytic and parasitic organisms^13,53^. We compared the unique gene signatures (i.e. GC content, codon usage bias, intron number) of potential HGTs against the rest of the *S. rosetta* genome to determine whether the genes were ancient or recent transfers. We also assessed the extent of taxonomic representation of the candidate HGTs in other choanoflagellates. Characterizing the magnitude and functions of HGTs in choanoflagellates may provide insight into their role in the evolution and adaptation of the lineage to new ecological niches and lifestyles. These HGT events may also shed light on the evolutionary history of genes involved in the origin of multicellularity in choanoflagellates and animals.

## Results

### Identification and genic architecture of candidate HGTs in *S. rosetta*

**Figure 1:**
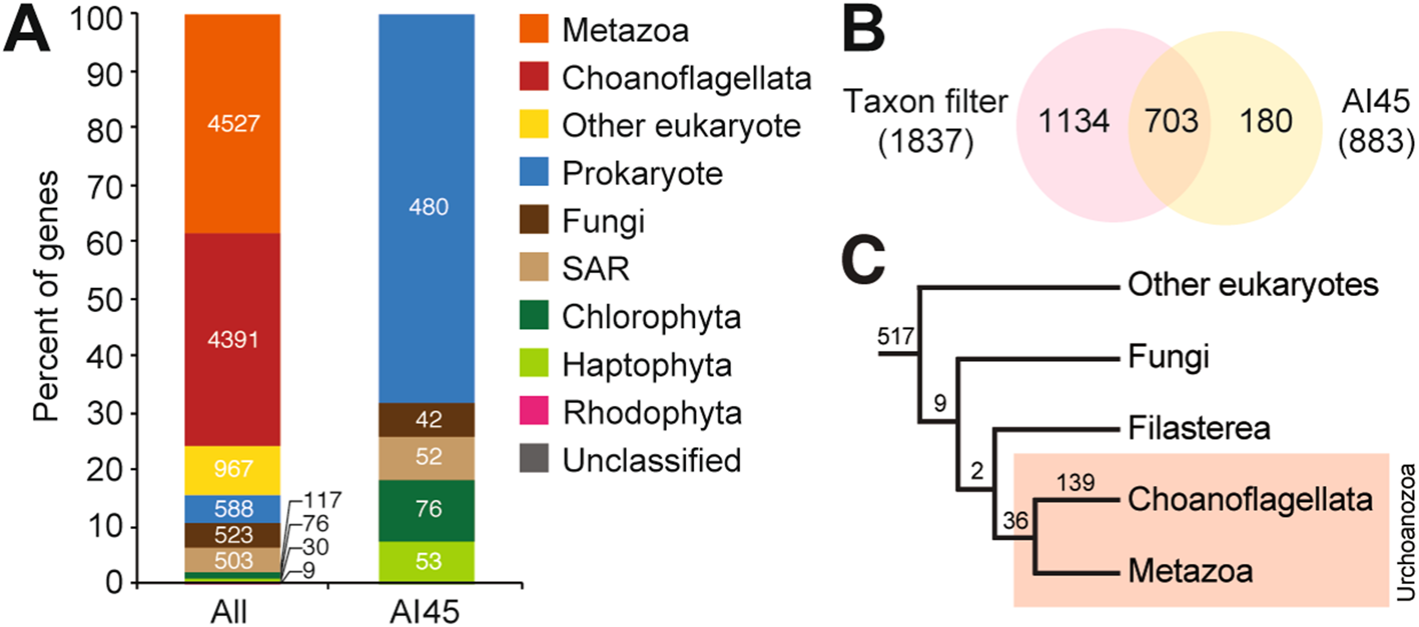
HGT candidates in the *S. rosetta* genome. (**A**) Taxonomic affiliation of all *S. rosetta* genes and genes that passed the Alien Index analysis (AI45). (**B**) Number of putative HGTs detected based on taxonomic affiliation (taxon filter) and Alien Index (AI45) analysis. (**C**) Number of HGT candidates with orthologs in other eukaryotic lineages.

Of the 11,731 reference *S. rosetta* protein coding genes, 4527 (38.59%) were related to metazoan sequences, while 4391 (37.43%) were specific to *S. rosetta* (Fig. 1A). Our taxon filter flagged 2804 (23.90%) genes with highest orthology to sequences from prokaryotes and unicellular non-metazoan taxa. Of these, 1837 (15.66%) genes had best hits to sequences from prokaryotes, fungi, and unicellular eukaryotic taxa with marine representatives, such as chlorophytes, rhodophytes, haptophytes, and the stramenopile, alveolate, and Rhizaria supergroup (SAR) (Fig. 1B). Best hits from prokaryotes (i.e. bacteria and archaea) and unicellular eukaryotic taxa (i.e. unicellular algae and fungi) were scored as potential HGT candidates and subjected to further analysis.

### Identification of candidate HGTs by Alien Index analysis

Using metrics described by Gladyshev et al.^54^, Alien Index (AI) analysis of the 1,837 potential HGT candidate genes identified 703 candidate HGTs from potential bacteria, archaea, and common unicellular marine fungi (i.e. Basidiomycota and Ascomycota) and eukaryotic (i.e. Chlorophyta, Haptophyta, Pelagophyta, and Bacillariophyta) donors (Fig. 1A, Table S1), which included only 38% of the genes flagged by sequence alignment to the NCBI nr database (Fig. 1B). A total of 111 (16%) of the potential HGTs in *S. rosetta* have orthologs to HGT candidates that were previously identified in the choanoflagellate, *Monosiga brevicollis*^40^.

### Orthologs of candidate HGTs in other taxa

To estimate when horizontally transferred genes in the *S. rosetta* genome were acquired, we assessed the number of candidate HGTs with orthologs in 20 other choanoflagellates and in representative eukaryotes, opisthokonts, filasterean, and metazoans, based on OrthoMCL groups or ortholog families identified by the study of Richter et al.^55^ (Table S2). Majority of candidate HGTs flagged in the AI analysis (517 genes) had orthologs in other eukaryotes (Excavata, Diaphoretickes, and Amoebozoa), fungi (9 genes), and filasterean (2 genes). The detection of orthologs of candidate HGTs in multiple eukaryotic lineages likely reflect genes that are present in eukaryotic donor taxa, genes that were transferred into older lineages, or ancestral genes that were lost in multiple lineages but retained in choanoflagellates. Thirty-six genes had orthologs in animals while 139 were only found in choanoflagellates (Fig. 1C). These 175 genes were potentially acquired in the last common ancestor of choanoflagellates and animals (Urchoanozoan) or in the choanoflagellate lineage. Given the difficulty of ascertaining the evolutionary origins of genes with orthologs spanning multiple eukaryotic lineages, succeeding analyses focused only on genes gained in the Urchoanozoan and in the choanoflagellate lineage.

### Genic architecture and expression of candidate HGTs

**Figure 2:**
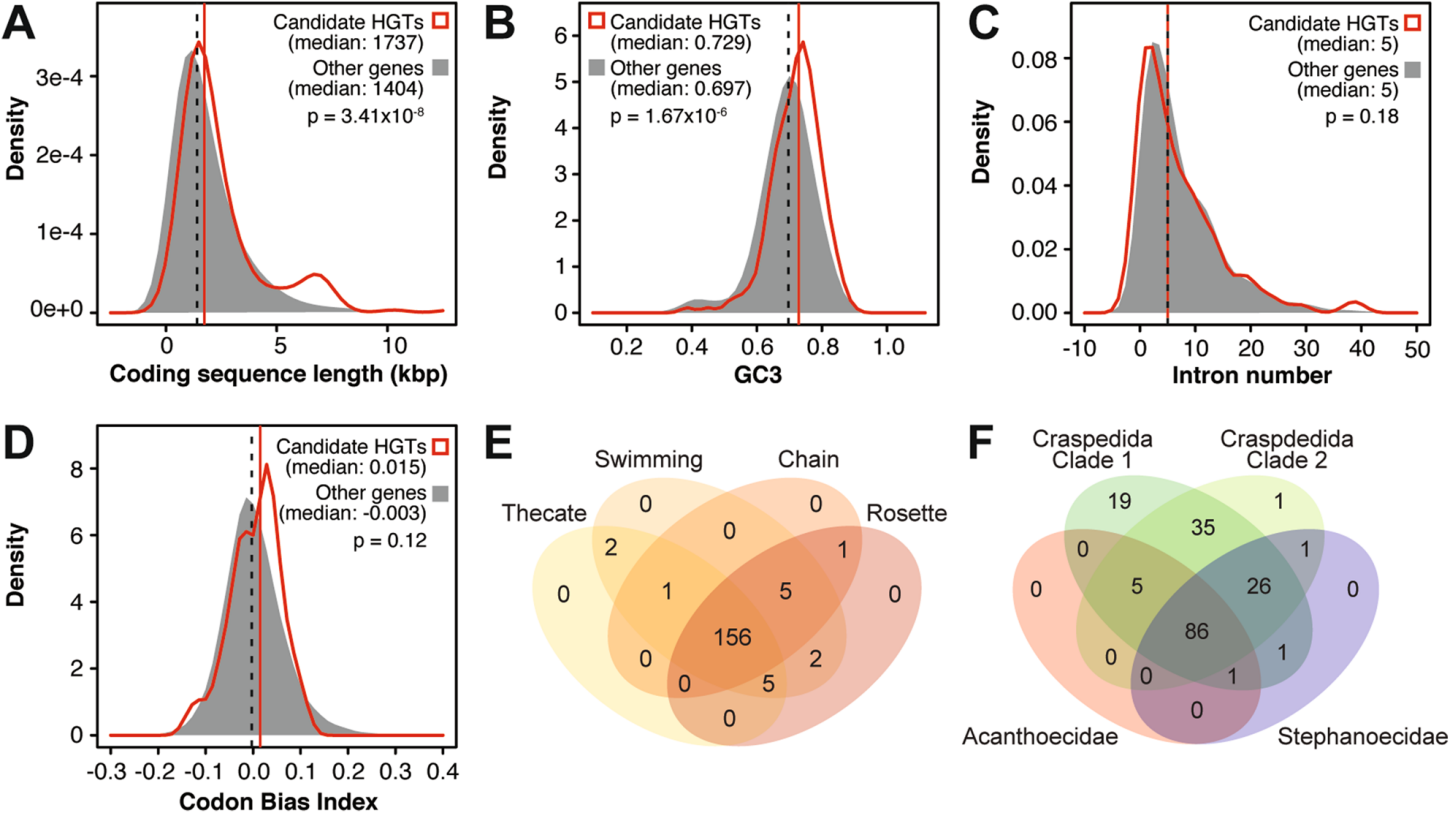
Gene architecture of candidate HGTs. Density plots showing the distribution of (**A**) coding sequence length, (**B**) GC content at the third codon position (GC3), (**C**) intron number, and (**D**) codon bias index in candidate horizontally transferred genes (red) in comparison to the bulk of *S. rosetta* genes (gray). (**E**) Number of HGT candidates that are expressed in the indicated life stages of *S. rosetta*. (**F**) Number of HGT candidates conserved in other choanoflagellate groups.

In *S. rosetta*, the difference in median protein coding sequence length of candidate HGTs (1737 bp) from the overall median CDS length (1404 bp) was small but significant (Kruskal–Wallis test,* p* < 0.001; Fig. 2A). Candidate HGTs also had significantly higher GC content at the third codon position (GC3) relative to other *S. rosetta* genes (Fig. 2B; Kruskal–Wallis test, *p* < 0.001). However, candidate HGTs were observed to have a similar median number of introns, specifically five, as compared to other *S. rosetta* genes (Fig. 2C). Only 1103 (8.79%) of *S. rosetta* genes lacked introns, of which only 23 passed both the AI and OrthoMCL filters. Codon bias index (CBI), which is a measure of codon usage frequency, was also similar between the putative HGTs and other genes (Fig. 2D). In addition, 156 of the candidate HGTs were expressed in the four life stages of *S. rosetta* (i.e. thecate cells, swimming cells, chain colonies, and rosette colonies), 16 genes were expressed in at least one stage, and only 3 were not detected in any of the stages (Fig. 2E, Table S3), based on data from the study of Fairclough et al.^34^. Candidate HGTs were conserved in other choanoflagellates, with 86 genes (49%) found in all choanoflagellate families, 66 genes (38%) found in both Craspedida clades, and 19 genes (11%) found only in Craspedida (clade 1), which includes *S. rosetta* (Fig. 2F).

### Potential sources of *S. rosetta* HGTs

**Figure 3:**
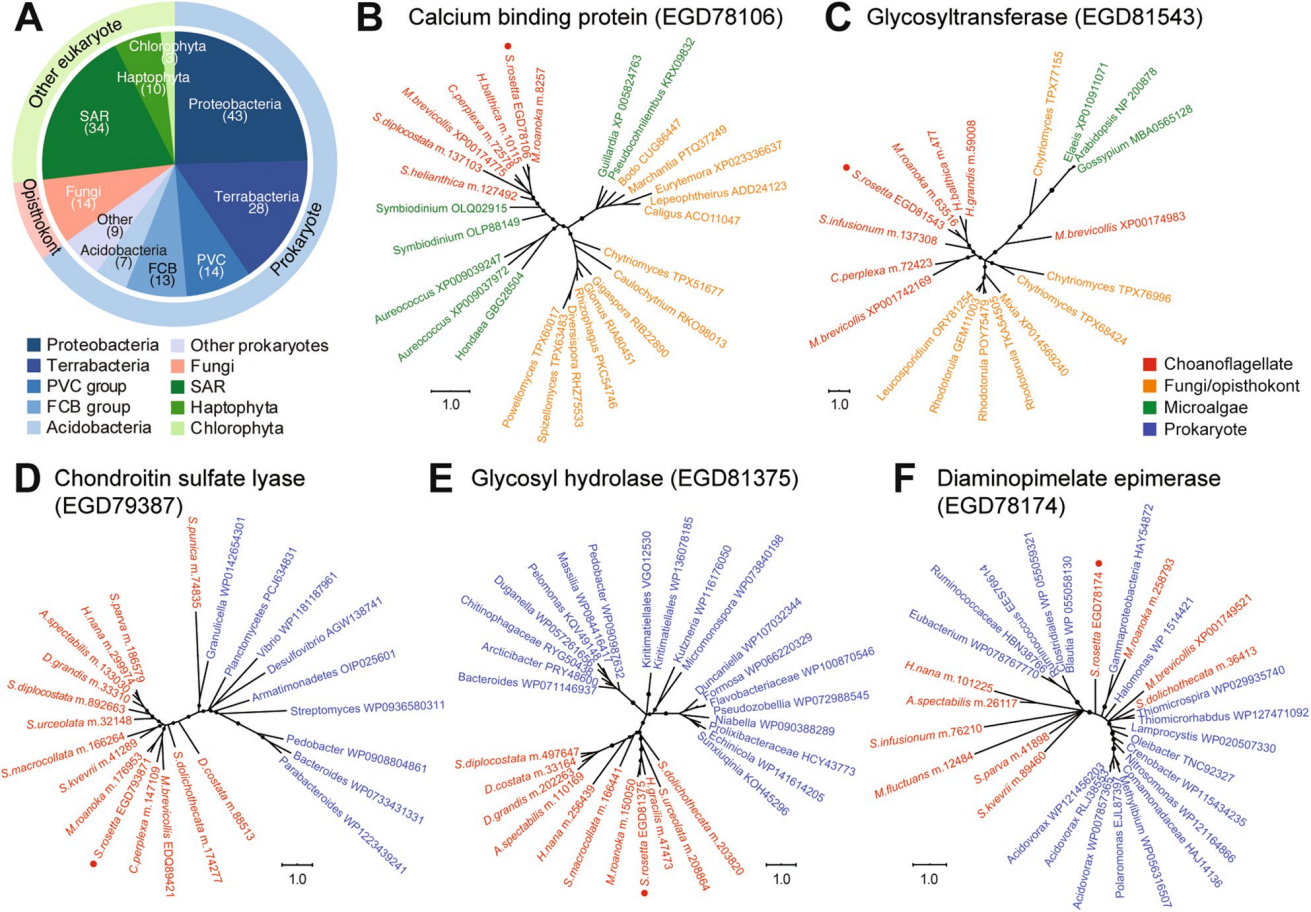
Potential donor phyla of candidate HGTs in *S. rosetta*. (**A**) Taxon affiliation of candidate HGTs based on the best sequence match for each gene. Phylogenetic analysis of selected candidate HGTs, including (**B**) calcium binding protein of microalgal origin, (**C**) glycosyltransferase of fungal origin, and (**D**) chondroitin sulfate lyase, (**E**) glycosyl hydrolase, and (**F**) diaminopimelate epimerase of prokaryotic origin. Genes from choanoflagellates are shown in red, fungi or opisthokonts in orange, microalgae in green, and prokaryotes in blue. *S. rosetta* genes are indicated by a red dot. Trees were generated using MrBayes 3.2.6. Circles at the branches indicate posterior probabilities of 0.70–1.00.

Majority of the candidate HGTs exhibited highest similarity to bacteria (65%), unicellular algae (27%), and unicellular fungal (Ascomycota and Basidiomycota) (8%) sequences (Fig. 3A). Phylogenetic analysis of 130 of 175 putative HGTs revealed conflicting phylogenetic signals relative to the consensus eukaryote reference phylogeny of Richter et al.^55^ (Fig. 3B–F, Fig. S1), confirming their possible origin from donor taxa. The most common potential bacterial donors belonged to the following phyla: Proteobacteria, Terrabacteria, Planctomycetes, Verrucomicrobia, and Chlamydiae (PVC) group, and Fibrobacteres, Chlorobi, and Bacteroidetes (FCB) group (Fig. 3A). At least 4 sequences may have been derived from marine microorganisms that *S. rosetta* potentially interacts with, including known food sources, such as *Vibrio* spp. (Proteobacteria) (3 genes) and *Cytophaga* spp. (Bacteroidetes) (1 gene)^50^*.*

### Functions of candidate HGTs in *S. rosetta*

**Figure 4:**
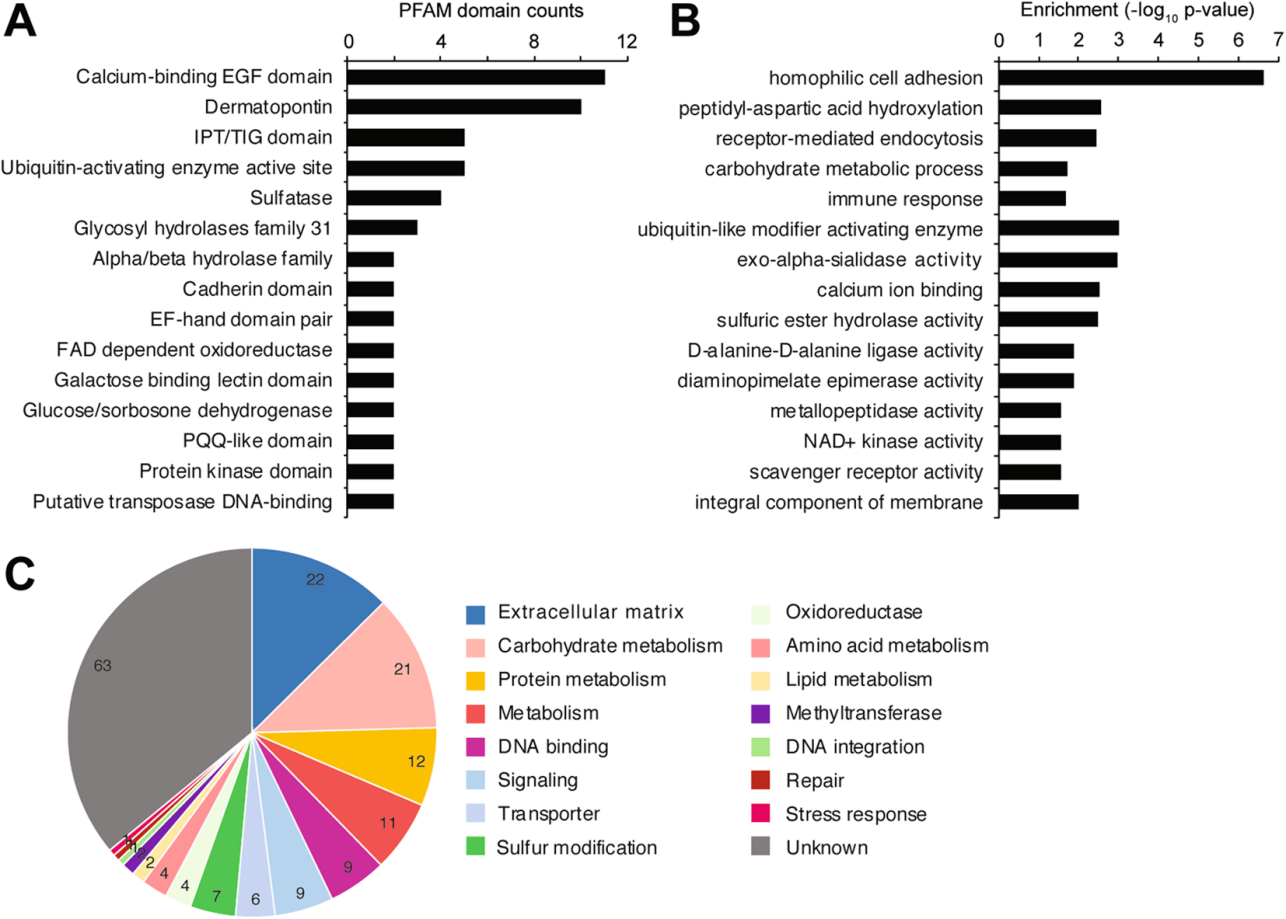
Functional analysis of candidate HGTs. (**A**) Most common PFAM protein domains in the set of candidate HGTs. (**B**) Gene ontology functions enriched in the set of putative HGTs. Enrichment p-values (*p* ≤ 0.05) for selected functions are shown. (**C**) Number of candidate HGTs with associated functions based on manual curation.

Of the 175 candidate HGTs in *S. rosetta*, 125 (71%) had identifiable PFAM domains and 114 (65%) were assigned gene ontology (GO) annotations (Table S1). 41 (23%) candidate HGTs contained two or more annotated PFAM domains. The most notable protein domains represented in the set of putative HGTs included enzymes (i.e. sulfatase, dehydrogenase, ubiquitin-activating enzyme active site, pyrroloquinolone quinolone-like domain, hydrolases, oxidoreductases, transposases), ECM-associated domains (i.e. dermatopontin, calcium-binding EGF domain, IPT/TIG, cadherin, galactose-binding lectin), and signaling domains (i.e. protein kinase) (Fig. 4A, Table S4).

Gene ontology analysis further supported the finding that majority of HGT candidates were enzymes with a variety of catalytic activities and were associated with cellular membranes where most biosynthetic and energy transduction processes of the cell occur^56^ (Fig. 4B, Table S5). More specifically, potential HGTs in *S. rosetta* were associated with functions related to carbohydrate and protein metabolic processes (i.e. carbohydrate metabolism, peptidyl-aspartic acid hydroxylation, diaminopimelate epimerase activity), and adhesion and signaling (i.e. homophilic cell adhesion, receptor-mediated endocytosis, calcium ion binding, scavenger receptor activity).

### Selected candidate HGTs

To further examine the potential functions of candidate HGTs, the genes were manually curated and assigned to a general cellular function based on GO affiliations and PFAM domains (Fig. 4C, Table S6). The most common functions within the set of putative HGTs were extracellular matrix (ECM) components. ECM-associated genes identified as potential HGTs included 10 dermatopontin/calcium-binding EGF (DPT/Ca^2+^ EGF) domain-containing genes. These genes had orthologs in craspedids but not in loricate choanoflagellates. Genes containing DPT/Ca^2+^ EGF domains had no orthologs in representative filastereans but were present in higher animals, including cnidarians and bilaterians. Two chondroitin sulfate lyase genes were also detected as potential HGTs in the AI analysis, although only one gene (EGD79387) passed the OrthoMCL filter and was unique to the choanoflagellate lineage.

Functions related to the metabolism of carbohydrates and proteins were also common within the set of candidate HGTs. Carbohydrate metabolism-related HGTs included 6 genes with glycosyl hydrolase domains and 2 with glycosyltransferase domains. Protein metabolism-related genes included various peptidases and ubiquitin-activating enzymes. Several genes involved in amino acid metabolism were also identified as HGTs, including diaminopimelate epimerase (*dapF*), a gene involved in lysine biosynthesis through the diaminopimelic acid (DAP) pathway. It should be noted that other DAP-associated genes, specifically, aspartate-semialdehyde dehydrogenase (*asd*) and diaminopimelate decarboxylase and aspartate kinase (*lysAC*), as well as other enzymes involved in the biosynthesis of histidine, threonine, methionine, cysteine, tryptophan, and arginine^57^, were also flagged as candidate HGTs by AI analysis but did not pass the OrthoMCL filter (Fig. S2). Candidate HGTs related to the metabolism of amino acids have orthologs in other choanoflagellates, sponges, and cnidarians.

## Discussion

Our analysis provides evidence of a rich repertoire of *S. rosetta* genes that may have been acquired through horizontal gene transfer. Recent gene acquisitions are usually distinguished by divergent genetic characteristics, such as GC content, codon usage bias, and genetic architecture (e.g. intron content, coding sequence length)^58,59^. Over time, transferred genes undergo sequence changes to adapt to host genome characteristics, enabling improved transcription and translation^60^. A majority of *S. rosetta* HGT candidates had gene features similar to the rest of the genome and were expressed, based on the study by Fairclough et al.^34^, indicating that these acquisitions were not recent^34^. We noted a higher GC content at the third codon position of the HGT candidate genes, which is a typical marker for genes derived from bacterial sources^61,62^, yet the genes did not exhibit a divergent intron count. It is possible that foreign genes with translationally optimal codons and high GC3 content that resembles the GC-rich genome of *S. rosetta* are more likely to be positively selected, as this allows for more efficient translation and greater expression. Alternatively, selection for genes with metabolic functions, which tend to have a higher GC bias^63^, may also explain the high GC3 content of the horizontally acquired genes*.* The prevalence of introns in *S. rosetta* candidate HGTs further indicate adaptation of acquired genes to the intron-rich genome of *S. rosetta*. Moreover, most HGT candidates had orthologs in other choanoflagellate taxa, suggesting that the gene transfer events occurred before divergence of the various choanoflagellate groups. Nevertheless, phylogenetic analysis of the candidate HGTs revealed incongruent gene trees, with candidate HGTs clustering with genes from potential donors, including bacteria, unicellular algae, and fungi. This suggests that candidate horizontally transferred genes in *S. rosetta* were acquired from multiple prokaryotic and unicellular eukaryotic donors.

Most of the potential donors of HGTs in *S. rosetta* clustered with possible bacterial donors, in contrast to *M. brevicollis* where HGTs were identified as coming mostly from algal donors^40^. *S. rosetta* is an active phagotroph of bacteria, such as *A. machipongonensis* and *Vibrio* spp., which have also been shown to influence its development and metabolic processes^26,27,50,52^. Thus, the major mechanism of HGT in *S. rosetta* may be through the engulfing of food or associated microbes. HGT events may also be facilitated by the activity of TEs, which are abundant in the genome of *S. rosetta*^16^. However, it should be noted that because some taxonomic lineages are underrepresented in sequence databases and since some HGTs may have been integrated into the host genome for a long time, the identification of the donor species for HGTs is not straightforward.

Most candidate HGTs with known functions were orthologous to operational genes, such as enzymes that function in amino acid and carbohydrate biosynthesis, as well as genes that function in intercellular signaling and in the establishment and modification of ECM components. Based on the complexity hypothesis proposed by Jain et al.^22^, gene transferability is dependent on two factors: gene function and protein–protein interaction/network interaction^22,23,64–70^. Operational genes are more likely to be passed horizontally because they can function independently of other genes^40,48,54,71–74^. On the other hand, informational genes or genes involved in transcription and translational processes, physically interact with more gene products, limiting their functionality when transferred individually and reducing the possibility that they will be successfully retained as HGTs^65^.

The acquisition of genes through ingestion of prey corroborates the “you are what you eat” gene transfer ratchet theory, which suggests that the evolution of the nuclear genome of most protists was driven by acquisition of exogenous genes by phagotrophy or engulfment and acquisition of gene fragments from their food sources^13^. This mode of acquisition allows for the transfer of more diverse genetic functions, as opposed to a more selective one via endosymbiosis. Interestingly, horizontal gene transfer in diverse eukaryotic lineages often involves enzymes that function in common metabolic pathways^40,41,46,48,53,54,72,75^. As mentioned above, the selective retention of these types of genes may be due to their ability to function independently and to be incorporated into pre-existing metabolic processes^22,76^. The acquisition of novel functions through HGT can extend the metabolic capability of the host, allowing it to explore and establish new niches or adapt to various environmental conditions. Horizontally acquired genes may also mediate interactions with other organisms in the environment and facilitate life stage transitions. The contribution of novel functions acquired via HGT may be greater in choanoflagellates like *M. brevicollis* and *S. rosetta* that have retained fewer ancestral gene families compared to other choanoflagellates^55^.

Novel combinations of protein domains could potentially enhance catalytic efficiency and functional novelty of enzymes. The genome of *S. rosetta* contains a rich complement of multidomain genes that may have contributed to its unique biology and morphology^31,34^. It is possible that some of these molecular innovations emerged through gene fusion or domain shuffling events that incorporated pre-existing domains with domains acquired from horizontally transferred genes, thereby expanding adaptive functional novelty of genes in choanoflagellates and other eukaryotes^77^.

Many of the putative HGTs in *S. rosetta* contribute to nutrient acquisition and metabolic processes by enabling efficient use of available organic substrates. Conservation of horizontally acquired proteases and glycosyl hydrolases in phagotrophic choanoflagellates suggests their importance in digesting various food sources and adapting to an environment high in plant biomass, such as the mud core samples from where *S. rosetta* was isolated^27,40,78^. Proteases and glycosyl hydrolases have also been flagged as candidate HGTs in *M. brevicollis,* bdelloid rotifers, sponge, rumen ciliates, and fungi^40,54,72,78,79^.

HGTs with functions related to amino acid biosynthesis contribute to the metabolic flexibility of *S. rosetta*. Some of these enzymes, particularly those involved in the DAP pathway of lysine biosynthesis, as well as those involved in the biosynthesis of arginine, threonine and methionine, have previously been identified as potential HGTs in *M. brevicollis*^40,41^*.* Conservation of these HGTs in the choanoflagellate lineage and their absence in some metazoans, filastereans, and fungi, suggest that they were likely transferred into older lineages and retained in choanoflagellates. Retention of genes involved in amino acid metabolism in choanoflagellates may contribute to unique metabolic competencies. On the other hand, these genes have been lost in the animal stem lineage^80,81^ as multicellular animals rely on direct acquisition of essential amino acids from their diet^82^. Cnidarians, however, regained the ability to synthesize aromatic amino acids tryptophan, phenylalanine, and other aromatic compounds through HGT events^80^.

*Salpingoeca rosetta* has several life stages regulated by extrinsic factors. Its genome harbors multiple adhesion receptors and cell membrane enzymes for substrate attachment, cell–cell communication, and colony formation that may regulate these life stage transitions^26,34,50–52,55^. Among these are candidate HGTs, including several genes with dermatopontin (DPT/Ca^2+^ EGF) domains, which may function similar to dermatopontin, an acidic multifunctional matrix protein that promotes cell adhesion, ECM collagen fibrillogenesis, and cell assembly mediated by cell surface integrin binding^83–87^. It is also a major component of the organic matrix of biomineralized tissues (e.g. mussels)^88^. Absence of orthologs of DPT/Ca^2+^ EGF domain-containing genes in some loricate choanoflagellates suggests the possible importance of these genes in the production of the organic theca in Craspedida.

*Salpingoeca rosetta* also has multiple glycosyl hydrolases and glycosyltransferases that were potentially acquired through horizontal transfer. Some glycosyl hydrolases and glycosyltransferases were found exclusively in the choanoflagellate lineage. These enzymes are known to sculpt the ECM of animals by changing the structure of the extracellular matrix components and by modifying functional groups on molecules to regulate their interactions^89,90^. Glycosyl hydrolases may degrade proteoglycans to control the mating process of *S. rosetta*^52,91^. Glycosyltransferases, on the other hand, regulate key signaling and adhesion proteins like cadherins and integrins^92–94^.

The *S. rosetta* genome contains two chondroitin sulfate lyase genes, both of which are candidate HGTs from potential bacterial donors, although only one gene is unique to the choanoflagellate lineage. It was suggested that these proteins may be involved in endogenous processes for regulating mating in *S. rosetta*^52^. It is also possible that chondroitin sulfate lyases contribute to the ability of choanoflagellates to modify cell walls and extracellular matrix proteins, which may facilitate transitions from one life stage to the next. Chondroitin sulfate lyase cleaves chondroitin sulfate glycosaminoglycans (GAGs) via an elimination mechanism resulting in disaccharides or oligosaccharides^95^. GAGs are typically found as side chains on proteoglycans on cell membranes and the ECM of animal tissues where they regulate processes such as adhesion, differentiation, migration, proliferation, and cell–cell communication^95^. The proteoglycan, chondroitin sulfate, was found to partially suppress the adhesive properties of dermatopontin^83–87^, suggesting that breakdown of chondroitin sulfate through lyase activity may promote stronger cell adhesion. The bacterium, *Vibrio fischerii*, produces a chondroitin sulfate lyase called *EroS* (or “extracellular regulator of sex”) that induces mating in the choanoflagellate, *S. rosetta*^52^. The conservation of chondroitin sulfate lyase genes in *S. rosetta* and most choanoflagellate representatives suggest that these genes may constitute unique adaptive mechanisms in choanoflagellates. Further studies on the expression of these genes in different environmental conditions are needed in order to better understand their potential functions in the host.

As with other studies on the detection of HGTs in eukaryotes, it is important to note that the current work is limited by the lack of broader taxon representation in publicly available databases. In addition, parametric tests and Alien Index analysis may not accurately estimate the number of HGT events, particularly for ancient gene transfers that have been retained in metazoans. Moreover, it can be difficult to distinguish HGT events from gene gain or gene loss events in the last universal common ancestor, as both result in patchy distribution of genes in the species tree. For many of the candidate HGTs in *S. rosetta*, presence of homologs in only a few lineages also constrained phylogenetic analyses to just a few representative taxa. These limitations may have resulted in the underestimation of the number of horizontally acquired genes in *S. rosetta*. Further development of methods to effectively filter out false positives and fine tune the results of HGT analysis is needed to reveal the true extent of horizontally acquired genes in the last common ancestor of choanoflagellates and animals.

In conclusion, we have shown that the genome of *S. rosetta* contains a rich repertoire of genes potentially acquired through horizontal transfer. Most genes were possibly ancient horizontal transfers gained prior to the divergence of choanoflagellates, as evidenced by similar genomic signatures and expression motifs to the host, as well as conservation in other choanoflagellate taxa. Horizontal acquisition of genes may have played a key role in the diversification of cellular metabolic processes and contributed novel functions that enhanced the catalytic ability of enzymes, thereby allowing *S. rosetta* to colonize diverse ecological niches. In addition, the acquisition of genes involved in the extra cellular senses and sensory responses to the environment, through the induction of reproduction and multicellular development, may have helped shape choanoflagellate evolution and multicellular life stages. We anticipate that future expression analysis of these promising candidate HGTs will provide a more in-depth understanding on their potential roles in choanoflagellates and animal multicellularity.

## Materials and methods

### Identification of HGTs by sequence alignment

The 11,731 predicted *S. rosetta* protein sequences downloaded from the Ensembl protist database^96^ (http://www.ensembl.org/; last accessed October, 2018) were aligned to the NCBI non-redundant (nr) protein database (http://www.ncbi.nlm.nih.gov/; last accessed October, 2018), comprised of protein sequences from GenBank, EMBL, DDBJ, PDB, and RefSeq, using Diamond^97^ local sequence alignment with a threshold E-value of 1 × 10^–5^. The taxonomic affiliation of hits were retrieved from NCBI taxonomy (http://www.ncbi.nlm.nih.gov/taxonomy), and a Python^98^ script was used to collect hits with specific taxon IDs. Only sequences with the lowest E-value corresponding to bacteria, archaea, unicellular algae (i.e. Chlorophyta, Stramenopiles, Bacillariophyta, Pelagophyta, and Haptophta), or unicellular fungi (i.e. Ascomycota and Basidiomycota) were considered as potential HGTs; peptides with no hits to the indicated taxa were disregarded from further analysis.

### Determining the Alien Index scores of candidate HGTs

Alien Index (AI) analysis quantitatively measures how well the *S. rosetta* protein sequences align to non-metazoan versus metazoan protein sequences^54^. The E-value of the best sequence alignment match of *S. rosetta* peptides against all metazoan or non-metazoan gene sequences from the NCBI nr database were used to compute the AI score of each gene using the formula^54^:

$$AI = \log \left( {\left( {best \, metazoan \, E \, value} \right) + 1E - 200} \right) - {\text{ log}}\left( {\left( {best \, non{ - }metazoan \, E \, value} \right) + 1E - 200} \right).$$

In cases where *S. rosetta* sequences had no hits to non-metazoans (excluding choanoflagellates) or metazoans, the E-value was set to 1. Genes that scored ≥ 45 were classified as foreign, 0 ≤ AI ≤ 45 as indeterminate, and less than 0 as metazoan genes^54^. Orthologs of the foreign genes or candidate HGTs were determined using OrthoMCL group data from the study of Richter et al.^55^. Only genes gained in the Urchoanozoan stem were included in subsequent analyses.

### Genomic feature analysis of candidate HGTs

Genomic features of putative HGTs, specifically GC content at the third codon position (GC3) and codon usage, as represented by the codon bias index (CBI), were determined using correspondence analysis on CodonW^99^. Coding sequence (CDS) lengths and intron numbers were determined from published information on the *S. rosetta* genome on Ensembl^96^. To determine if there were mean differences between the genomic features of candidate HGTs compared to all *S. rosetta* genes, we performed Kruskal–Wallis test in RStudio version 1.2.1335^100^ with R version 3.6.1^100^. Density plots were generated using the sm package^101^ in R and edited using Adobe Illustrator version 24.2.1.

### Gene expression analysis

To examine expression of candidate HGTs, we obtained transcriptome data from the study of Fairclough et al.^34^. Sequence libraries representing various life stages of *S. rosetta* were downloaded from the NCBI Short Read Archive (rosette cells, SRX042054; chain cells, SRX042047; thecate cells, SRX042052; swimming cells, SRX042053). Reads were mapped against the predicted cDNA sequences of *S. rosetta* using kallisto^102^ with default settings to obtain gene expression values in transcripts per million reads (TPM).

### Protein domain and gene ontology analysis

Identification of protein domains and gene ontology associations was conducted on Blast2GO^103^ to determine possible functions of candidate HGTs in *S. rosetta*. Gene ontology terms associated with each predicted peptide were determined from its best Diamond hit to the UniProt database^104^ at an E-value ≤ 1 × 10^–5^. Enriched functions in the set of putative HGTs versus non-HGTs were identified using the Fisher’s exact test in topGO^105^ in R package version 3.6.1^100^. Only functions with an FDR-corrected p-value ≤ 0.05 were considered statistically significant.

### Phylogenetic analysis of candidate HGTs

Peptide sequences of candidate HGTs were aligned with representative sequences from selected metazoa, eukaryotic, and prokaryotic taxa using MAFFT version 7^106^. Alignment confidence scores were calculated using GUIDANCE2^107^ and alignments were trimmed using Gblocks^108^ with default settings to remove ambiguous and divergent protein alignments. For each protein sequence, Akaike information criterion (AIC) and Bayesian information criterion (BIC) scores were calculated in MEGAX^109^. The best evolutionary model for phylogeny between different models tested for the protein sequences was determined by using the calculated AIC and BIC scores that had the smallest score difference, in this case, mtrev substitution model was consistently used. Markov Chain Monte Carlo (MCMC) parameters of each analysis were set to 100,000 generations sampled every 100 trees. By default, the first 25% of the trees were discarded as burn-in. Bayesian phylogenetic trees were constructed using MrBayes 3.2.6^110^. Trees were edited online using Interactive Tree Of Life (iTOL)^111^ and Adobe Illustrator version 24.2.1. Genes with an insufficient number of homologs, an alignment confidence score ≤ 70%, or ambiguous alignment regions were not included in this analysis.

## Supplementary Information

Supplementary Information 1.

Supplementary Information 2.

## Data Availability

The datasets generated during and/or analyzed in the current study are available in Figshare: https://figshare.com/projects/Detection_of_Horizontal_Gene_Transfer_in_the_Genome_of_the_Choanoflagellate_Salpingoeca_rosetta/79619.
