# Supplementary material for: Detection of horizontal gene transfer in the genome of the choanoflagellate Salpingoeca rosetta
Source: Sci Rep. 2021 Mar 16;11:5993. doi: 10.1038/s41598-021-85259-6 (PMC7971027; doi:10.1038/s41598-021-85259-6)
Supplement: Supplementary file 1 — Supplementary Information 1. [file 41598_2021_85259_MOESM1_ESM.pdf]

Supplemental Information

Detection of Horizontal Gene Transfer in the Genome of the Choanoflagellate *Salpingoeca rosetta*

Danielle M. Matriano[1], Rosanna A. Alegado[2], and Cecilia Conaco[1]

[1]Marine Science Institute, University of the Philippines, Diliman

[2]Department of Oceanography, Hawai‘i Sea Grant, Daniel K. Inouye Center for Microbial Oceanography: Research and Education, University of Hawai`i at Manoa

List of Supplemental Information

Supplemental Tables (provided in a separate Excel file)

Table S1. Summary of HGT analyses for all *S. rosetta* genes

Table S2. Average expression of candidate HGTs in transcripts per million reads (TPM)

Table S3. Orthologs of candidate HGTs in other taxonomic groups. Presence of an ortholog in at least one member of the group is indicated by 1 while no ortholog detected is represented by 0, based on OrthoMCL data from Richter et al. (2018)[1]. Represented taxa include Excavata (Naegleria); Diaphoretickes (Chondrus, Guillardia, Chlamydomonas, Arabidopsis, Emiliania, Bigelowiella, Tetrahymena, Thalassiosira); Amoebozoa (Acanthamoeba, Dictyostelium); Fungi (Coprinopsis, Rhizopus, Schizosaccharomyces, Homolaphlyctis, Yarrowia); Filasterea (Capsaspora); Choanoflagellates (21 species); Sponges (Oscarella, Ephydatia, Amphimedon); Ctenophores (Mnemiopsis); Other animals (Trichoplax, Cnidaria - Hydra, Nematostella, Acropora, Bilateria - Ciona, Gasterosteus, Mus, Branchiostoma, Strongylocentrotus, Caenorhabditis, Pristionchus, Daphnia, Drosophila, Tetranychus, Capitella, Helobdella)

Table S4. PFAM domains in candidate HGTs

Table S5. Gene ontology enrichment in candidate HGTs

Table S6. Associated functions of candidate HGTs

Supplemental Figures

Figure S1. Unrooted phylogenetic trees of 130 candidate HGTs in *S. rosetta* (S1-1 to S1-124). Phylogenetic trees were generated using MrBayes 3.2.6[2]. Text colors indicate taxon affiliation (red, choanoflagellate; blue, prokaryote; green, microalgae/plant; orange, opisthokont/fungi). Posterior probabilities of 0.70-1.00 are indicated as dots on selected branches. Trees were edited online using Interactive Tree of Life (iTOL)[3] and Adobe Illustrator version 24.2.1.

Figure S2. Candidate HGTs in amino acid biosynthetic pathways. Pathway map showing genes identified as HGTs in both *Salpingoeca rosetta* and *Monosiga brevicollis* (blue lines). Candidate HGTs in *S. rosetta* that passed the Alien Index and OrthoMCL filters are indicated by yellow lines, while those that passed only the Alien Index filter are indicated by red lines. Text colors indicate potential donors of candidate HGTs (blue, bacteria; green, microalgae; yellow, fungi). The pathway map image was obtained from the Kyoto Encyclopedia of Genes and Genomes[4] (https://www.kegg.jp/kegg/) and edited using Adobe Illustrator version 24.2.1.

Figure S1. Unrooted phylogenetic trees of 87 candidate HGTs in *S. rosetta* (S1-1 to S1-124). Phylogenetic trees were generated using MrBayes 3.2.6[2]. Text colors indicate taxon affiliation (red, choanoflagellate; blue, prokaryote; green, microalgae/plant; orange, opisthokont/fungi). Posterior probabilities of 0.70-1.00 are indicated as dots on selected branches. Trees were edited online using Interactive Tree of Life (iTOL)[3] and Adobe Illustrator version 24.2.1.

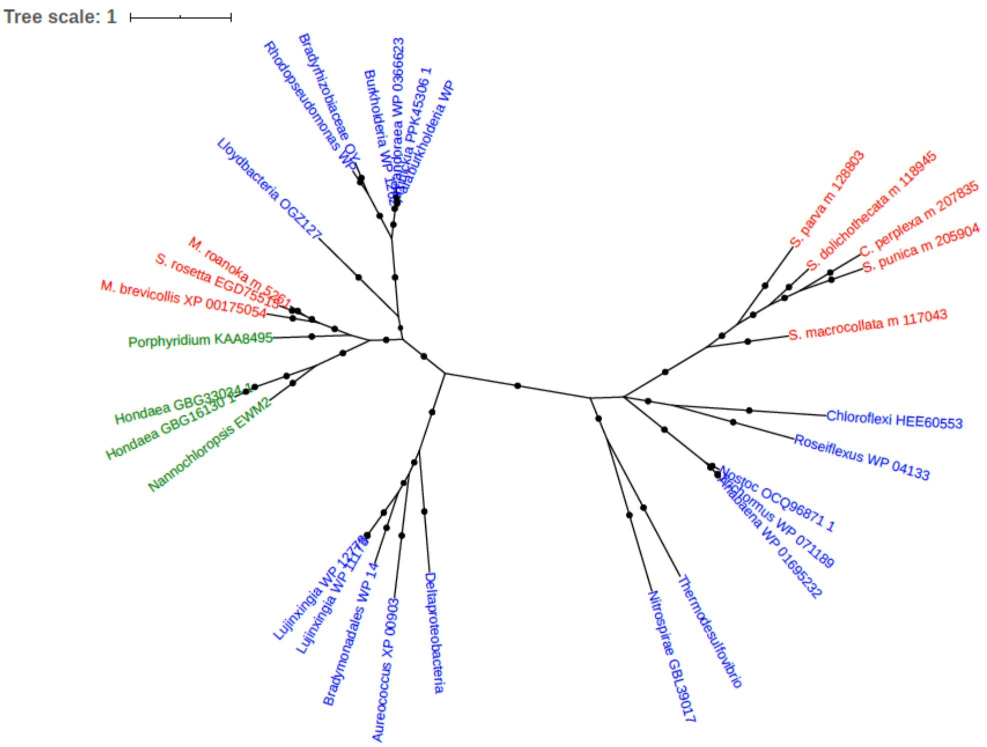

Figure S1-1. 3-dehydroquinate synthase (aroB) (EGD75515)

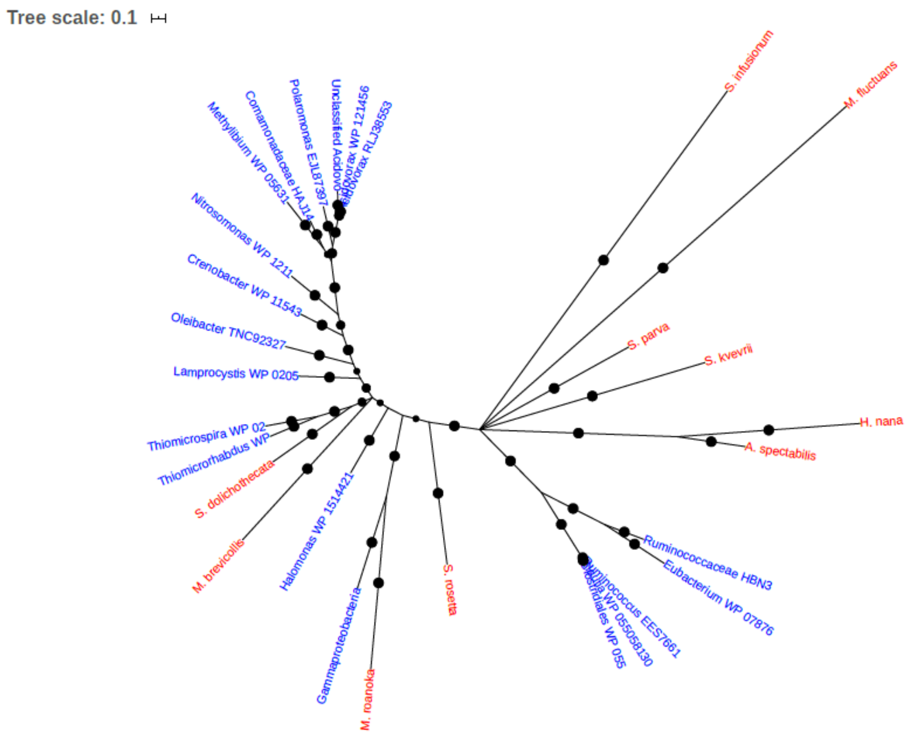

Figure S1-2. B Diaminopimelate epimerase (dapF) (EGD78174)

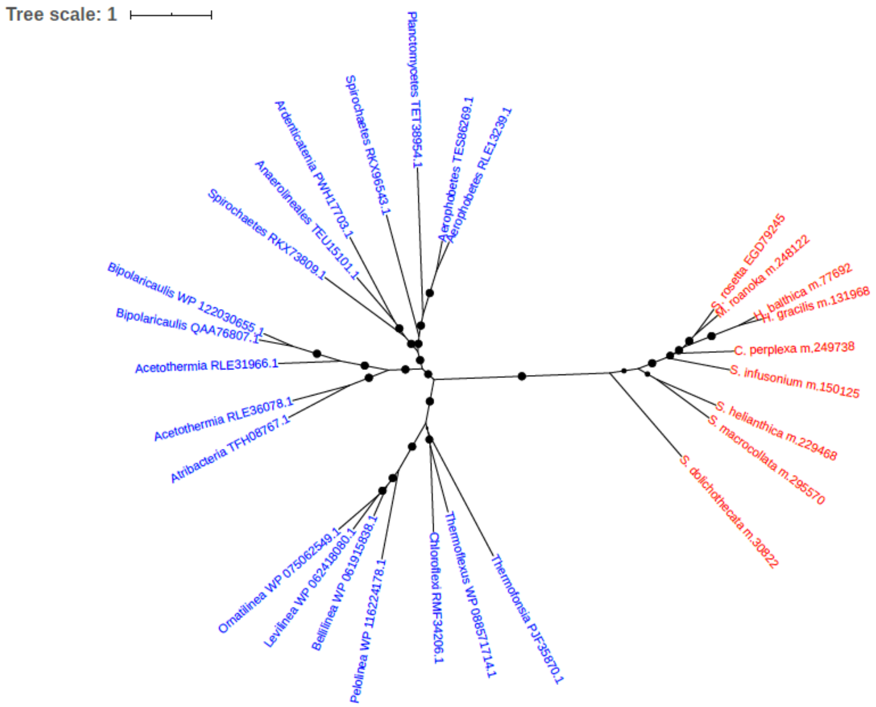

Figure S1-3. Uncharacterized protein (glyA) (EGD79245)

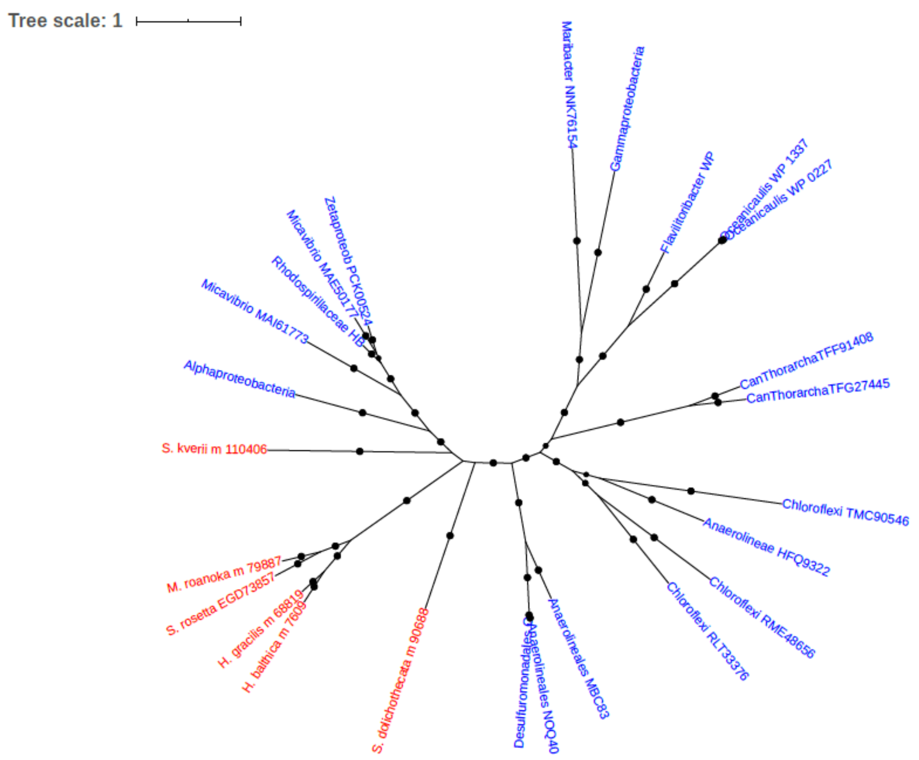

Figure S1-4. Uncharacterized protein (EGD73857)

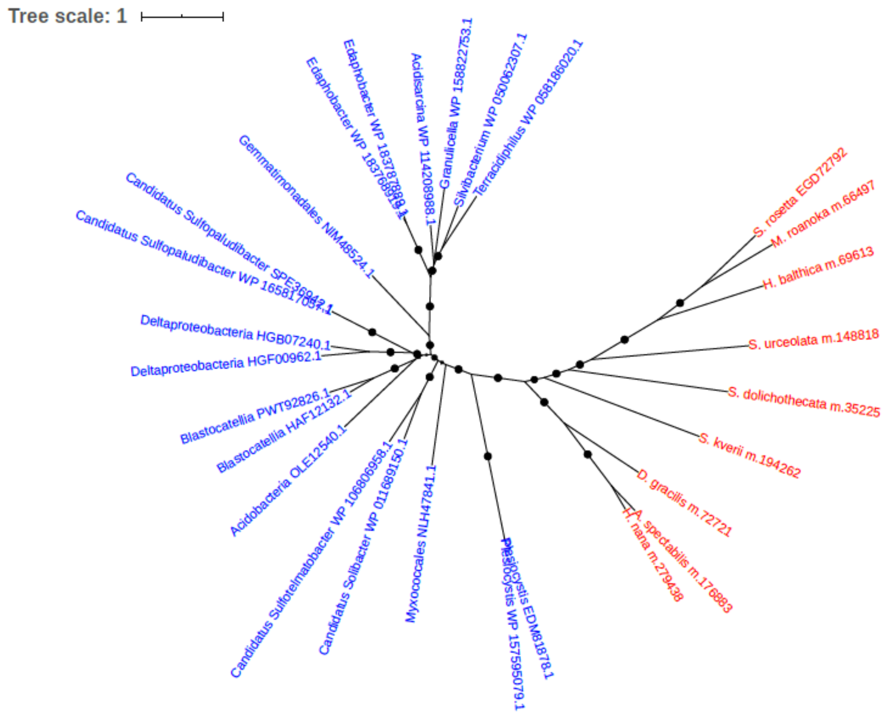

Figure S1-5. Uncharacterized protein (EGD72792)

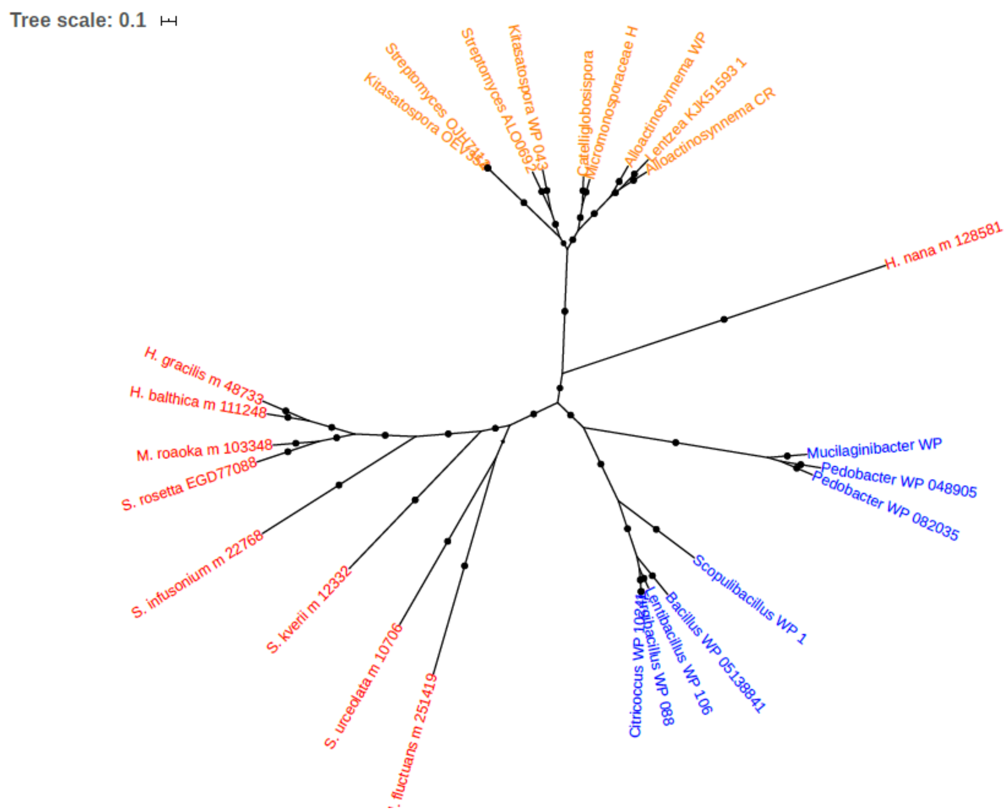

Figure S1-6. Uncharacterized protein (EGD77088)

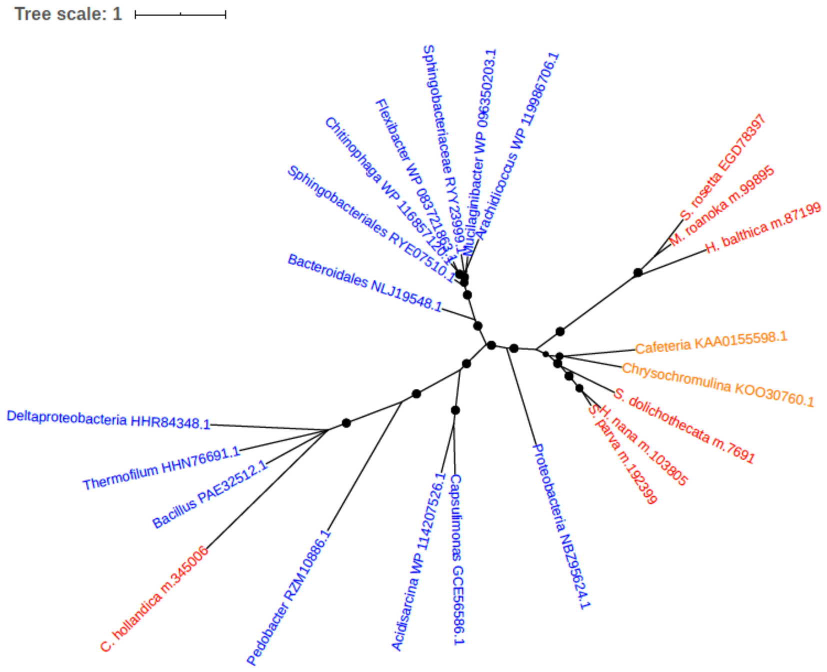

Figure S1-7. Uncharacterized protein (EGD78397)

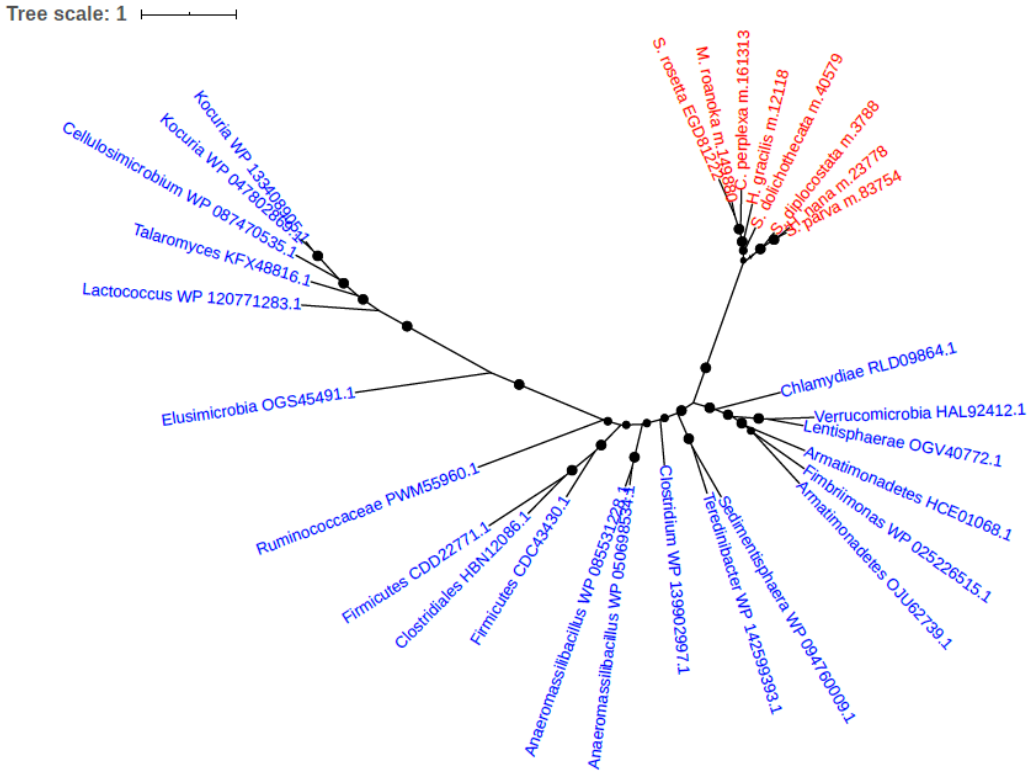

Figure S1-8. Uncharacterized protein (EGD81222)

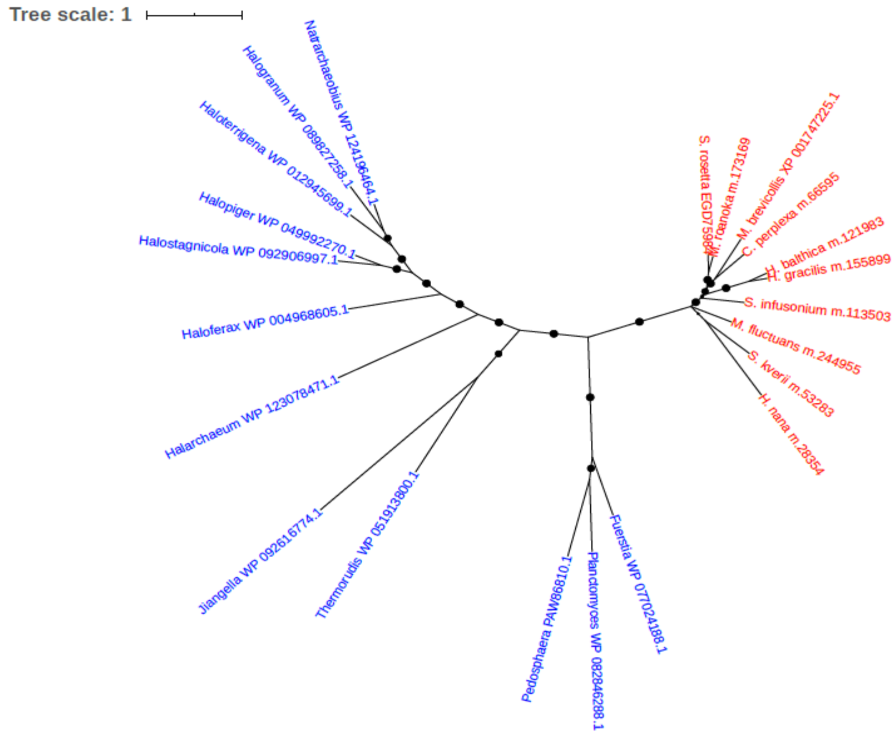

Figure S1-9. Mannonate dehydratase (EGD75984)

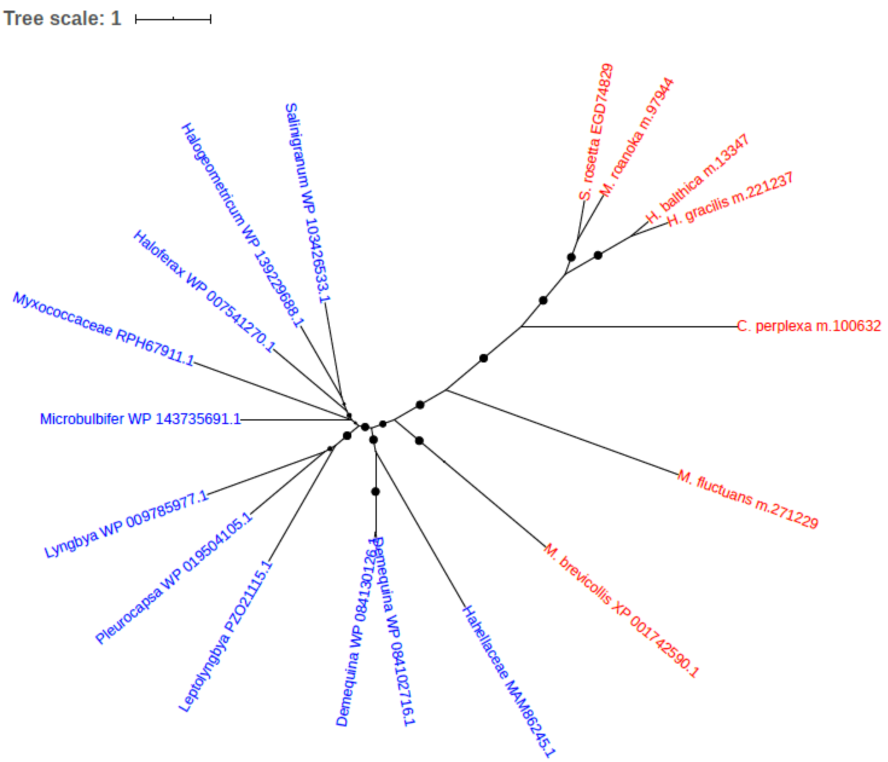

Figure S1-10. Uncharacterized protein (EGD74829)

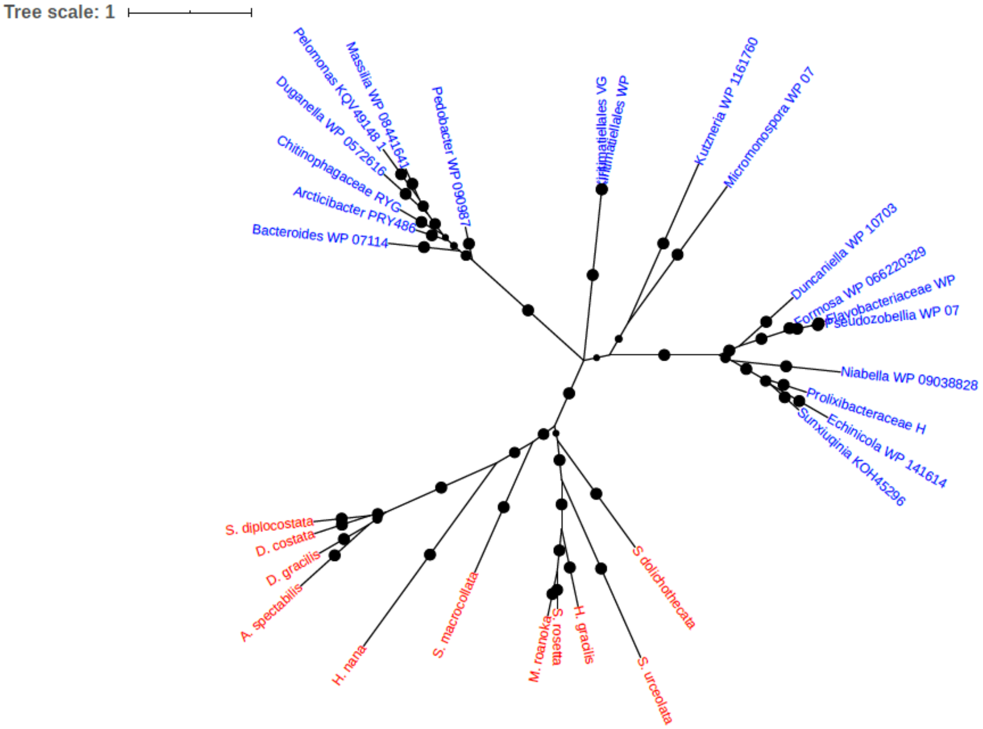

Figure S1-11. Glycosyl hydrolase, family 88 (EGD81375)

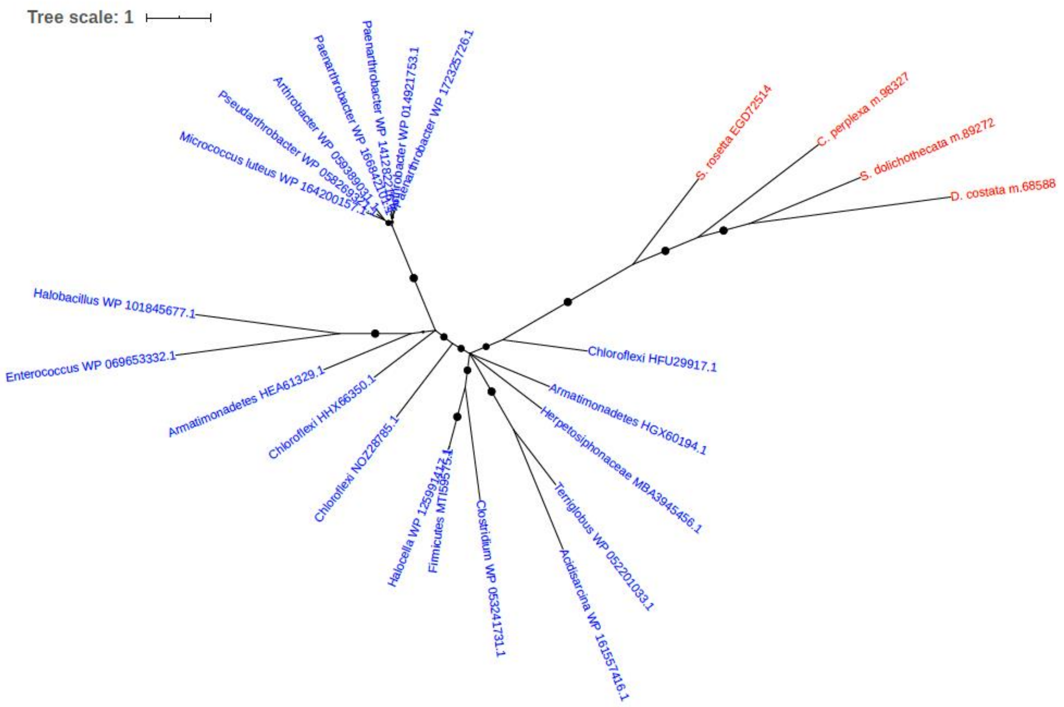

Figure S1-12. Uncharacterized protein (EGD72514)

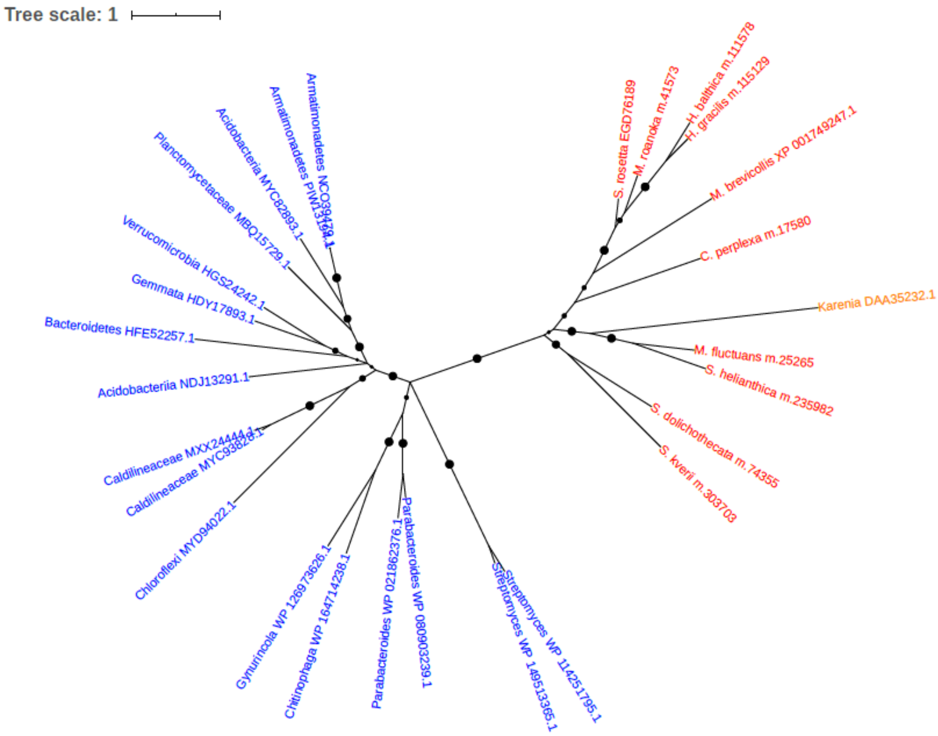

Figure S1-13. Exo-alpha-sialidase (EGD76189)

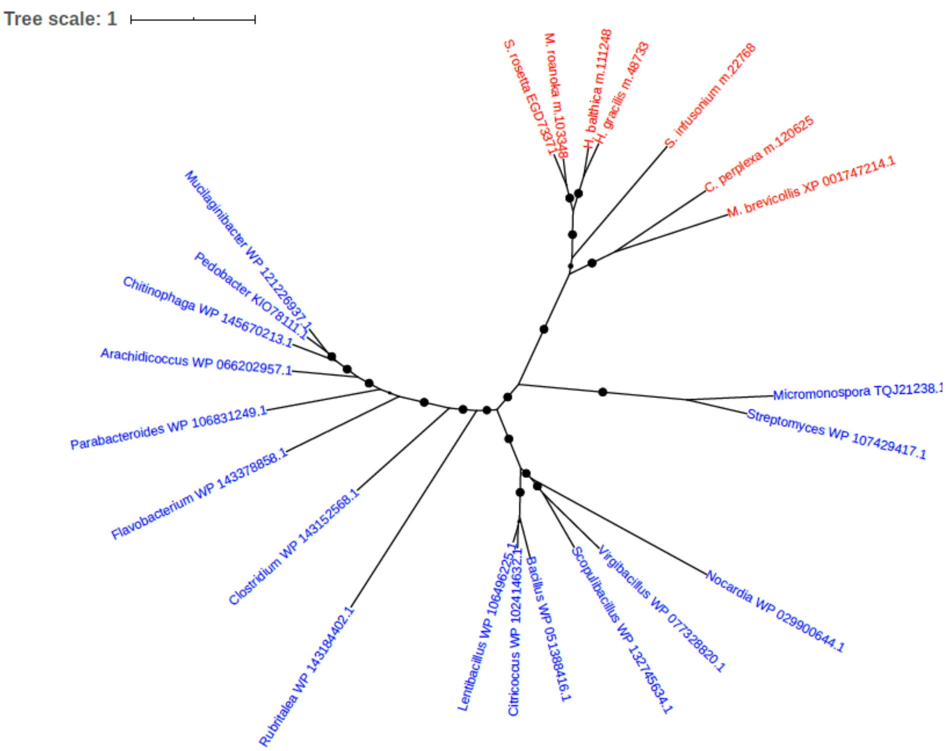

Figure S1-14. Uncharacterized protein (Fragment) (EGD73371)

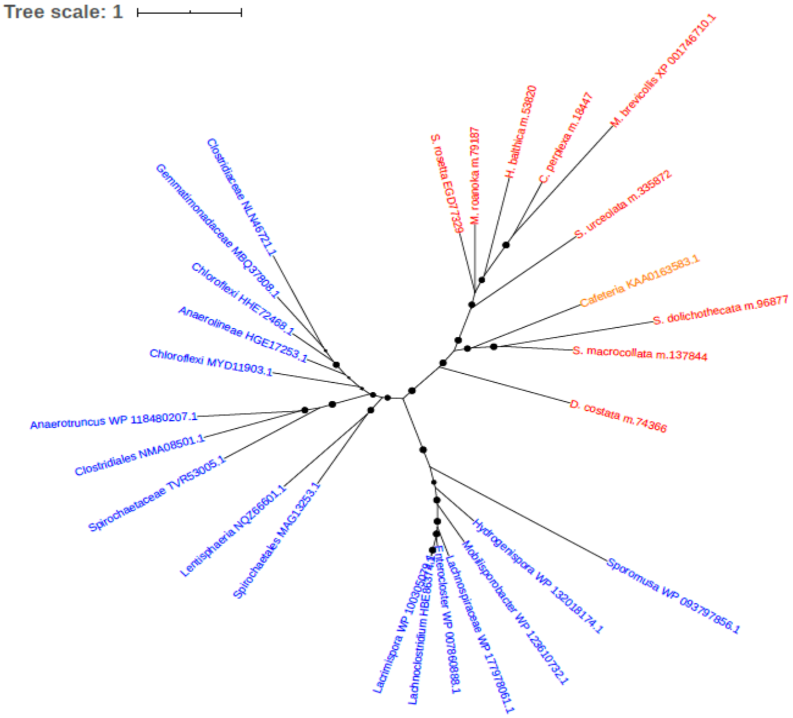

Figure S1-15. Uncharacterized protein (EGD77329)

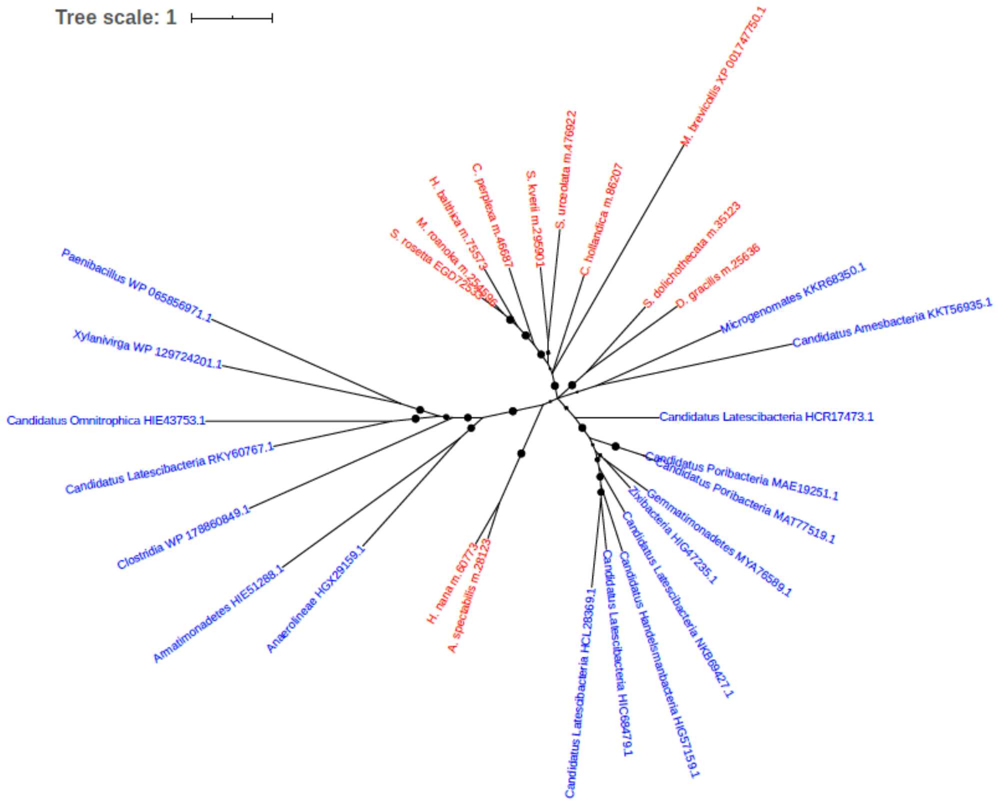

Figure S1-16. Uncharacterized protein (EGD72533)

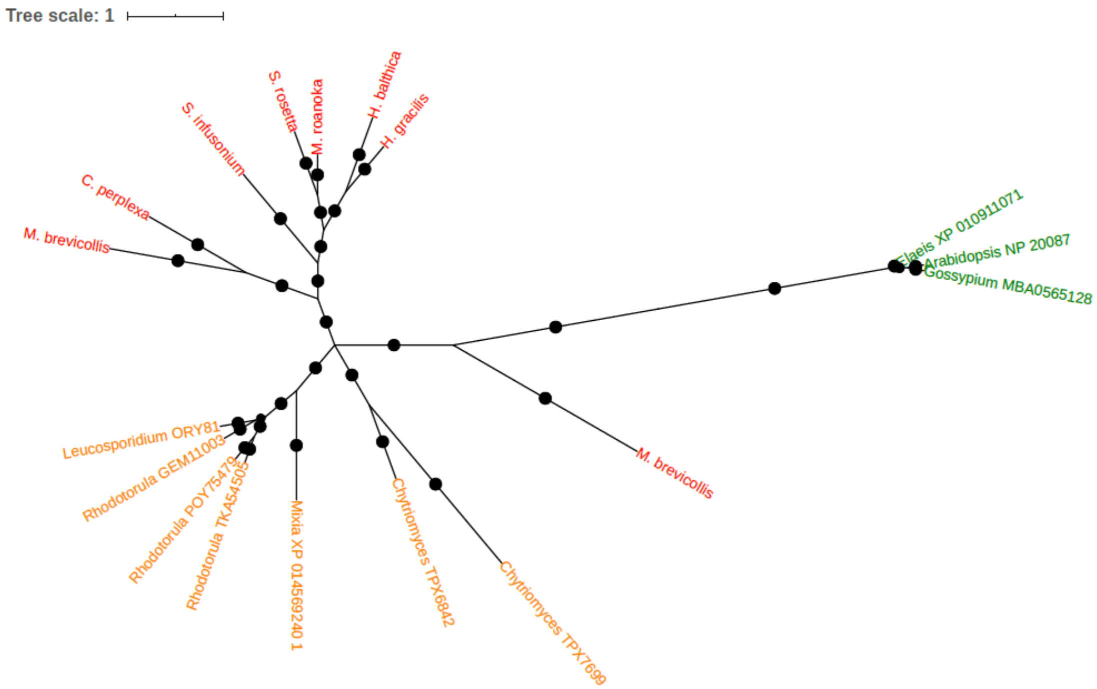

Figure S1-17. Uncharacterized protein (EGD81543)

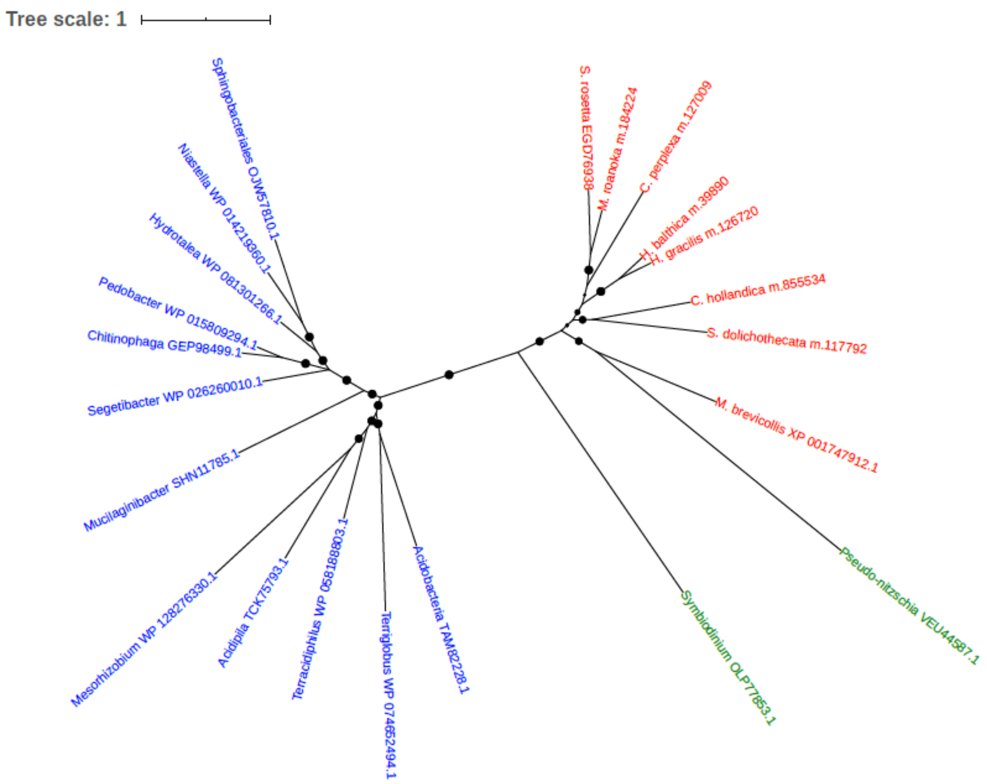

Figure S1-18. Uncharacterized protein (EGD76938)

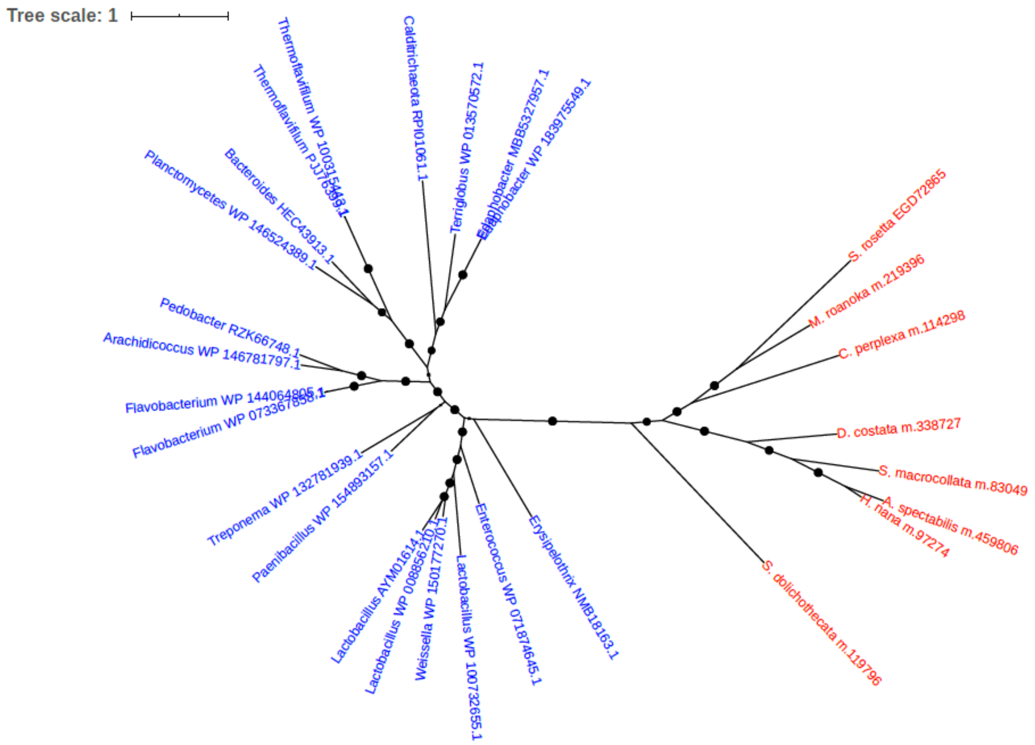

Figure S1-19. Uncharacterized protein (EGD72865)

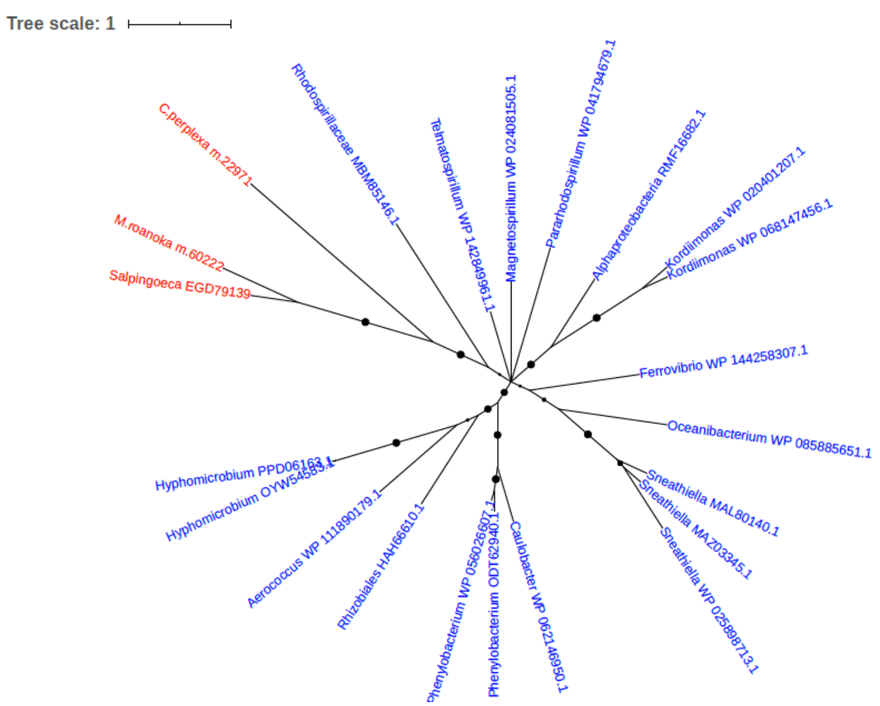

Figure S1-20. NAD+ kinase (EGD79139)

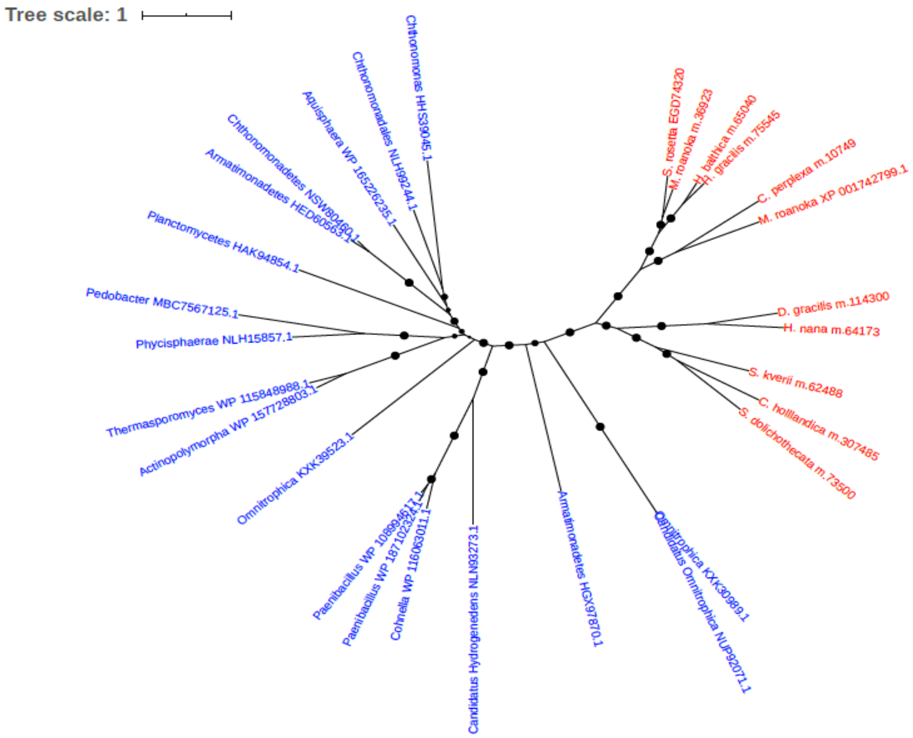

Figure S1-21. Uncharacterized protein (EGD74320)

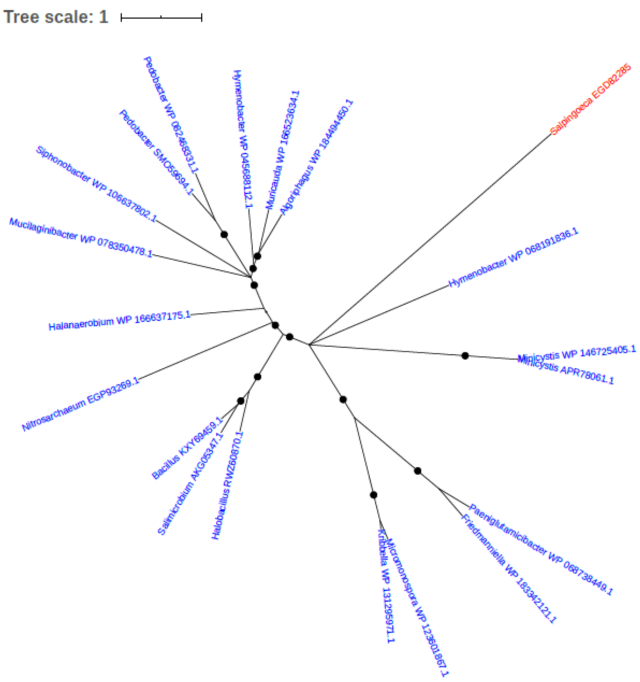

Figure S1-22. Uncharacterized protein (EGD82285)

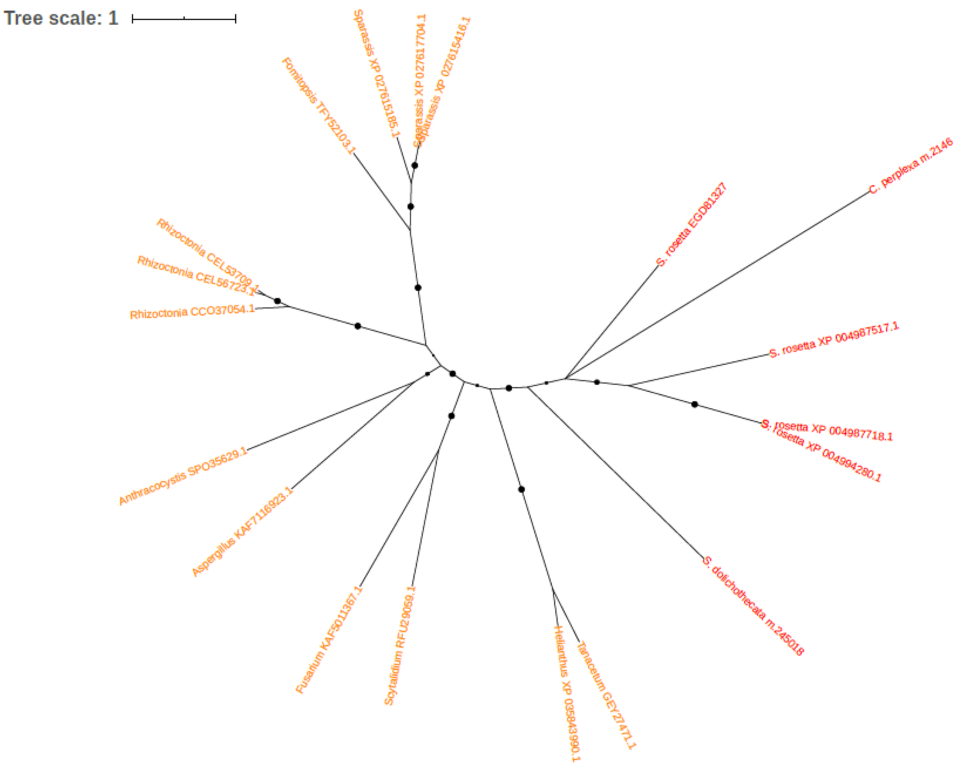

Figure S1-23. Uncharacterized protein (EGD81327)

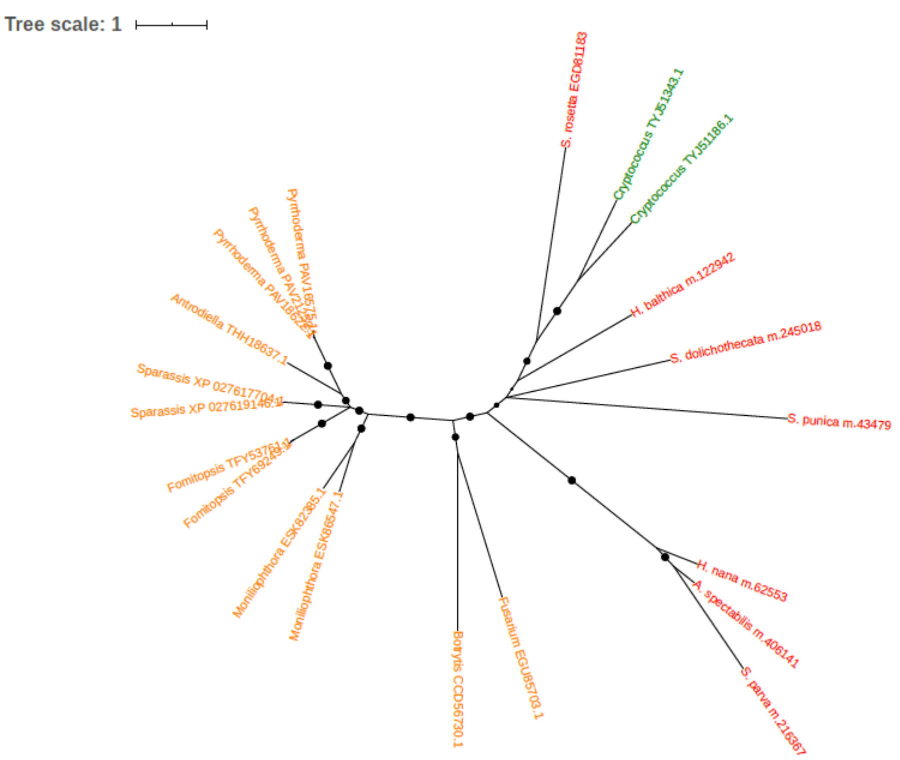

Figure S1-24. Uncharacterized protein (EGD81183)

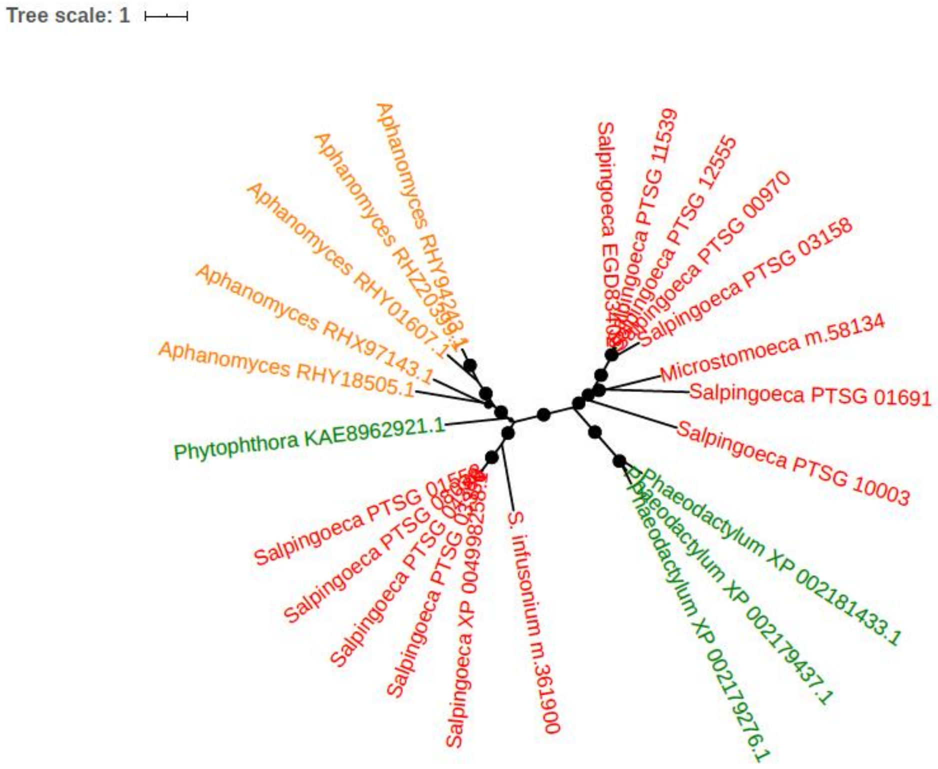

Figure S1-25. Uncharacterized protein (EGD83402)

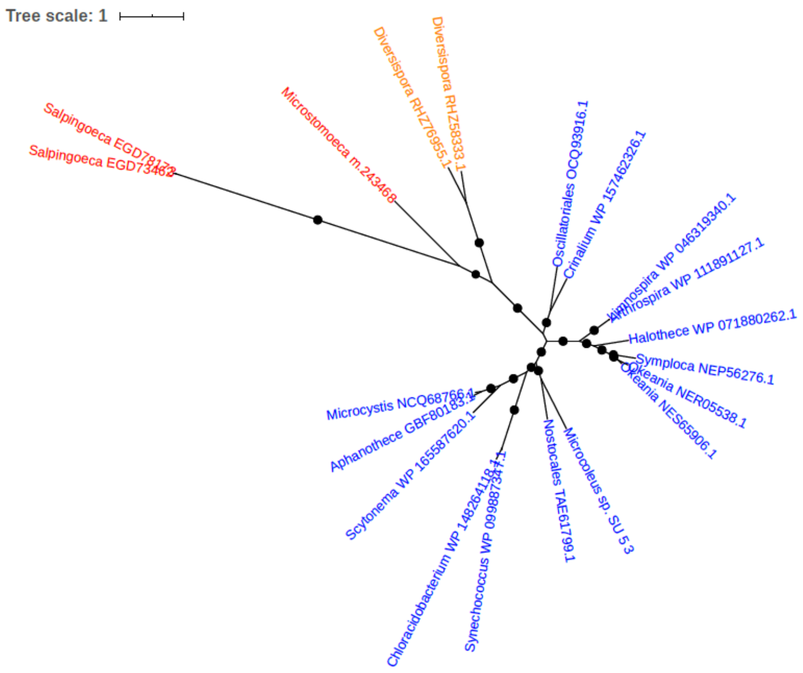

Figure S1-26. Uncharacterized protein (EGD73462 and EGD78172)

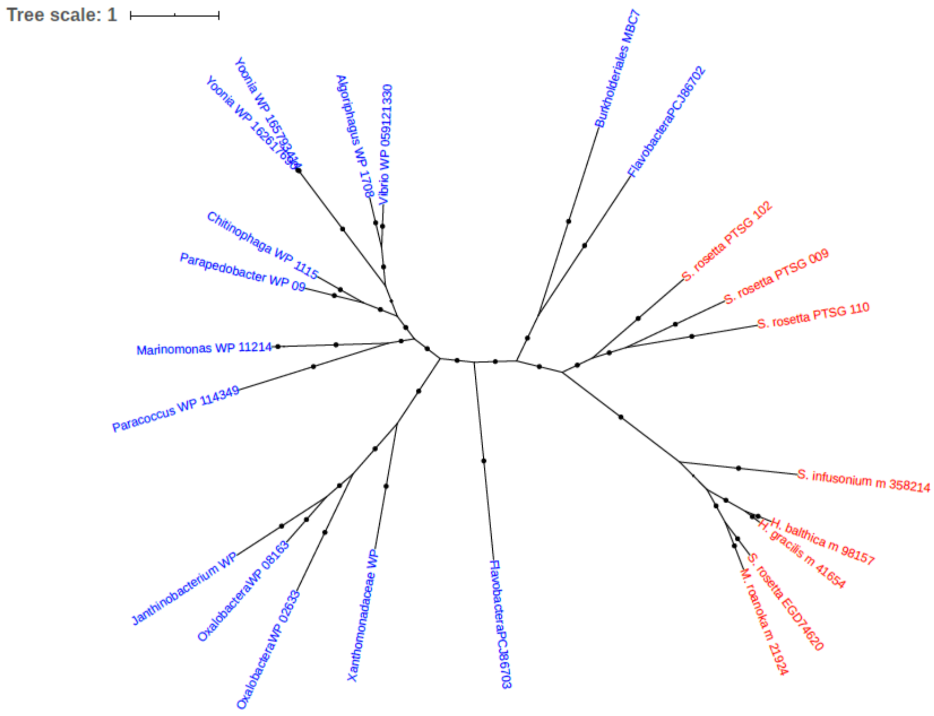

Figure S1-27. Uncharacterized protein (EGD74620 and EGD81083)

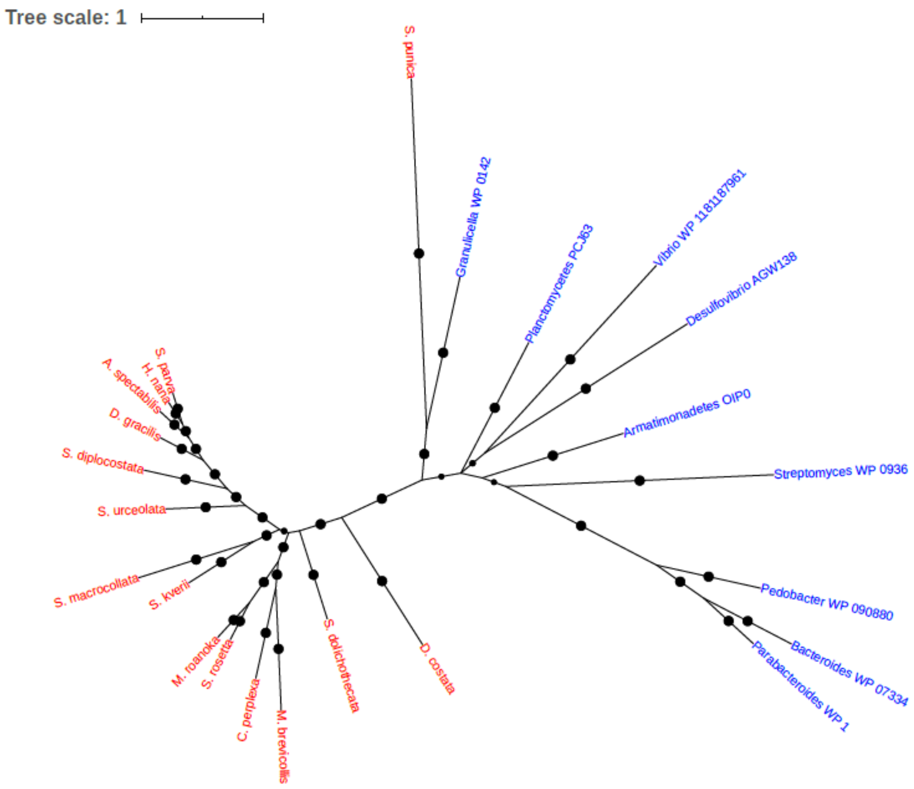

Figure S1-28. Uncharacterized protein (EGD79387)

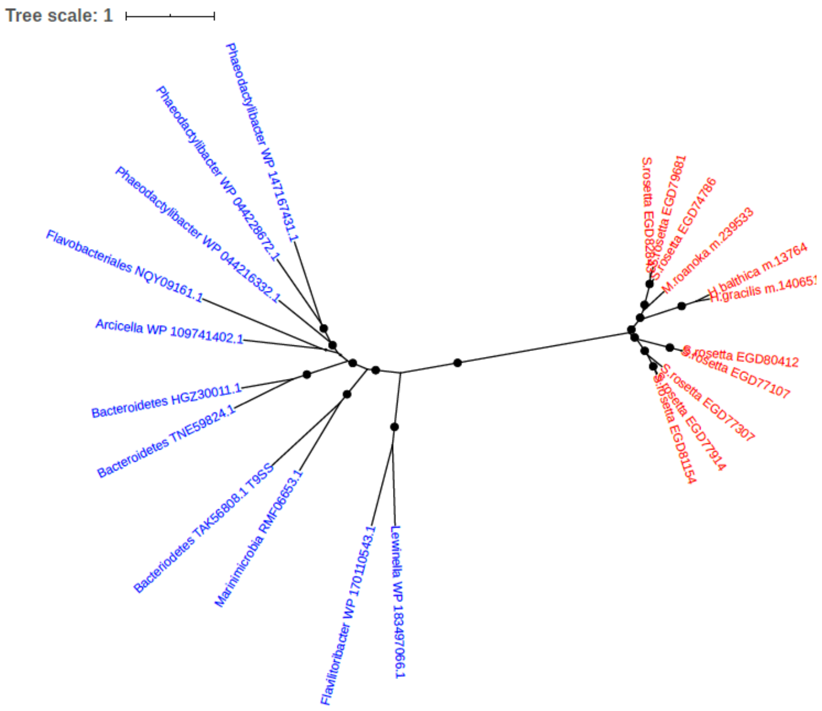

Figure S1-29. Uncharacterized protein (EGD82843, EGD796831, EGD74786, EGD80412, EGD77107, EGD77307, EGD77914, and EGD81154)

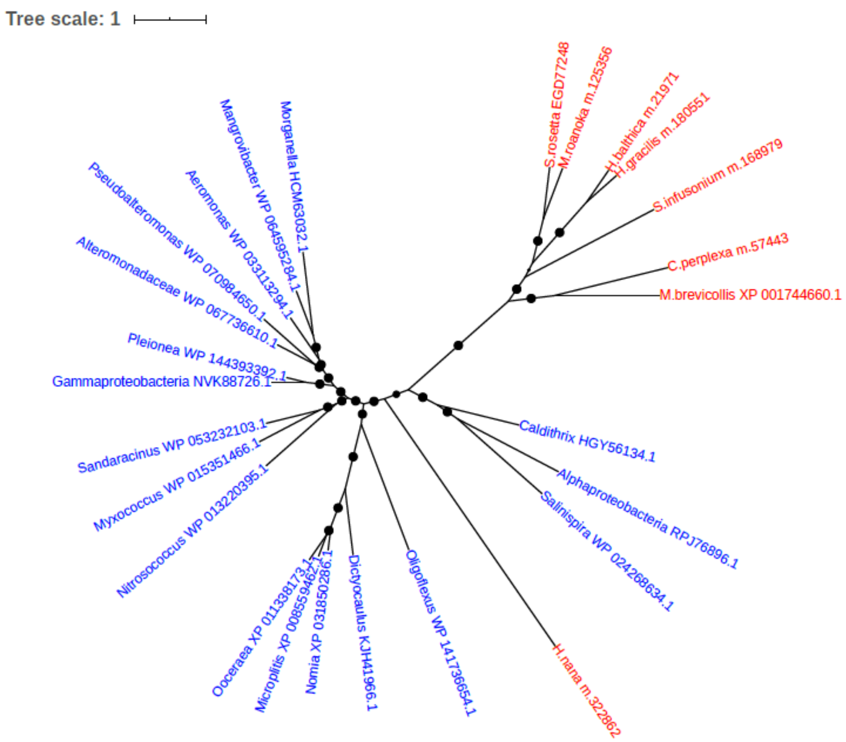

Figure S1-30. 3-hydroxybutyryl-CoA dehydratase (EGD77248)

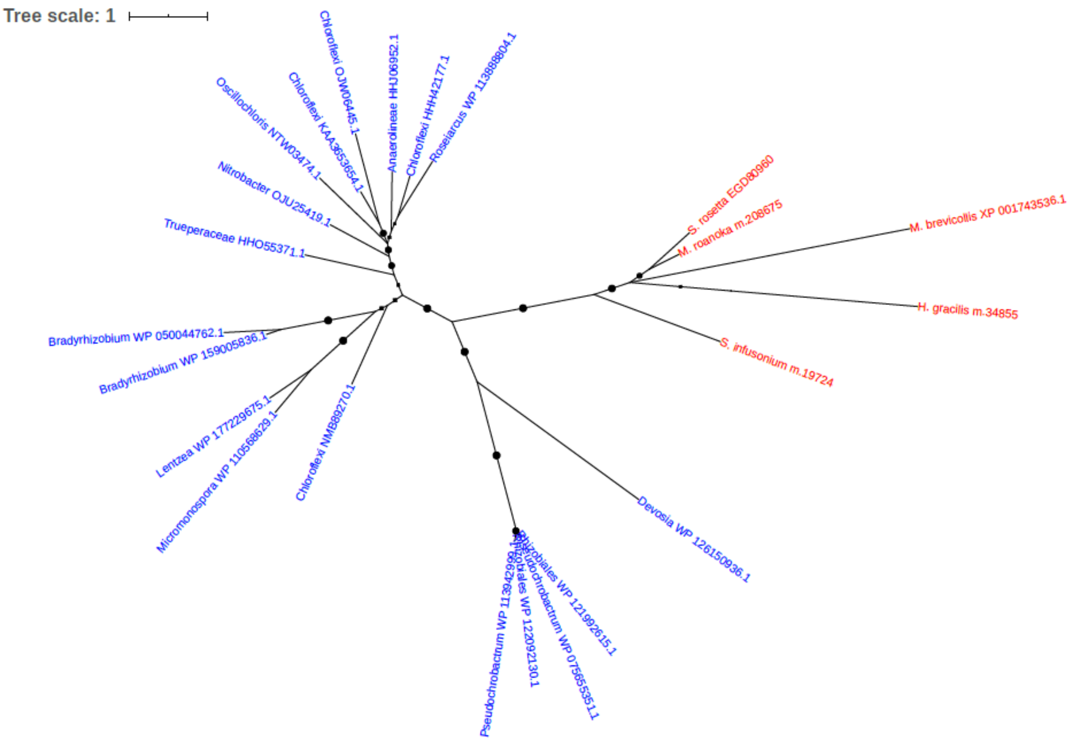

Figure S1-31. Uncharacterized protein (EGD80960)

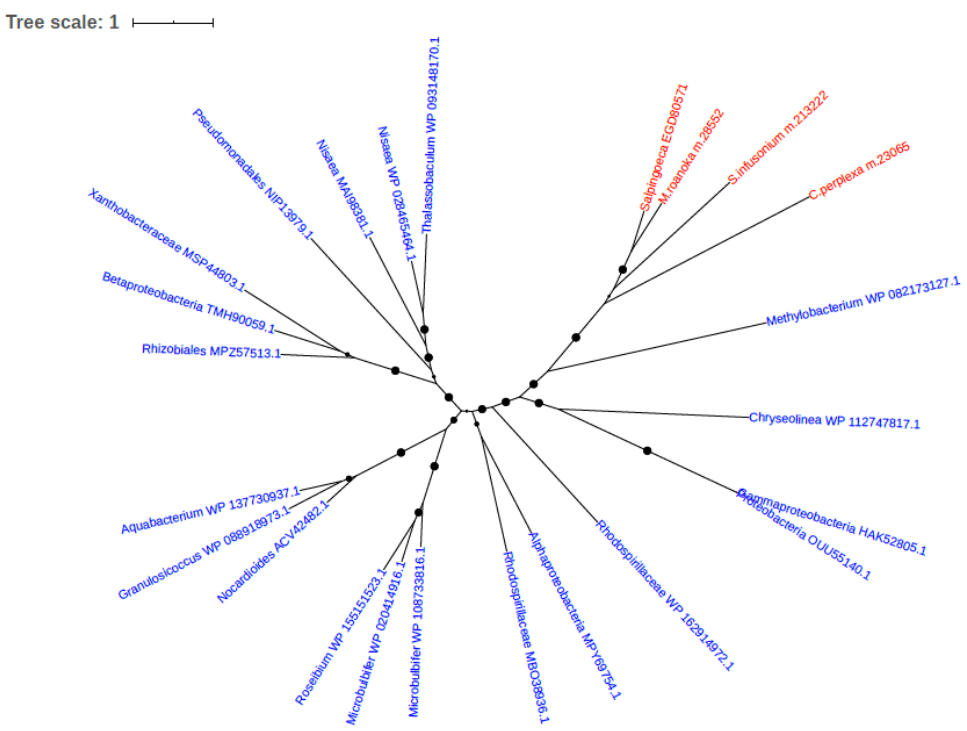

Figure S1-32. Esterase (EGD80571)

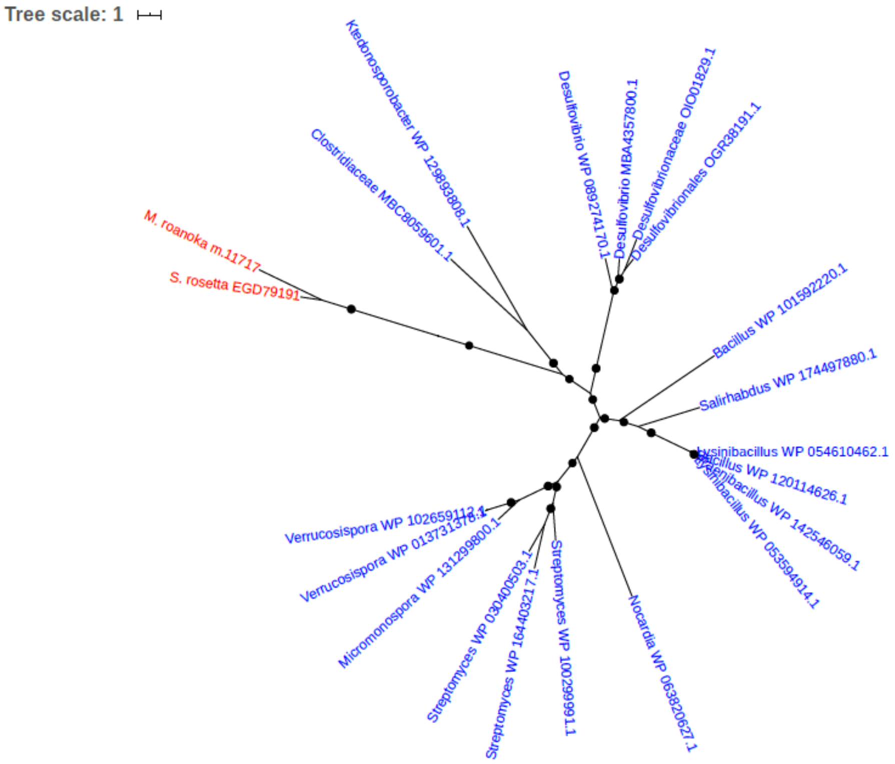

Figure S1-33. Uncharacterized protein (EGD79191)

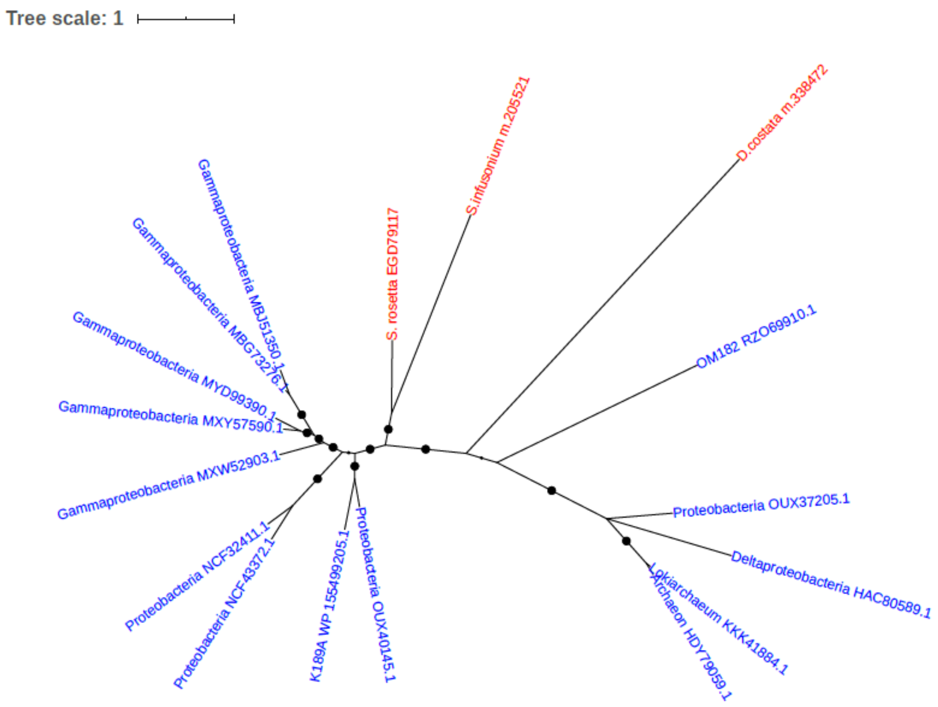

Figure S1-34. Dihydroflavonone isomerase (EGD79117)

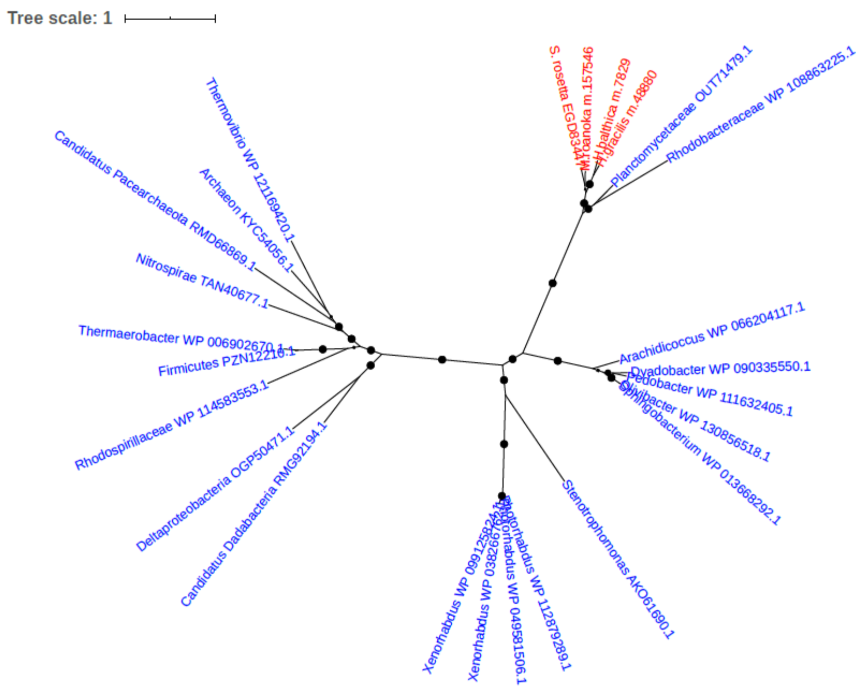

Figure S1-35. Myo-inositol-1-phosphate synthase (EGD83447)

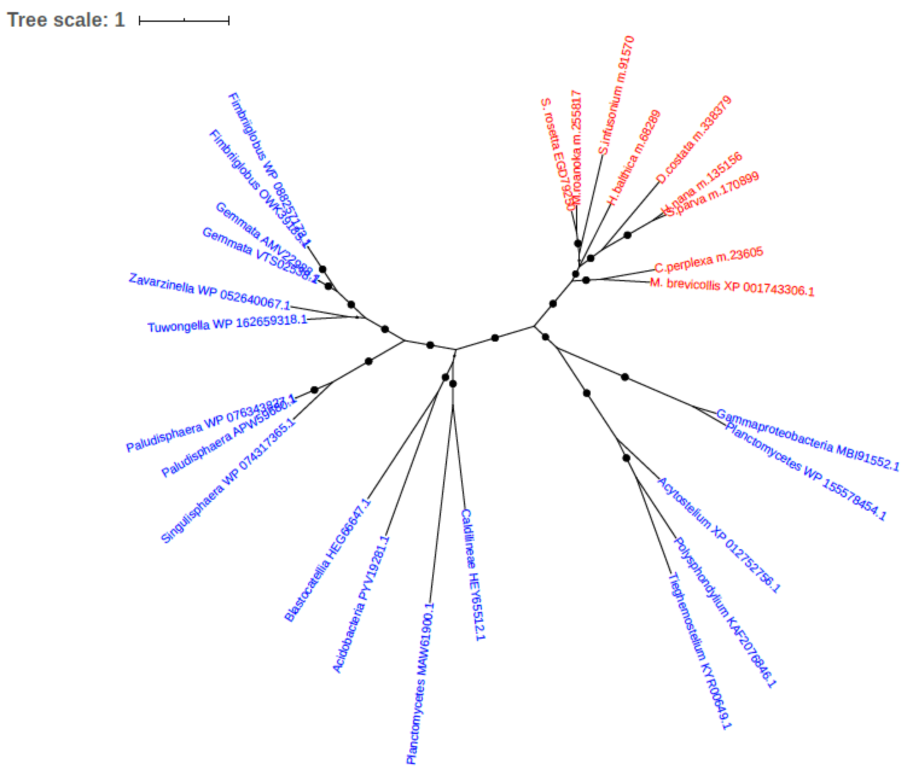

Figure S1-36. NAD-dependent epimerase/dehydratase (EGD79250)

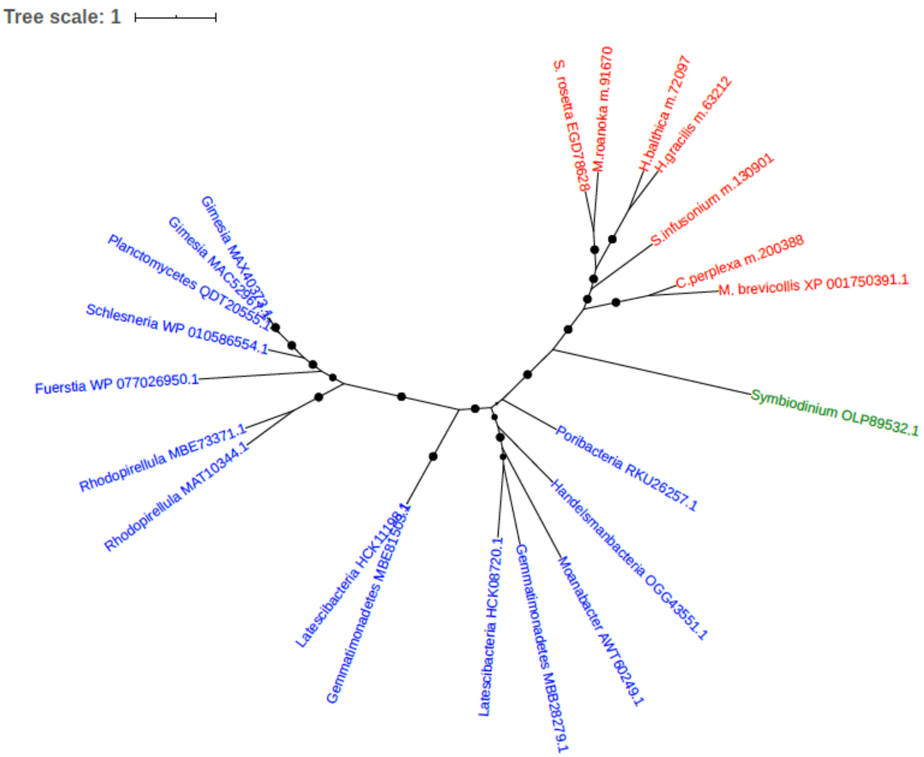

Figure S1-37. Uncharacterized protein (EGD78628)

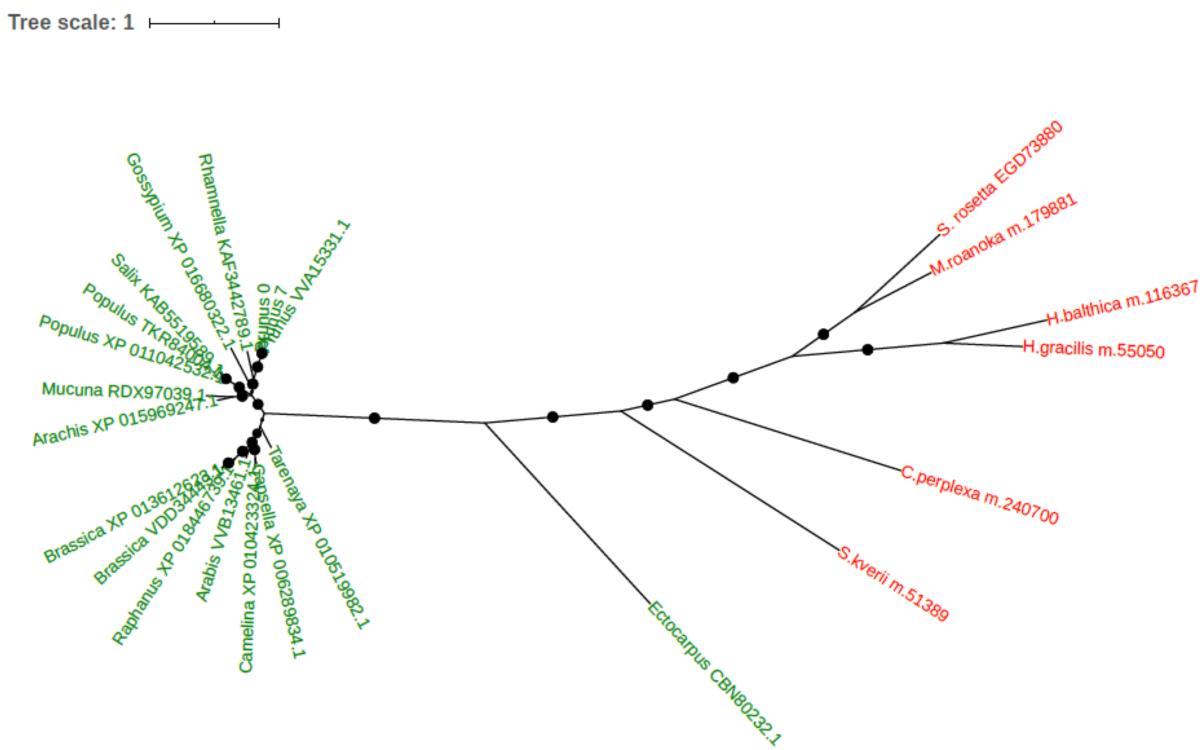

Figure S1-38. Transferase (EGD73880)

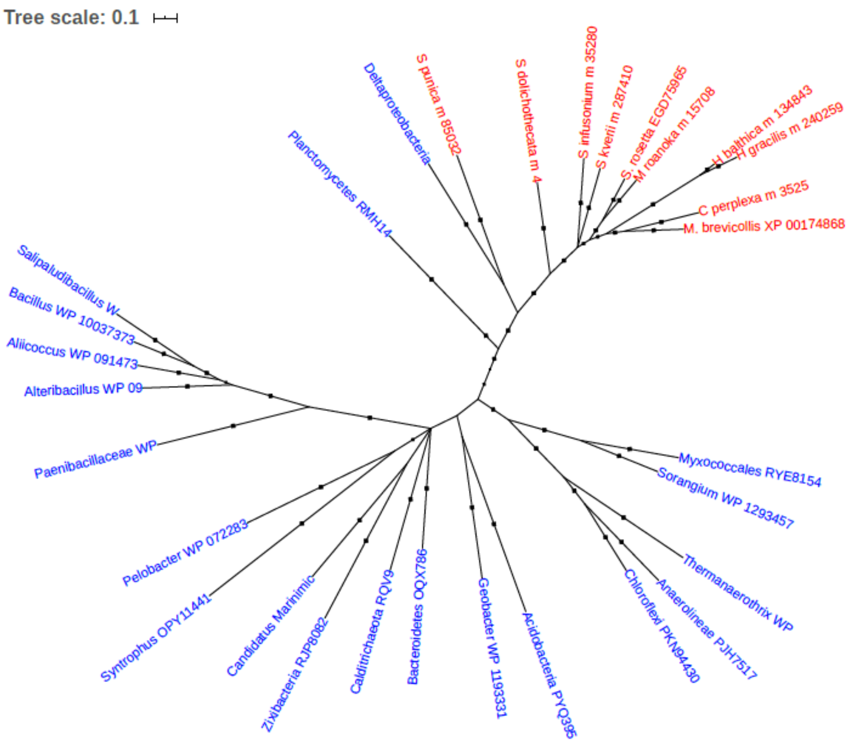

Figure S1-39. Isocitrate dehydrogenase [NADP] (EC 1.1.1.42) (EGD75965)

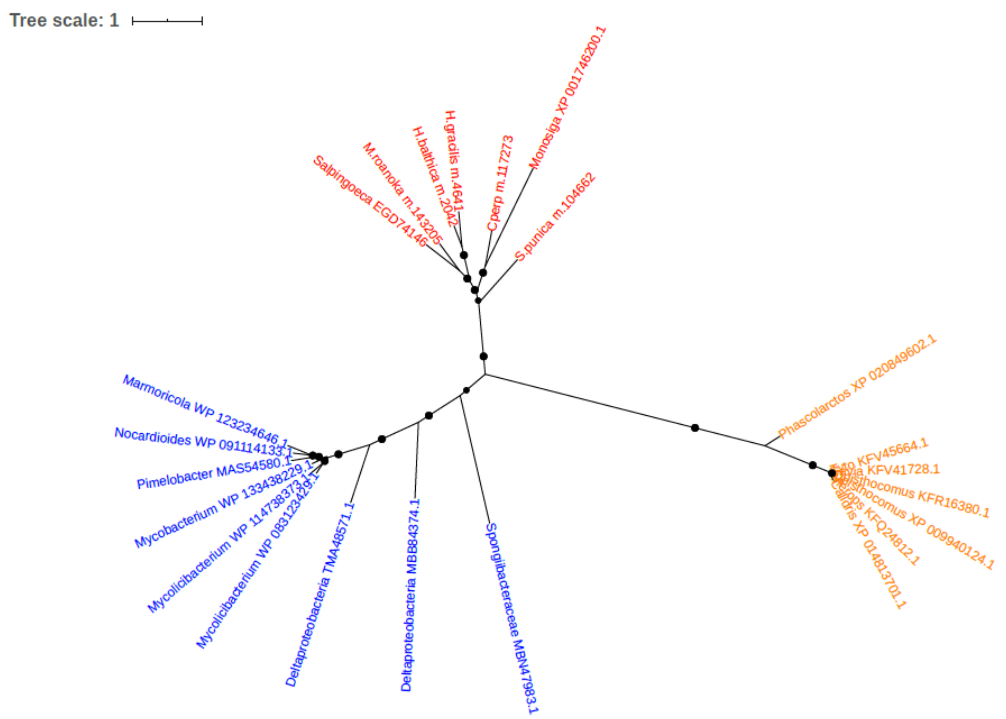

Figure S1-40. Uncharacterized protein (EGD74146)

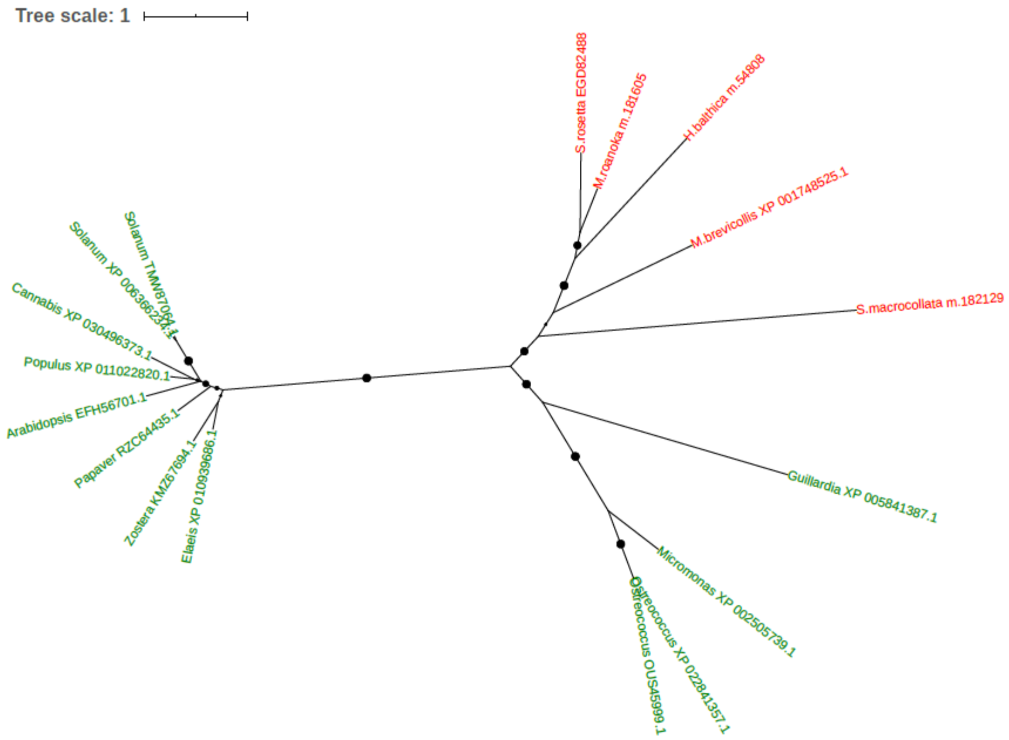

Figure S1-41. Uncharacterized protein (EGD82488)

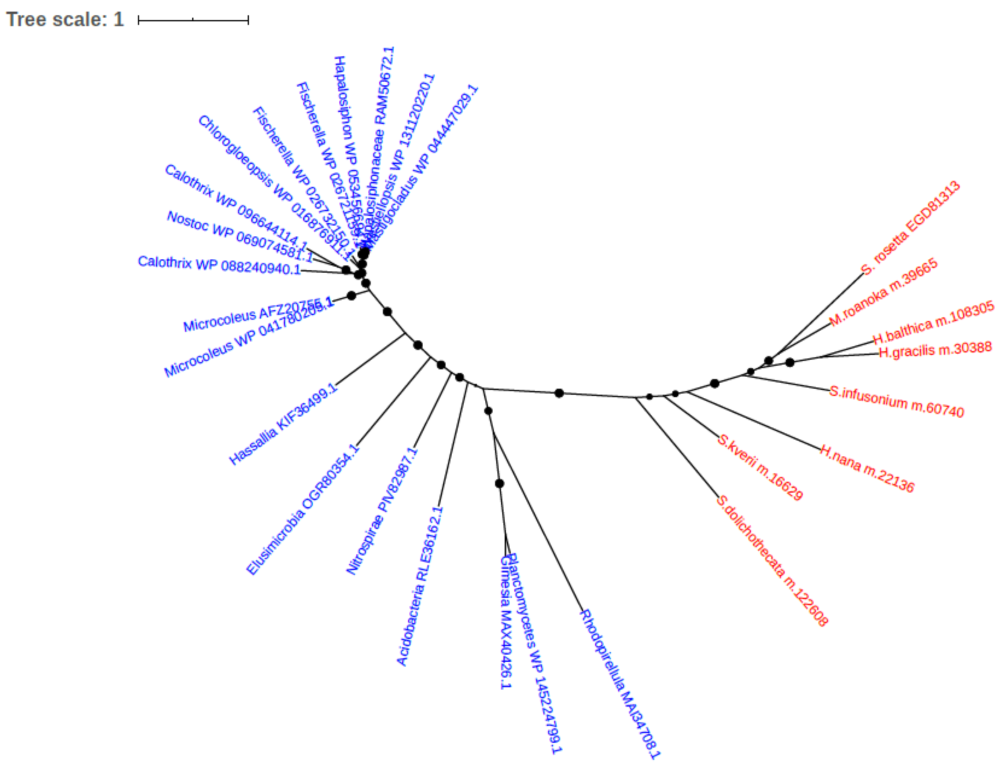

Figure S1-42. Uncharacterized protein (EGD81313)

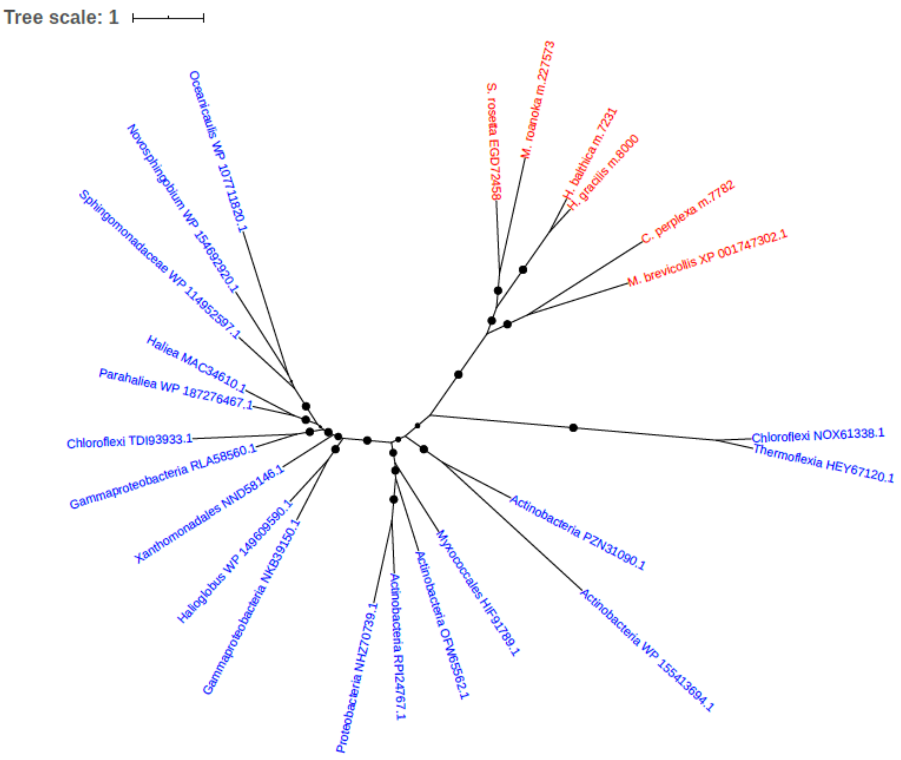

Figure S1-43. Uncharacterized protein (EGD72458)

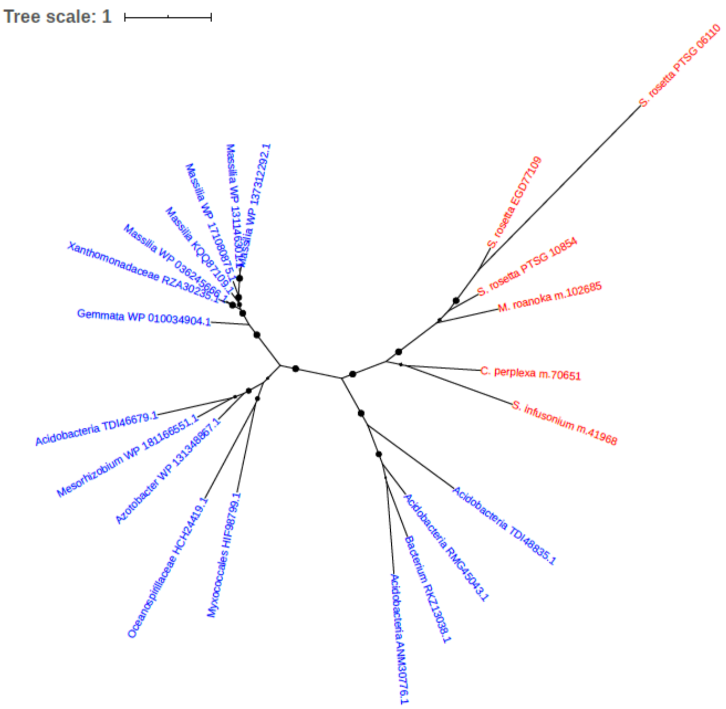

Figure S1-44. Uncharacterized protein (EGD77109)

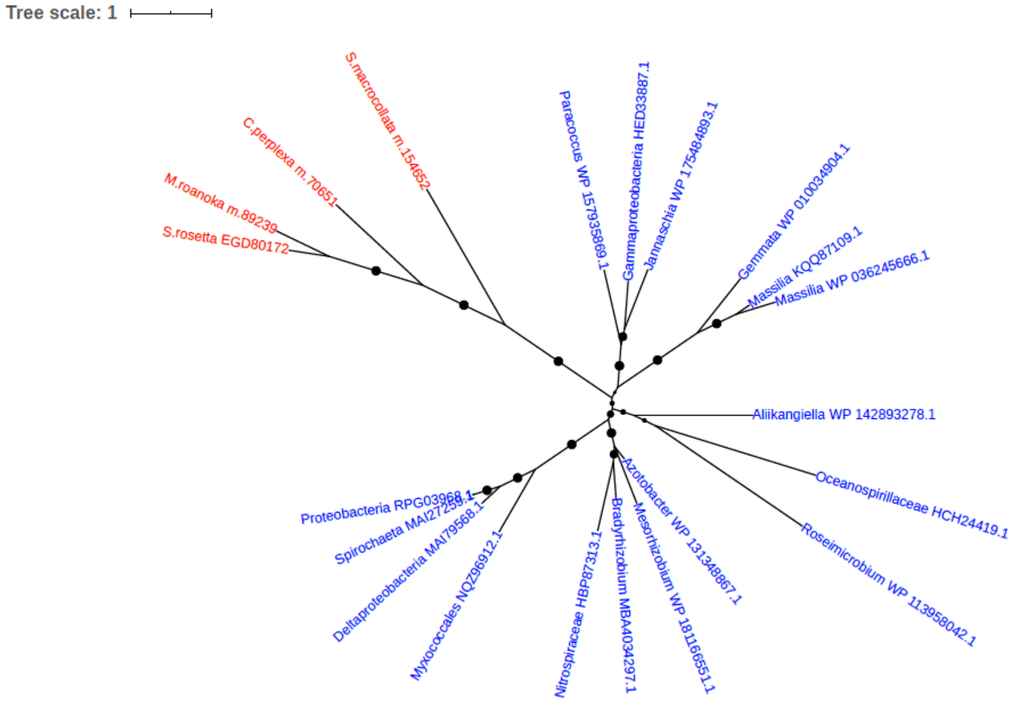

Figure S1-45. Uncharacterized protein (EGD80172)

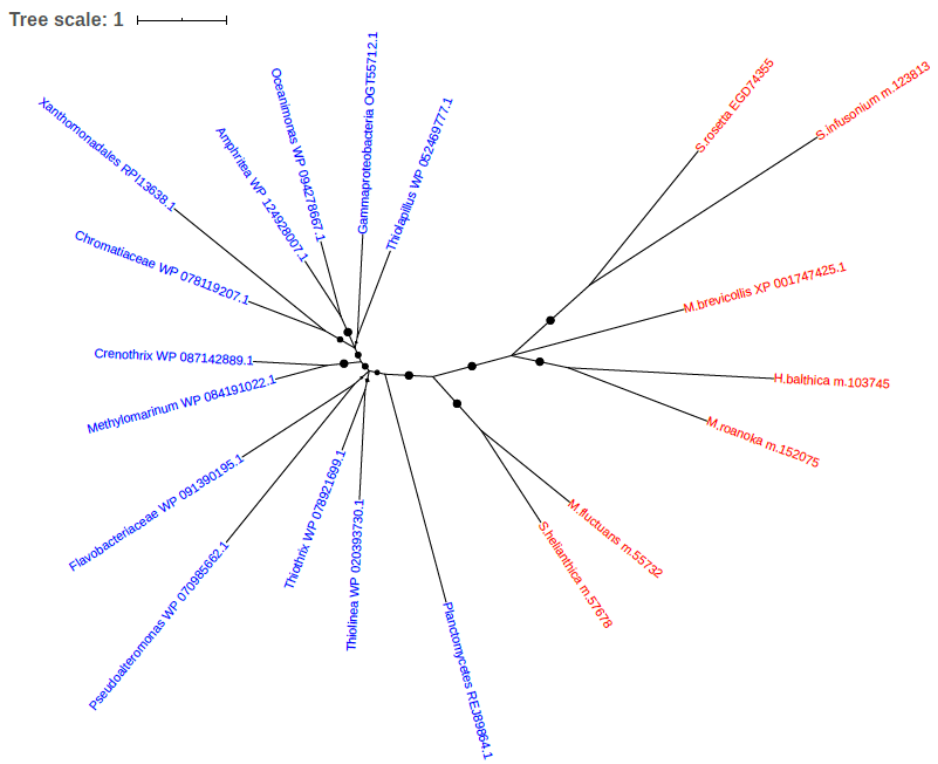

Figure S1-46. Uncharacterized protein (EGD74355)

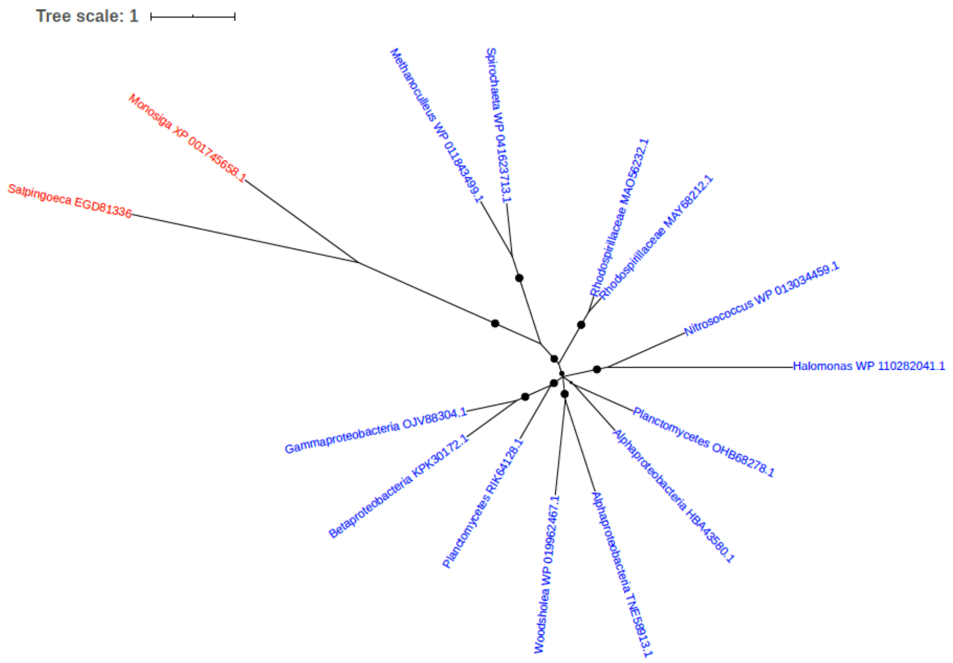

Figure S1-47. Uncharacterized protein (EGD81336)

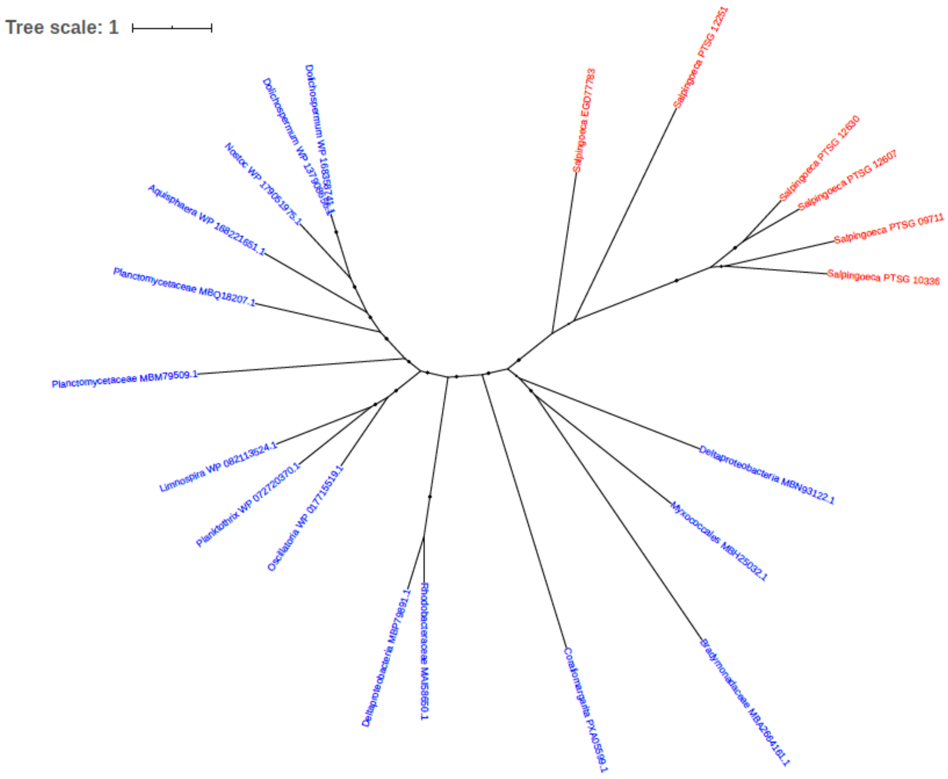

Figure S1-48. Uncharacterized protein (EGD77783)

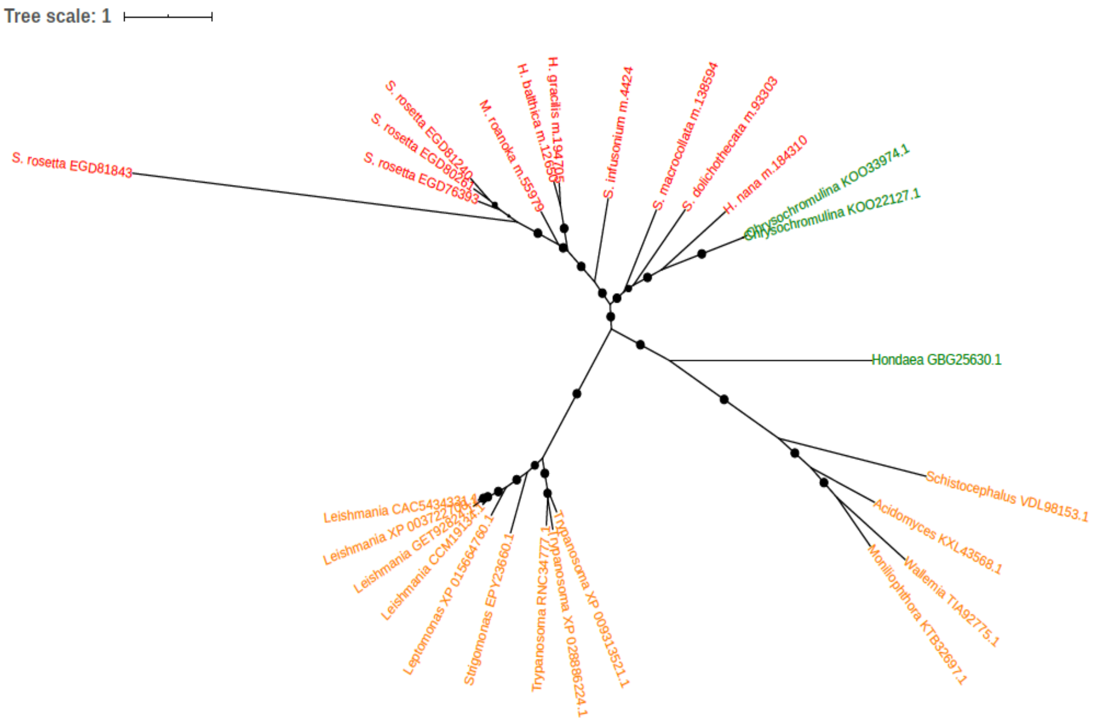

Figure S1-49. Uncharacterized protein (EGD81843, EGD76393, EGD80261, and EGD81240)

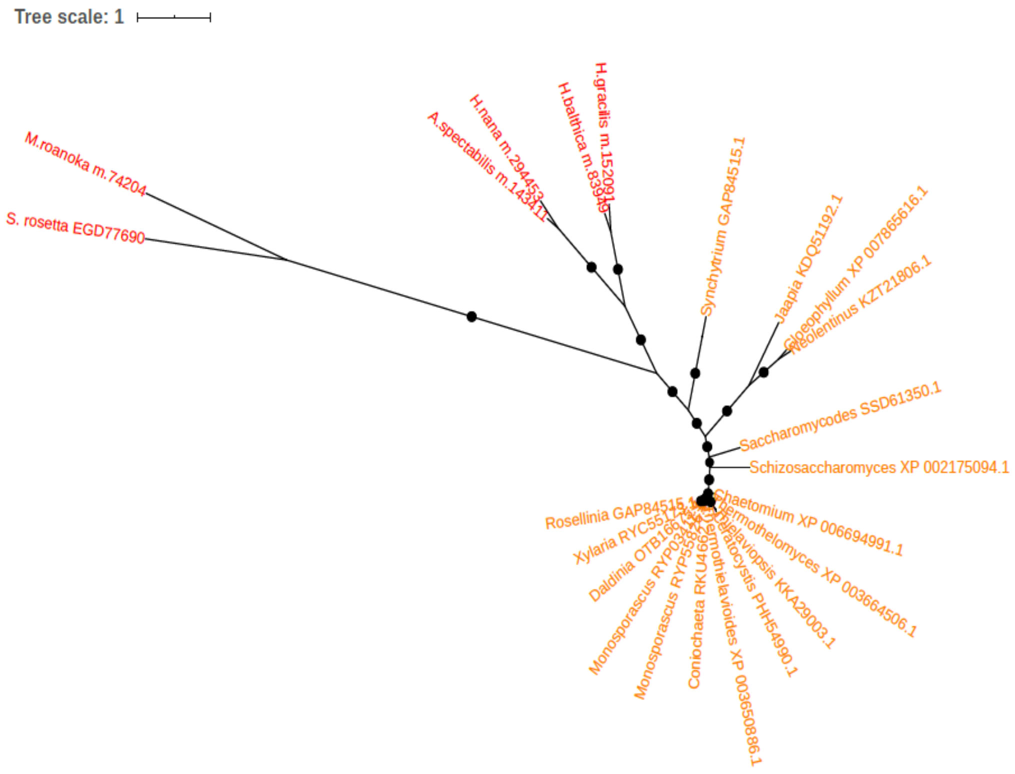

Figure S1-50. Uncharacterized protein (EGD77690)

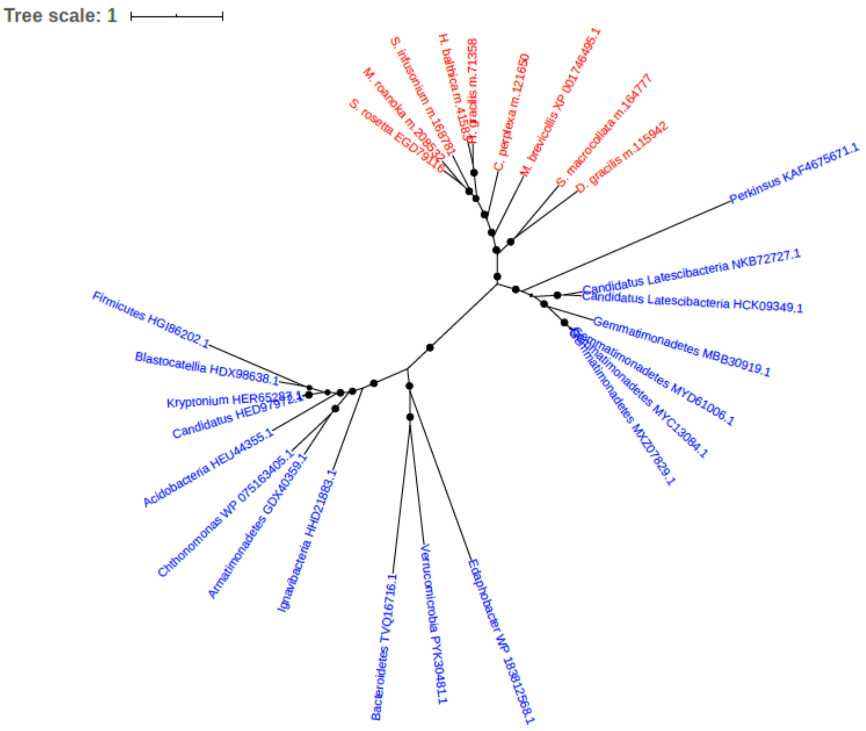

Figure S1-51. Uncharacterized protein (EGD79116)

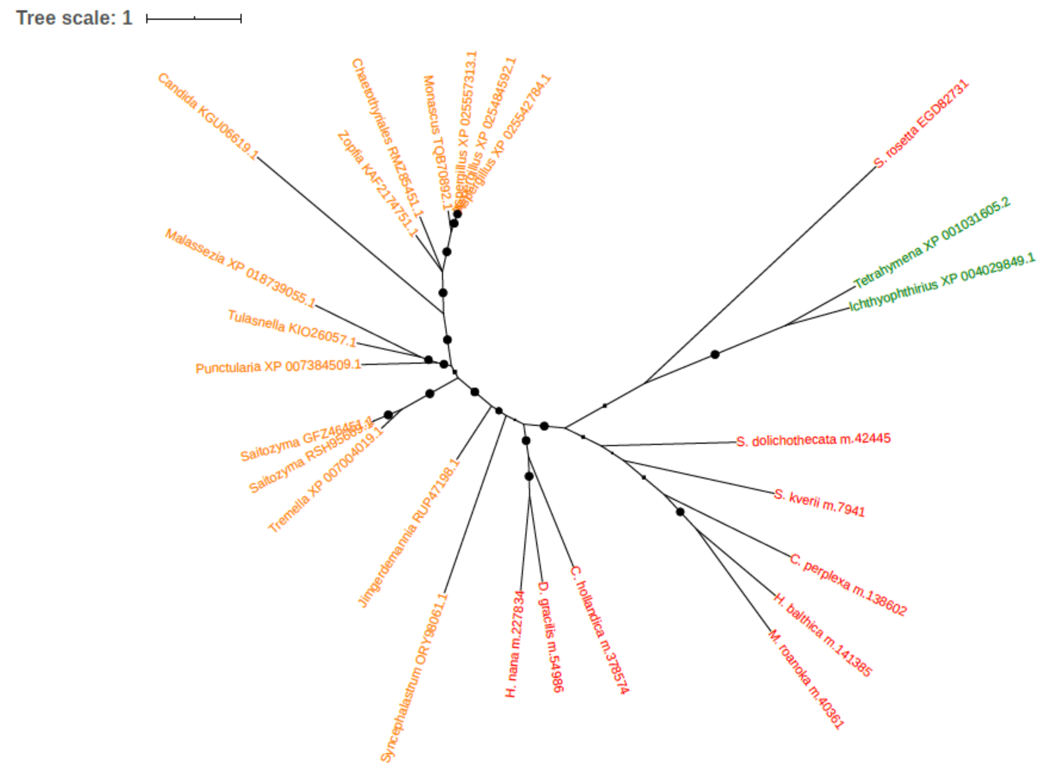

Figure S1-52. Uncharacterized protein (EGD82731)

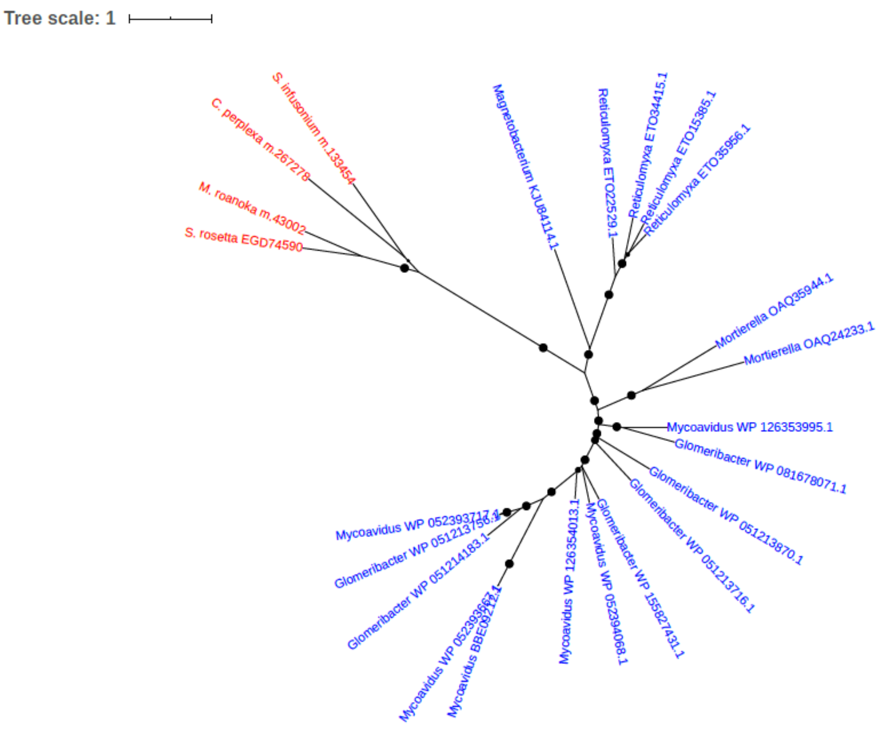

Figure S1-53. Uncharacterized protein (EGD74590)

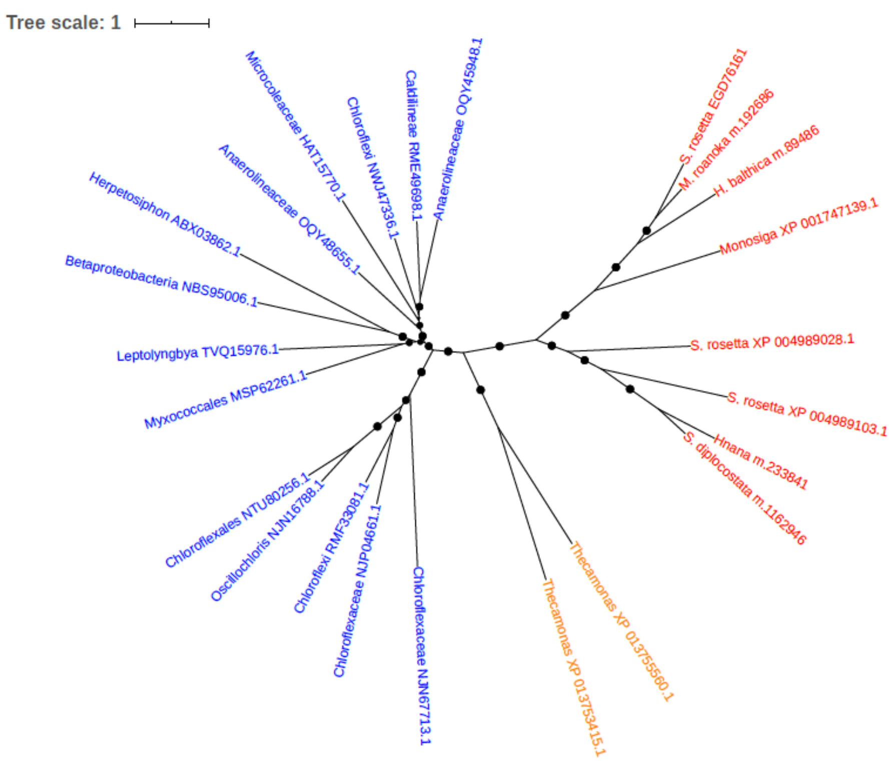

Figure S1-54. Uncharacterized protein (EGD76161)

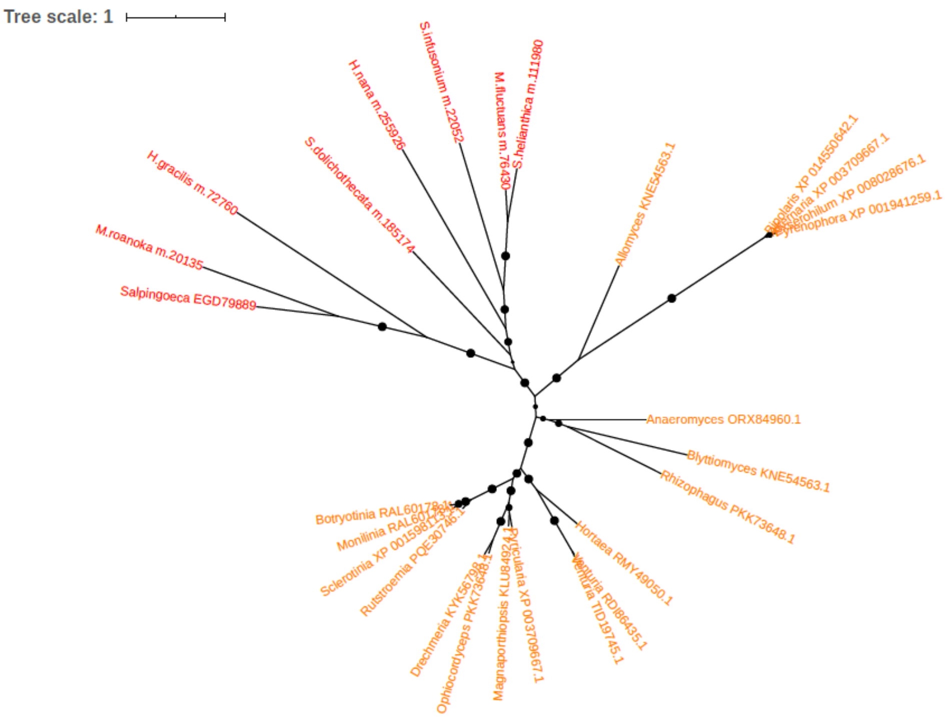

Figure S1-55. CMGC/CDK protein kinase (EGD79889)

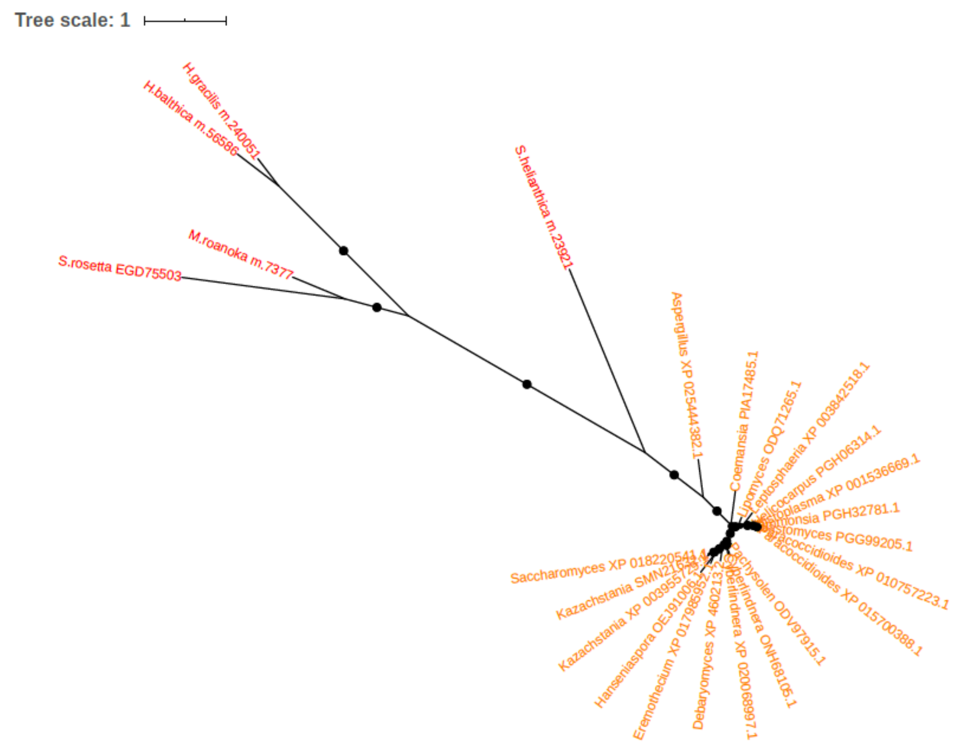

Figure S1-56. CMGC/MAPK protein kinase (EGD75503)

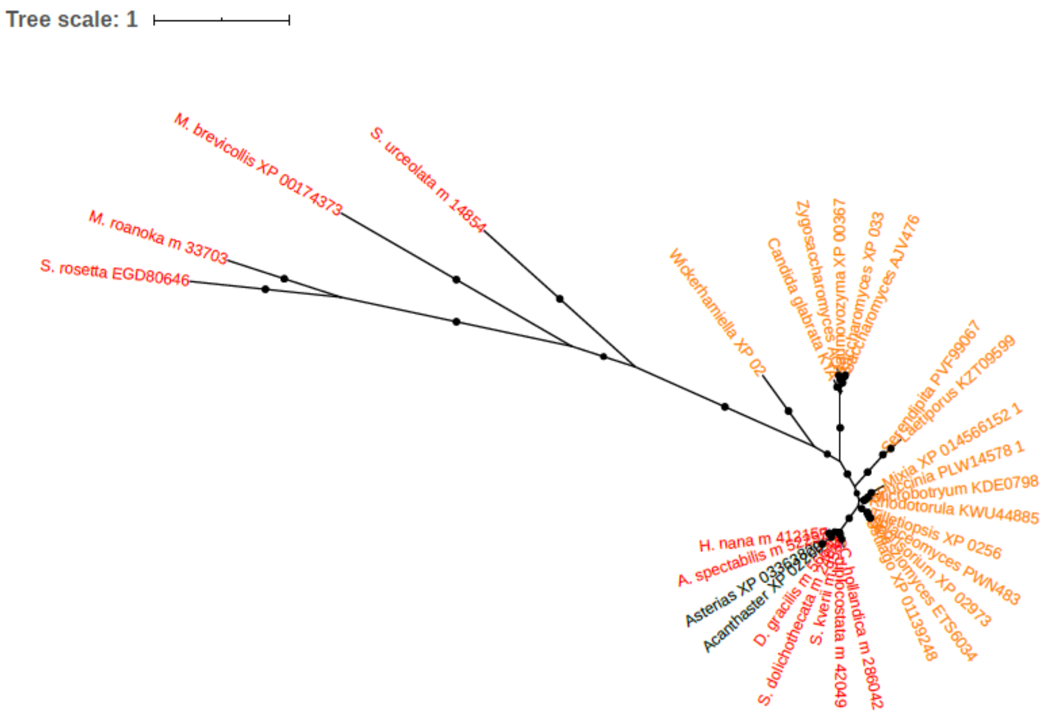

Figure S1-57. Uncharacterized protein (EGD80646)

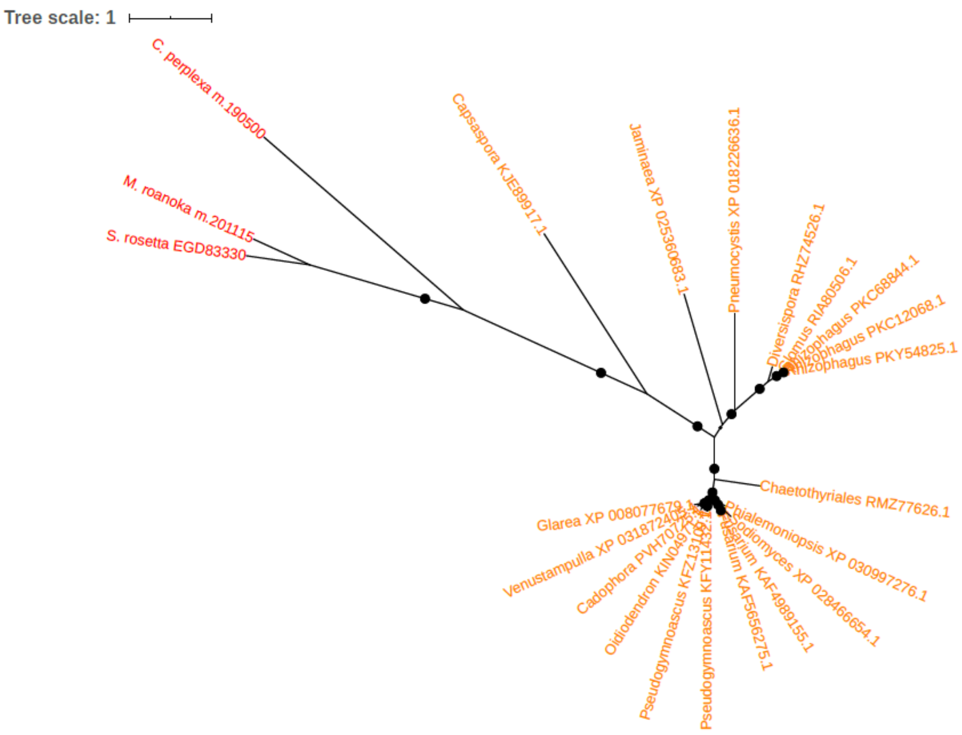

Figure S1-58. Uncharacterized protein (EGD83330)

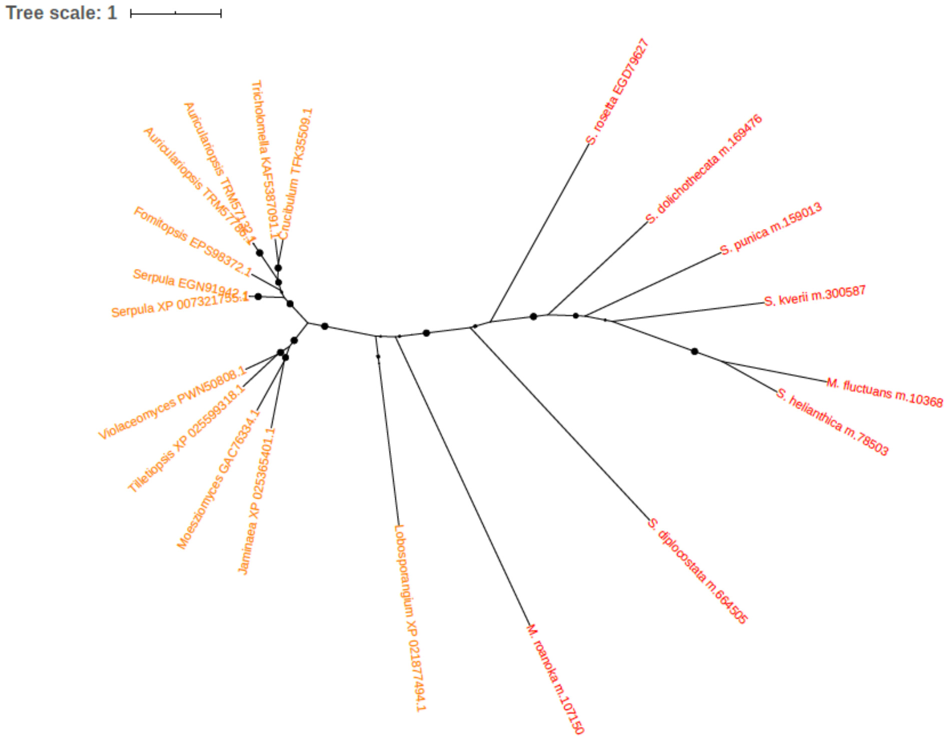

Figure S1-59. Uncharacterized protein (EGD79627)

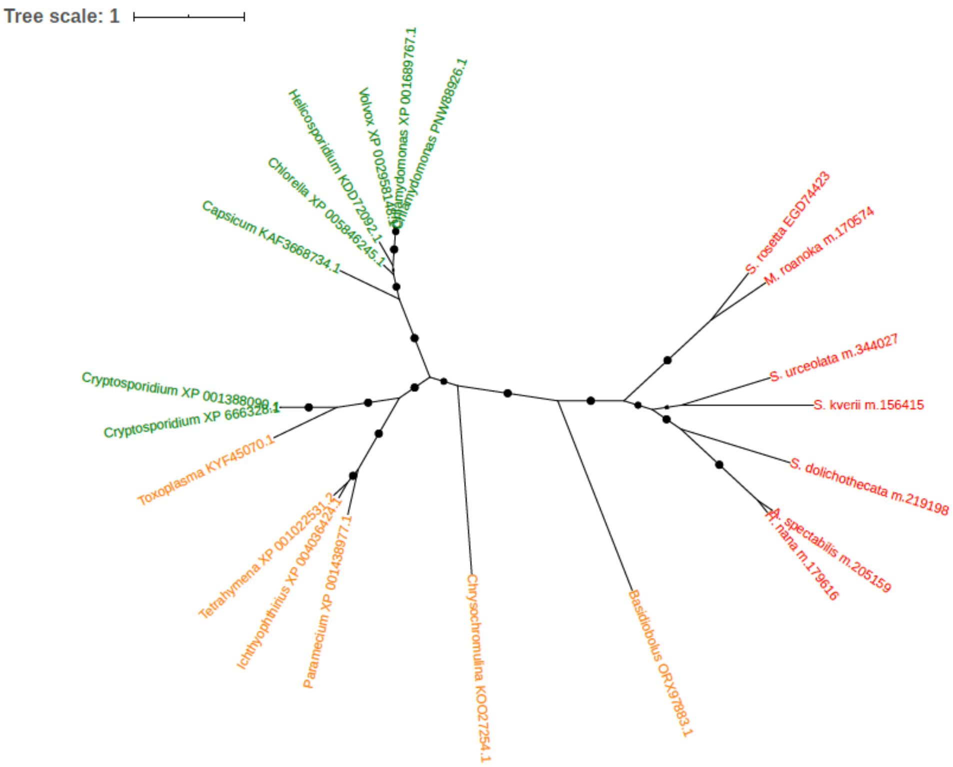

Figure S1-60. Uncharacterized protein (EGD74423)

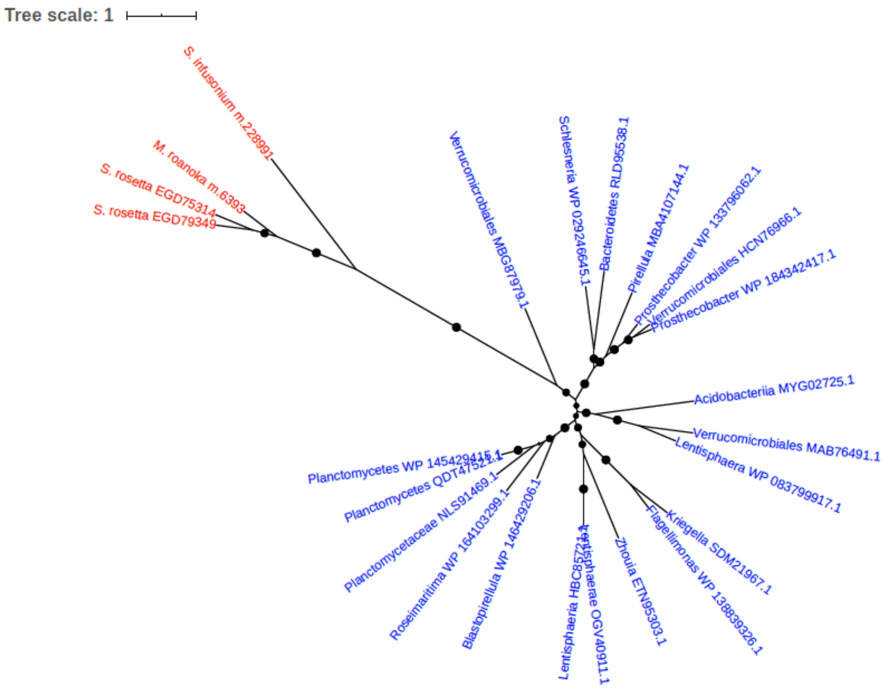

Figure S1-61. Uncharacterized protein (EGD79349)

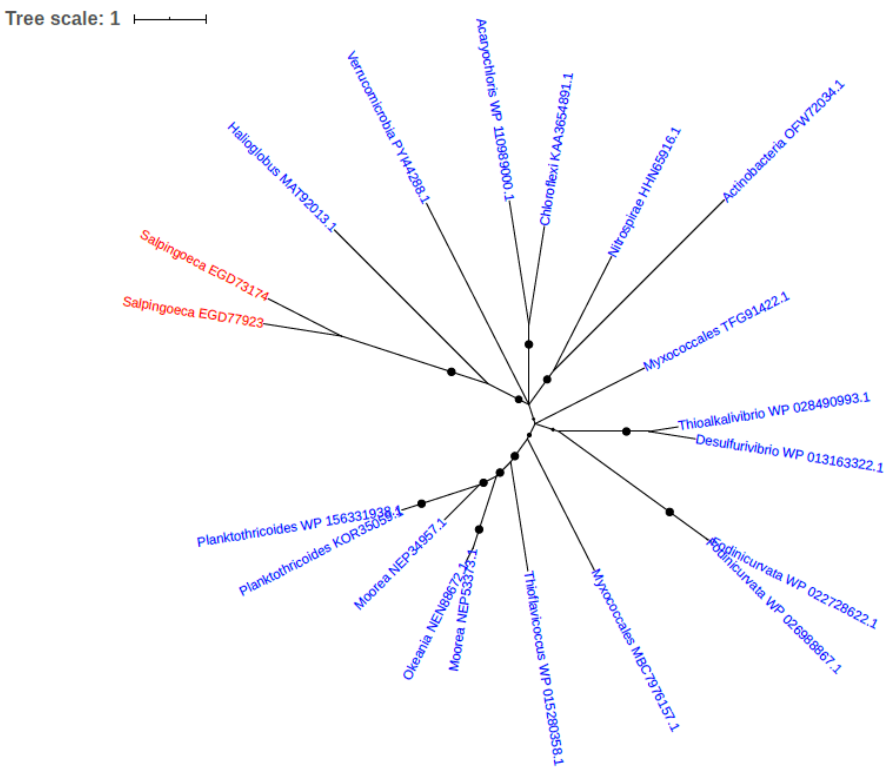

Figure S1-62. Uncharacterized protein (EGD77923 and EGD73174)

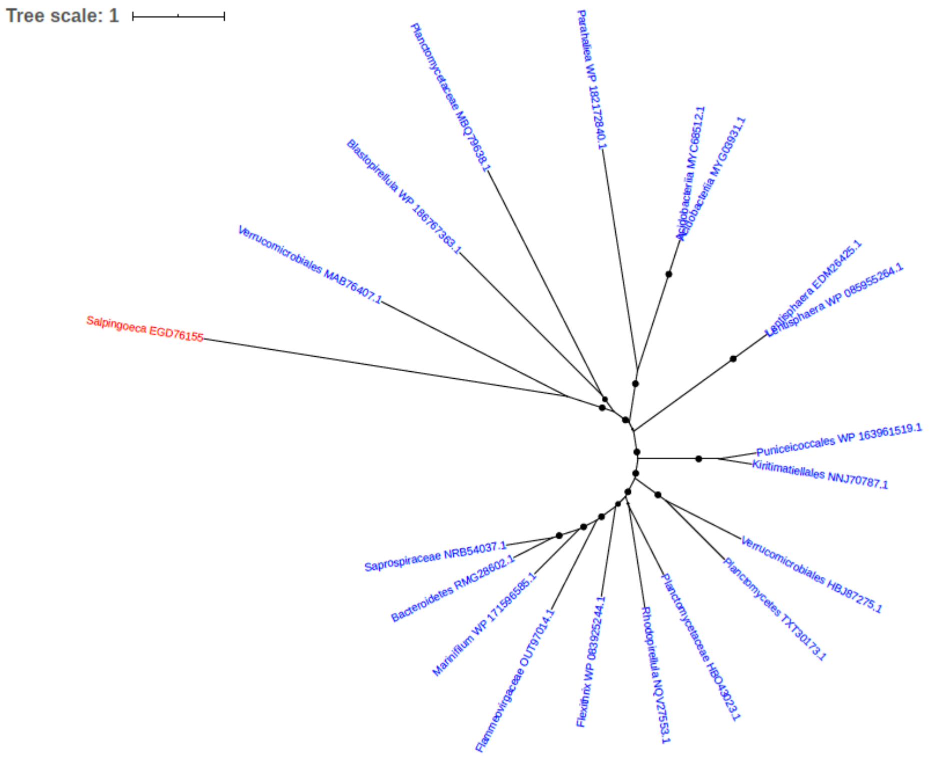

Figure S1-63. Uncharacterized protein (EGD76155)

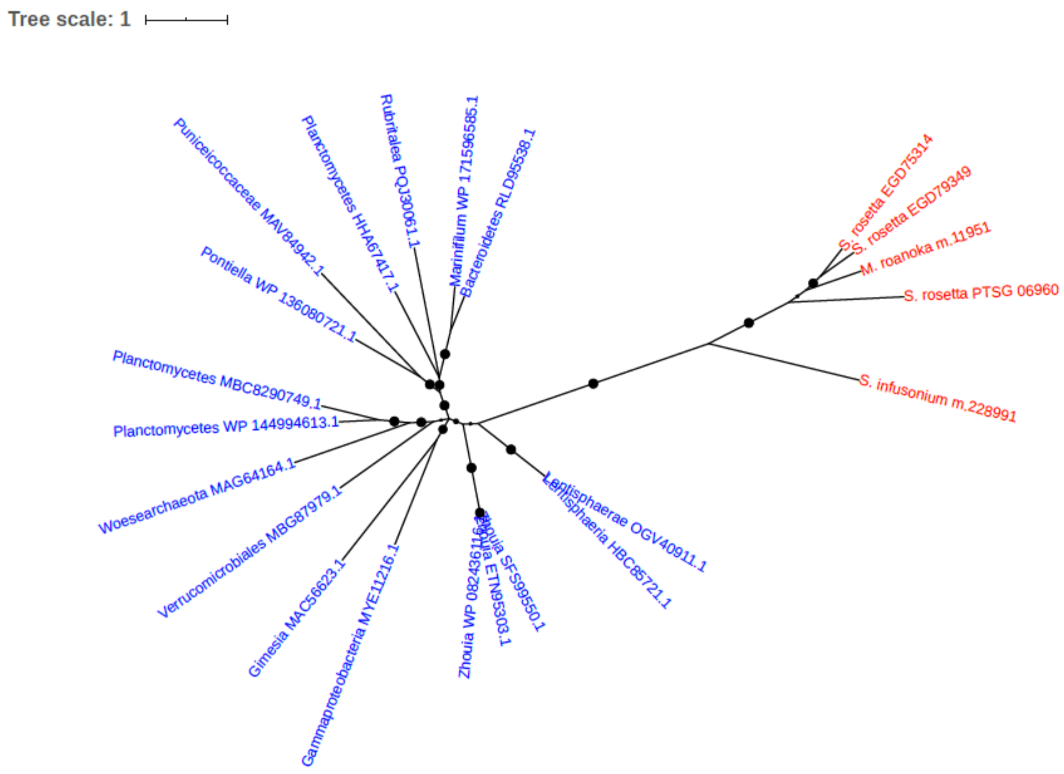

Figure S1-64. Uncharacterized protein (EGD75314)

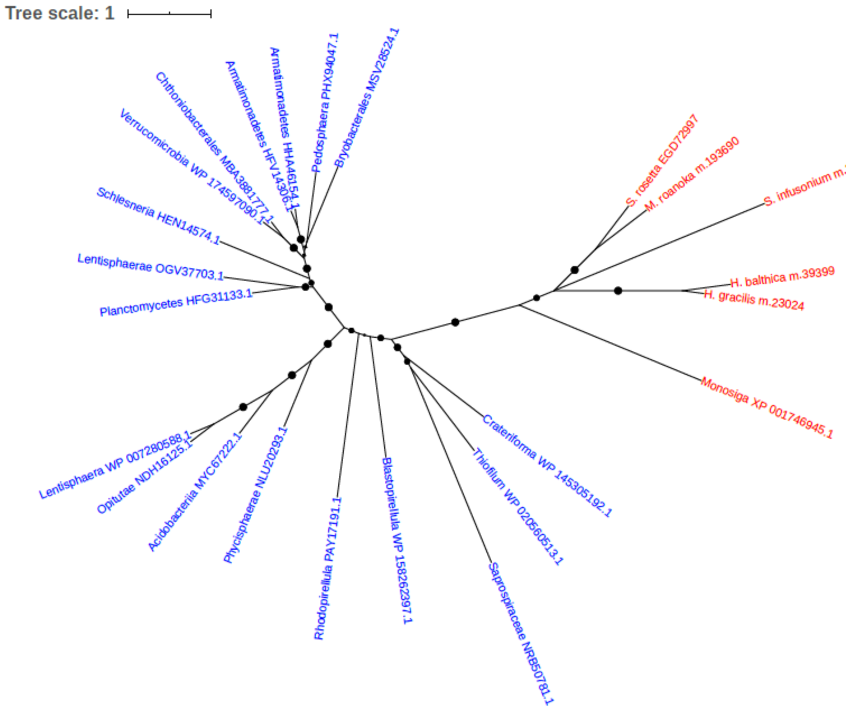

Figure S1-65. Uncharacterized protein (EGD72997)

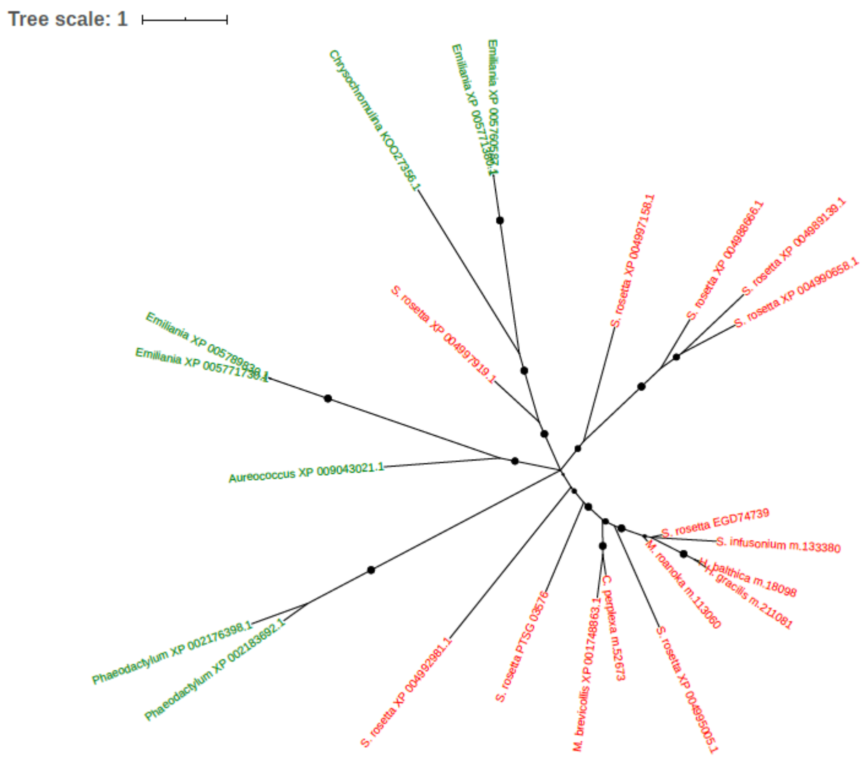

Figure S1-66. Uncharacterized protein (EGD74739)

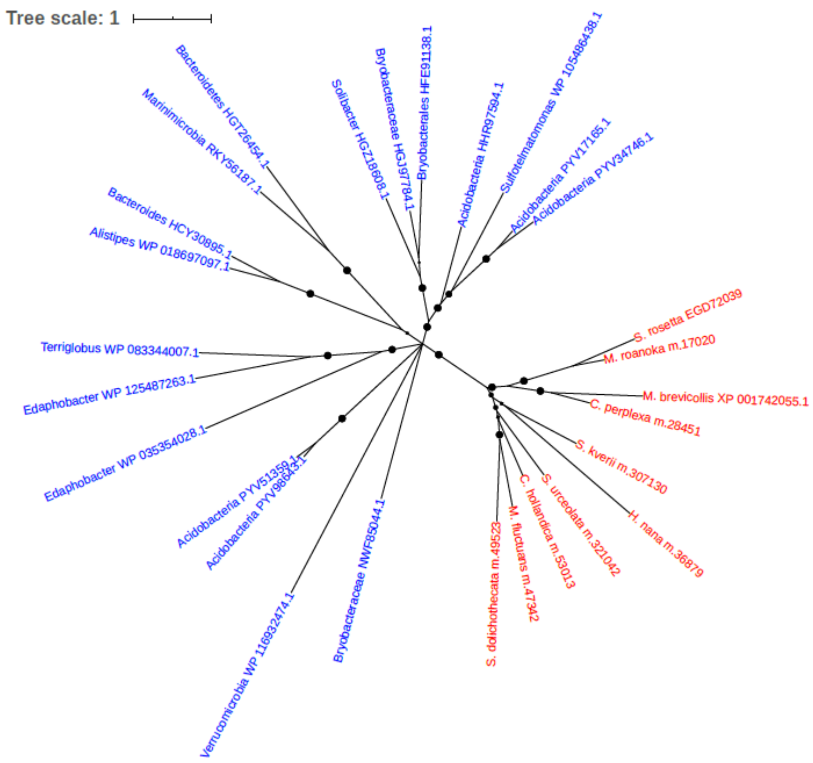

Figure S1-67. Uncharacterized protein (EGD72039)

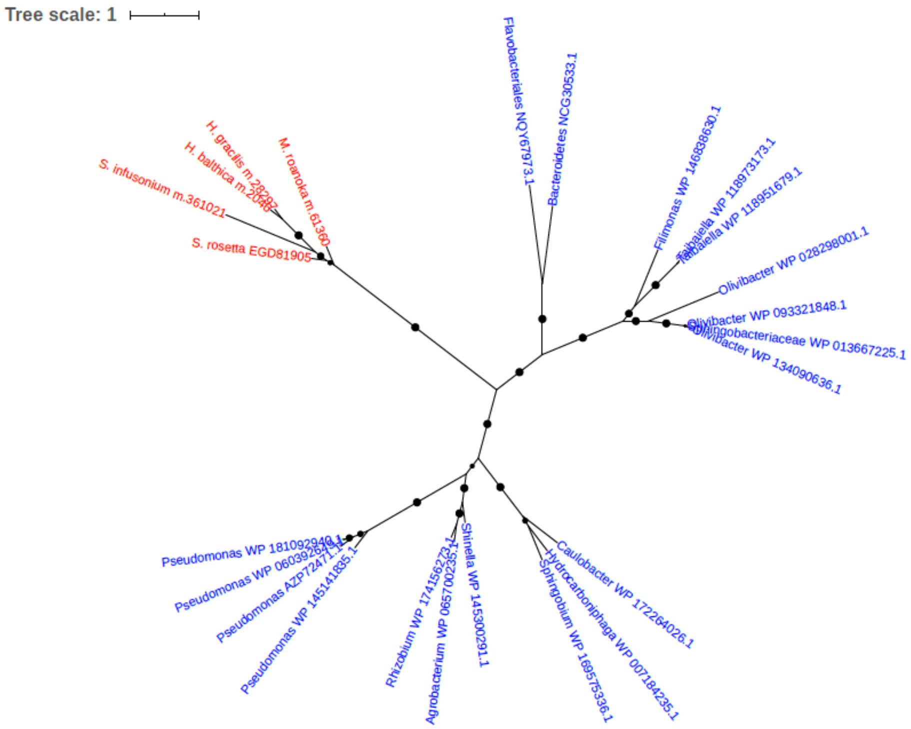

Figure S1-68. Uncharacterized protein (EGD81905)

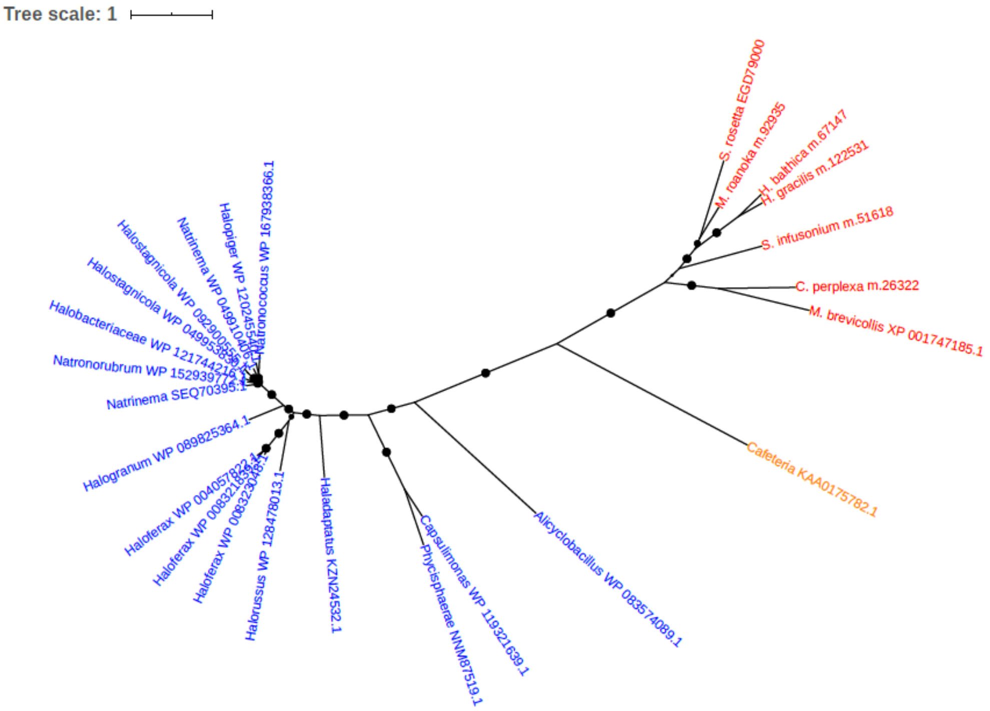

Figure S1-69. Uncharacterized protein (EGD79000)

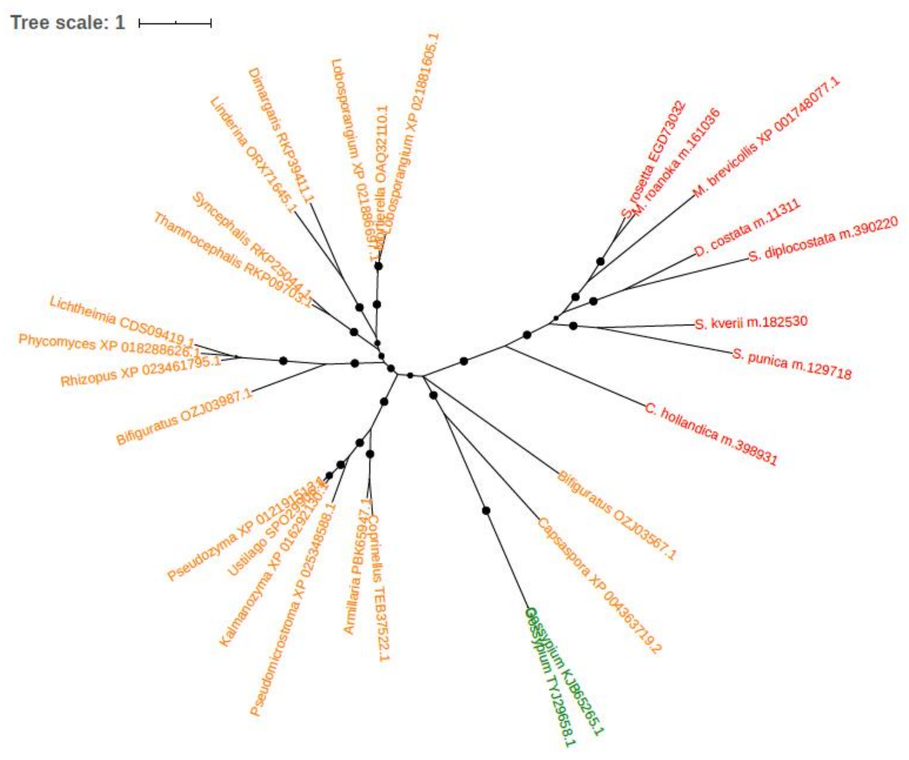

Figure S1-70. Uncharacterized protein (EGD73032)

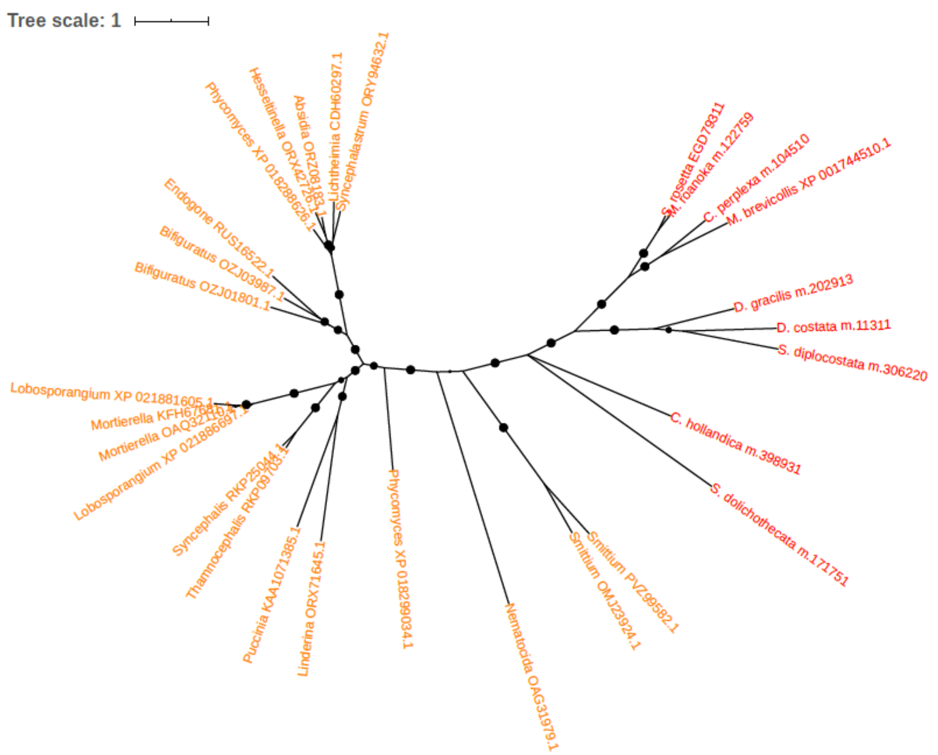

Figure S1-71. Uncharacterized protein (EGD79311)

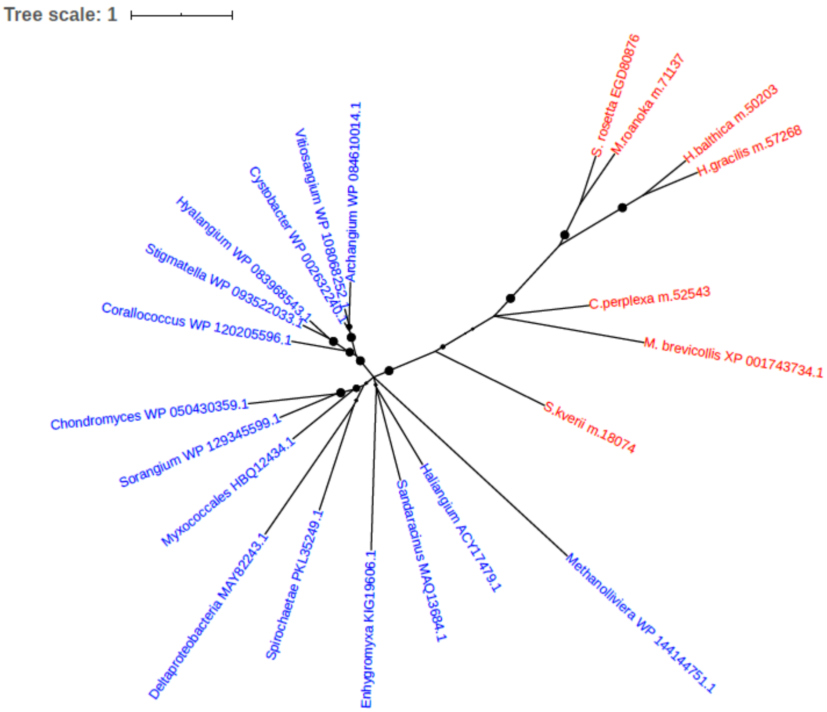

Figure S1-72. Major facilitator superfamily transporter permease (EGD80876)

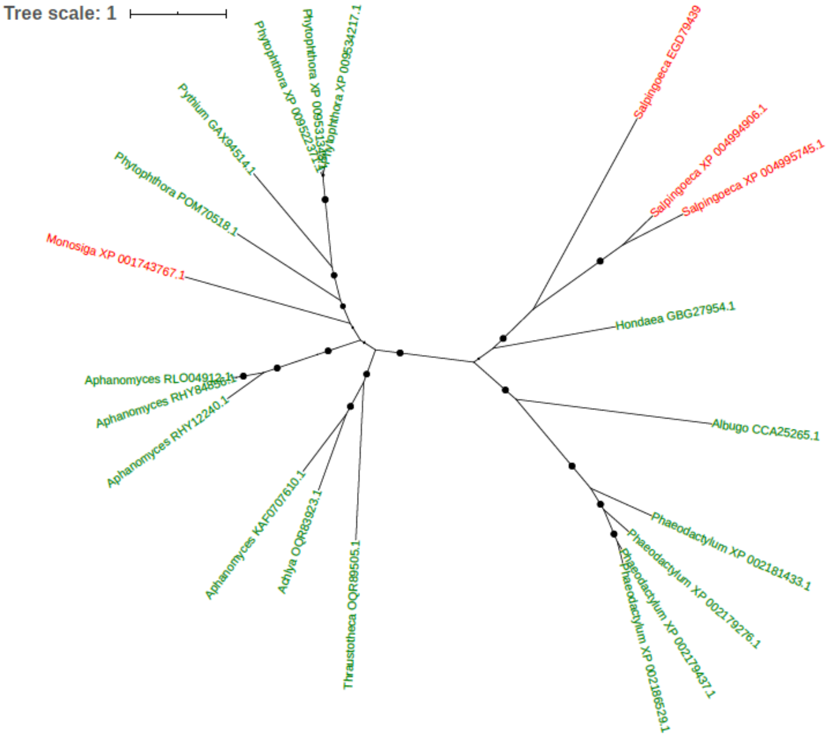

Figure S1-73. Uncharacterized protein (EGD79439)

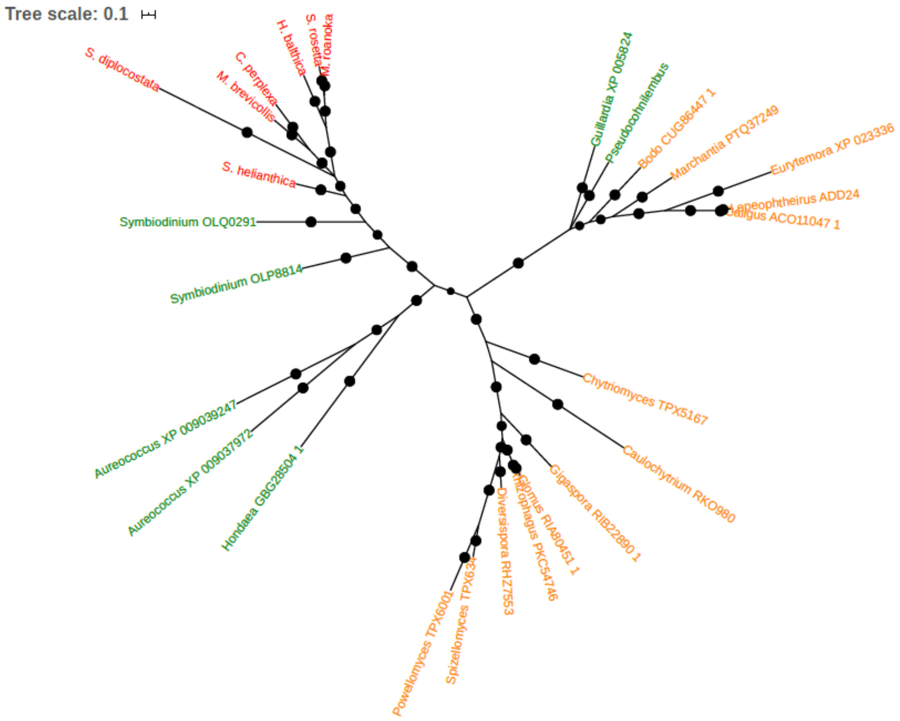

Figure S1-74. Uncharacterized protein (EGD78106)

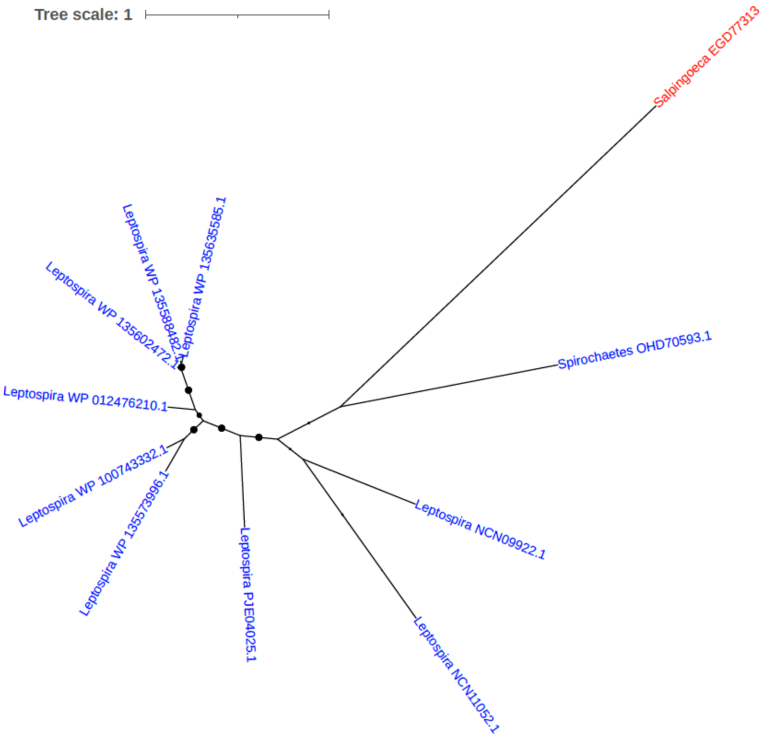

Figure S1-75. Uncharacterized protein (EGD77313)

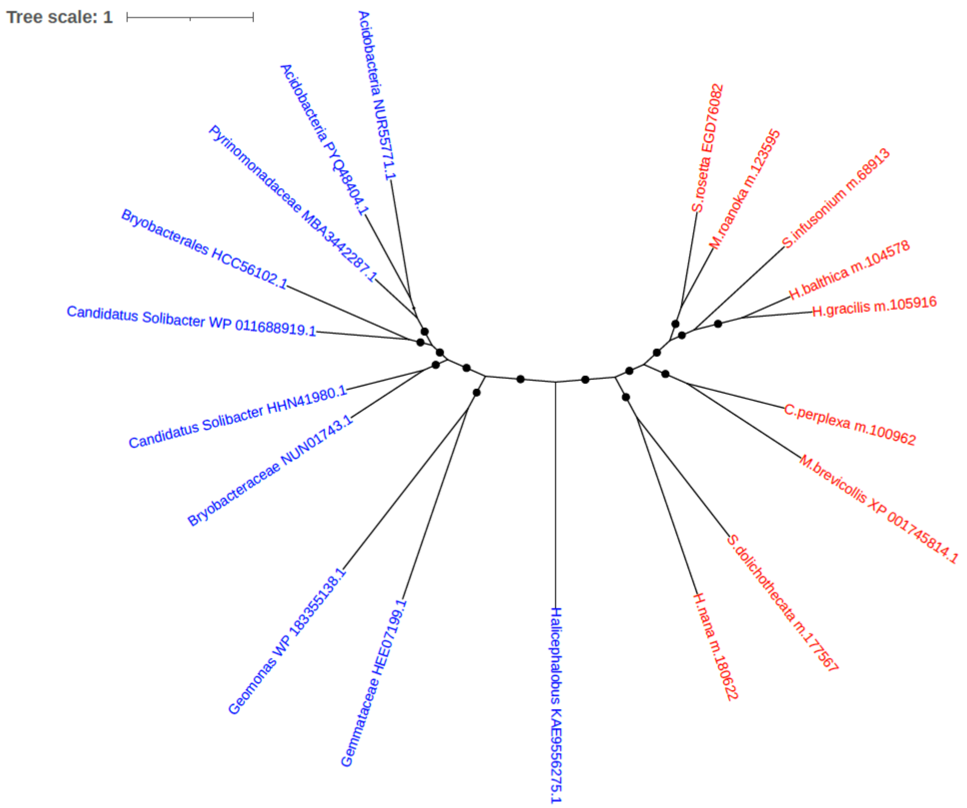

Figure S1-76. Uncharacterized protein (EGD76082)

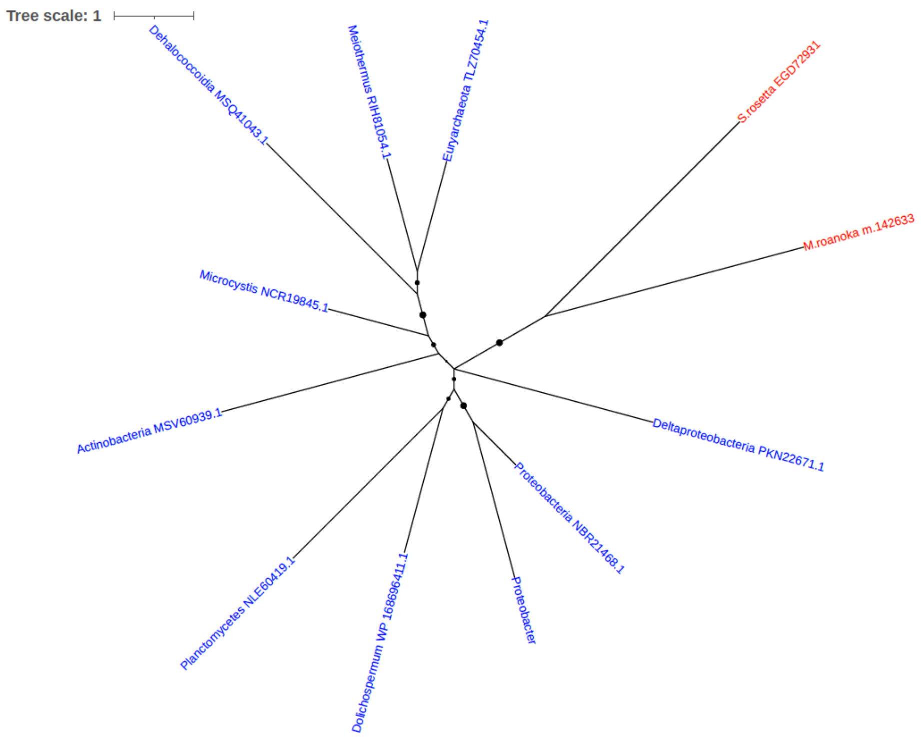

Figure S1-77. Uncharacterized protein (EGD72931)

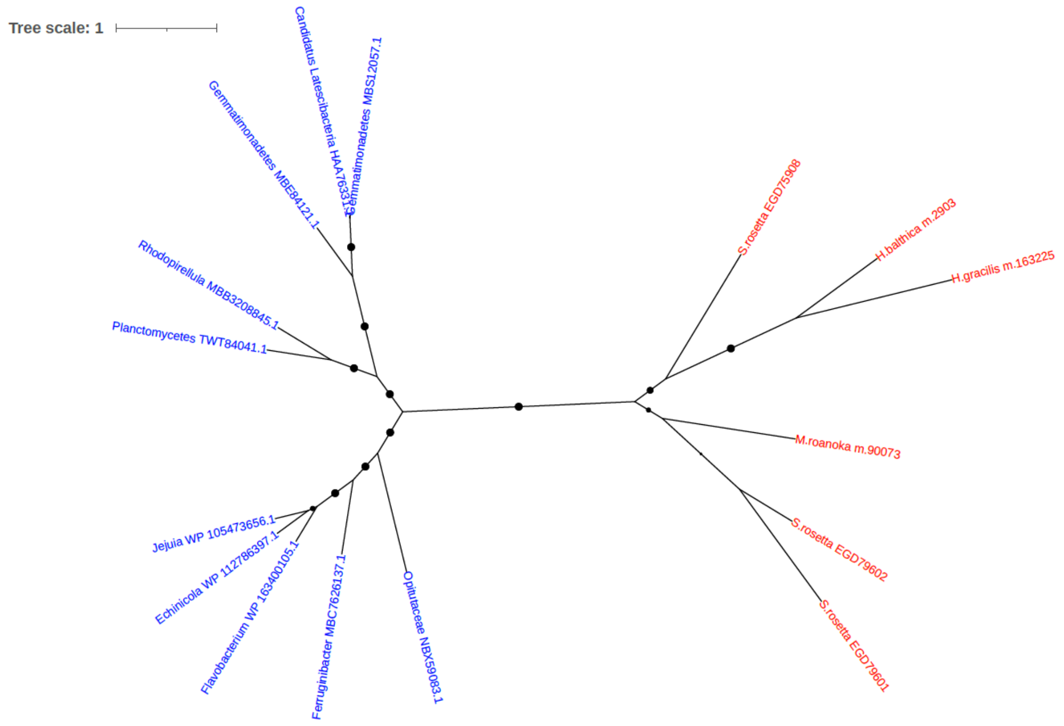

Figure S1-78. Uncharacterized protein (EGD75908)

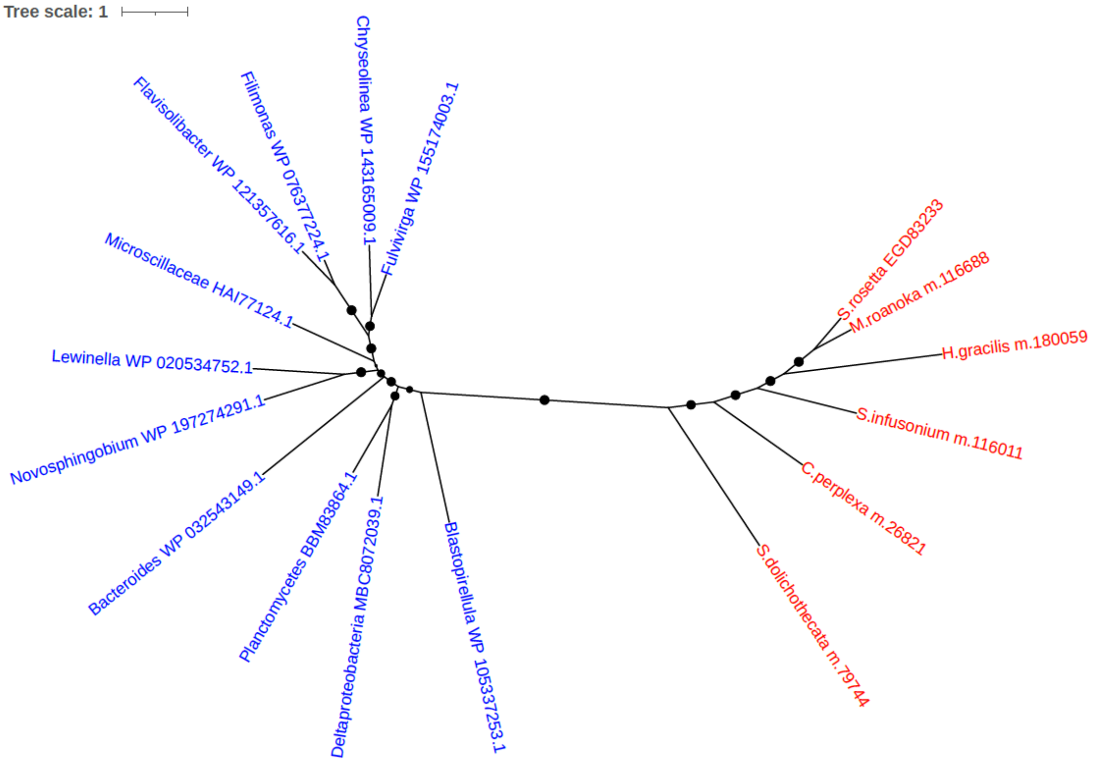

Figure S1-79. Uncharacterized protein (EGD83233)

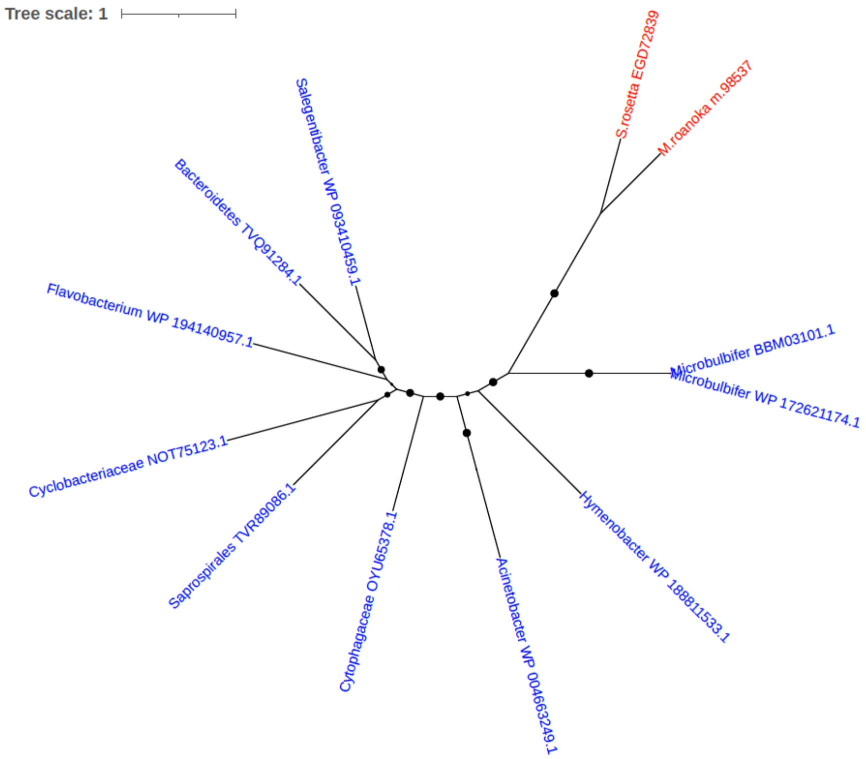

Figure S1-80. Uncharacterized protein (EGD72839)

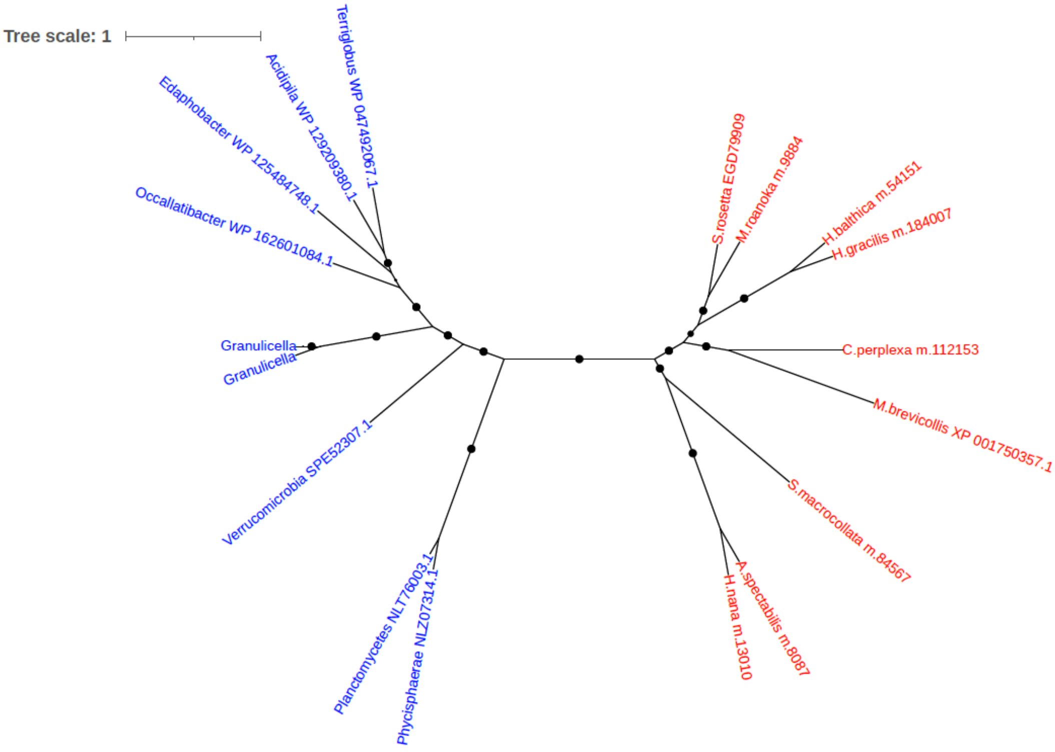

Figure S1-81. Uncharacterized protein (EGD72839)

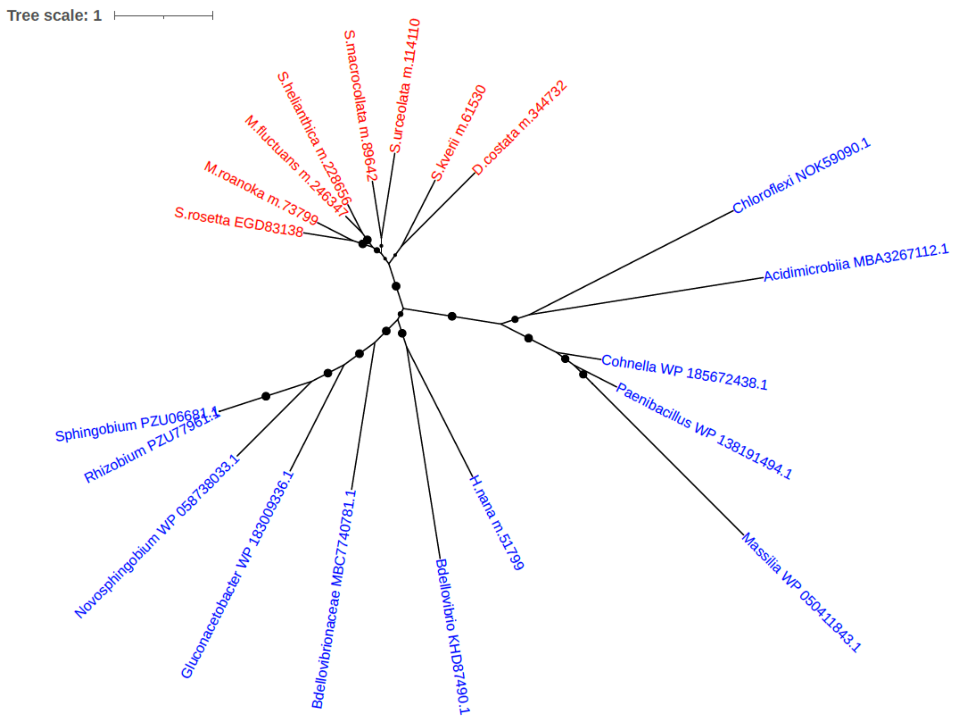

Figure S1-82. Uncharacterized protein (EGD83138)

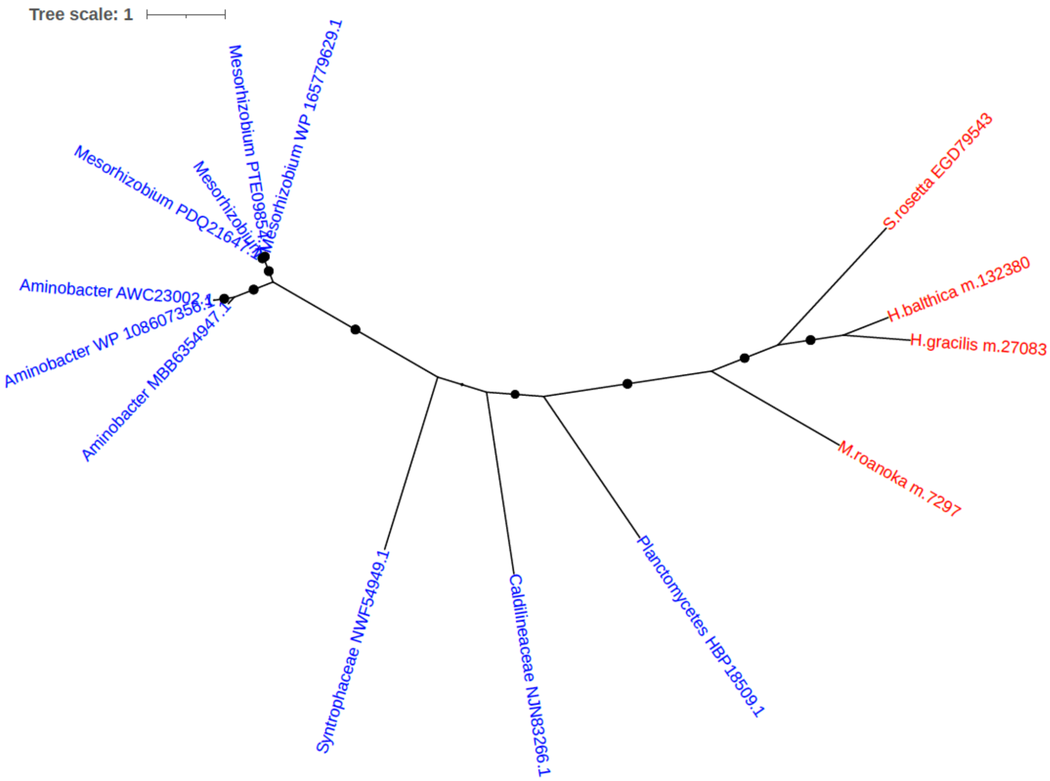

Figure S1-83. Uncharacterized protein (EGD79543)

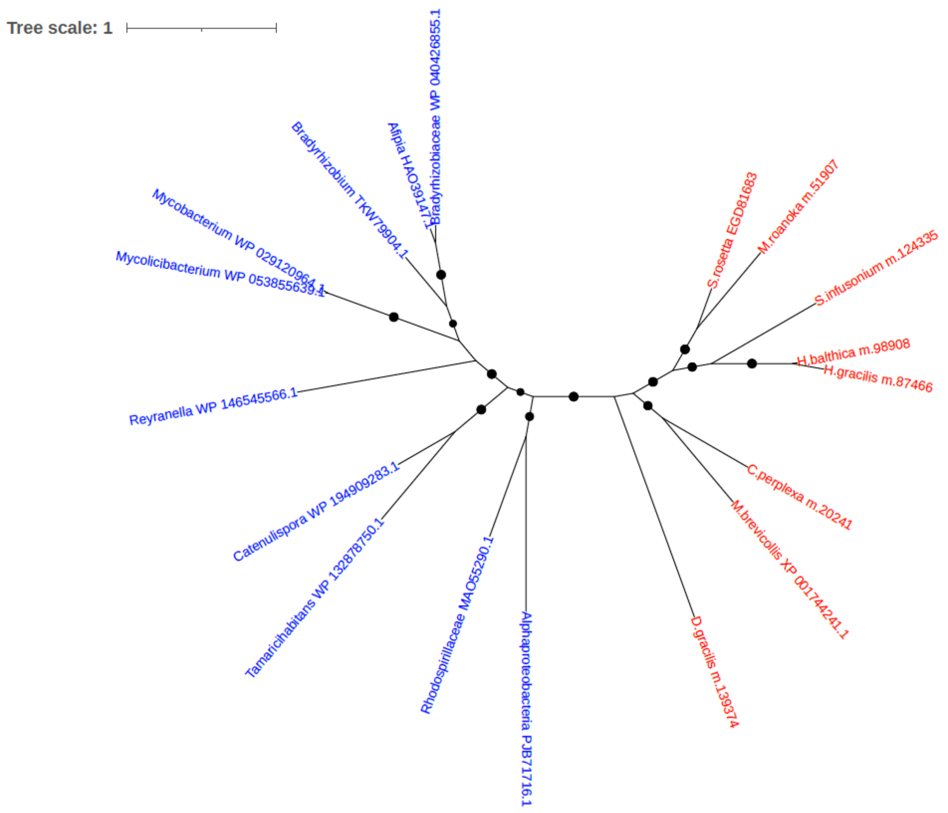

Figure S1-84. Uncharacterized protein (EGD81683)

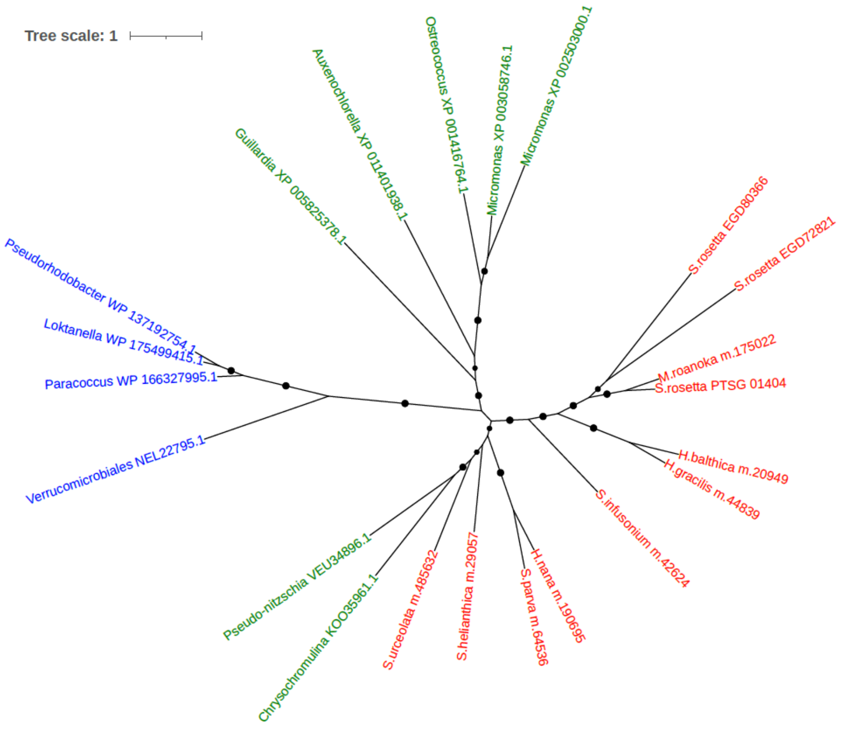

Figure S1-85. Uncharacterized protein (EGD80366)

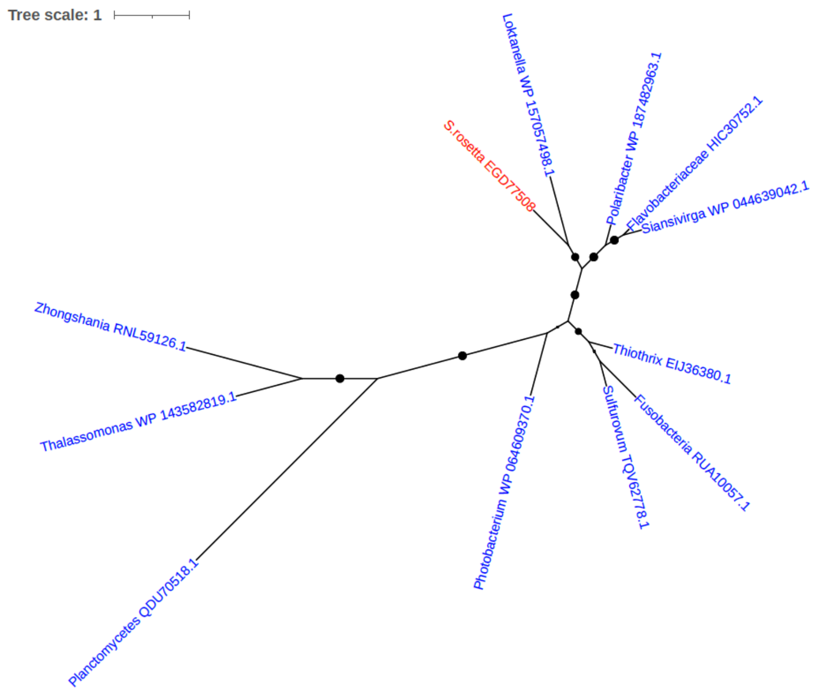

Figure S1-86. Uncharacterized protein (EGD77508)

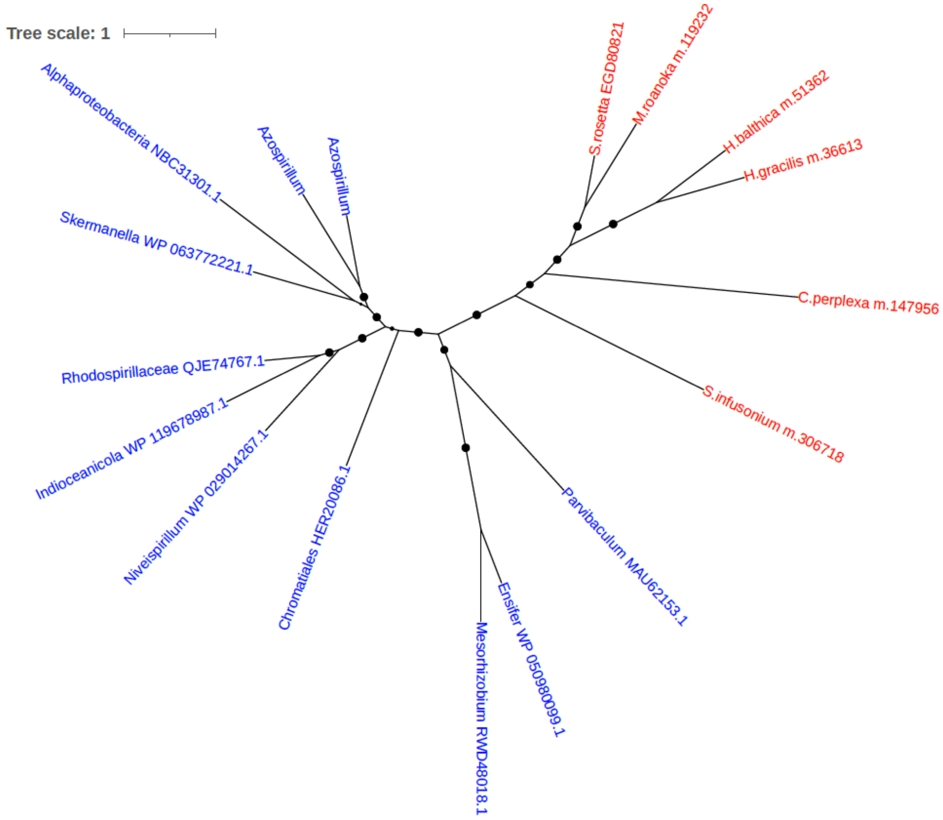

Figure S1-87. Uncharacterized protein (EGD80821)

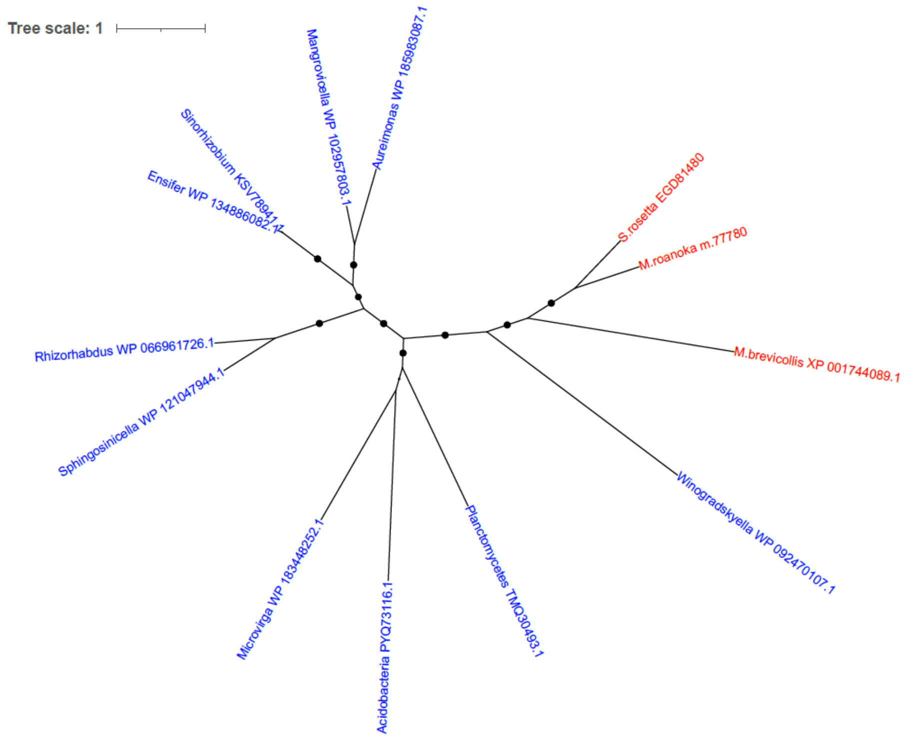

Figure S1-88. Uncharacterized protein (EGD81480)

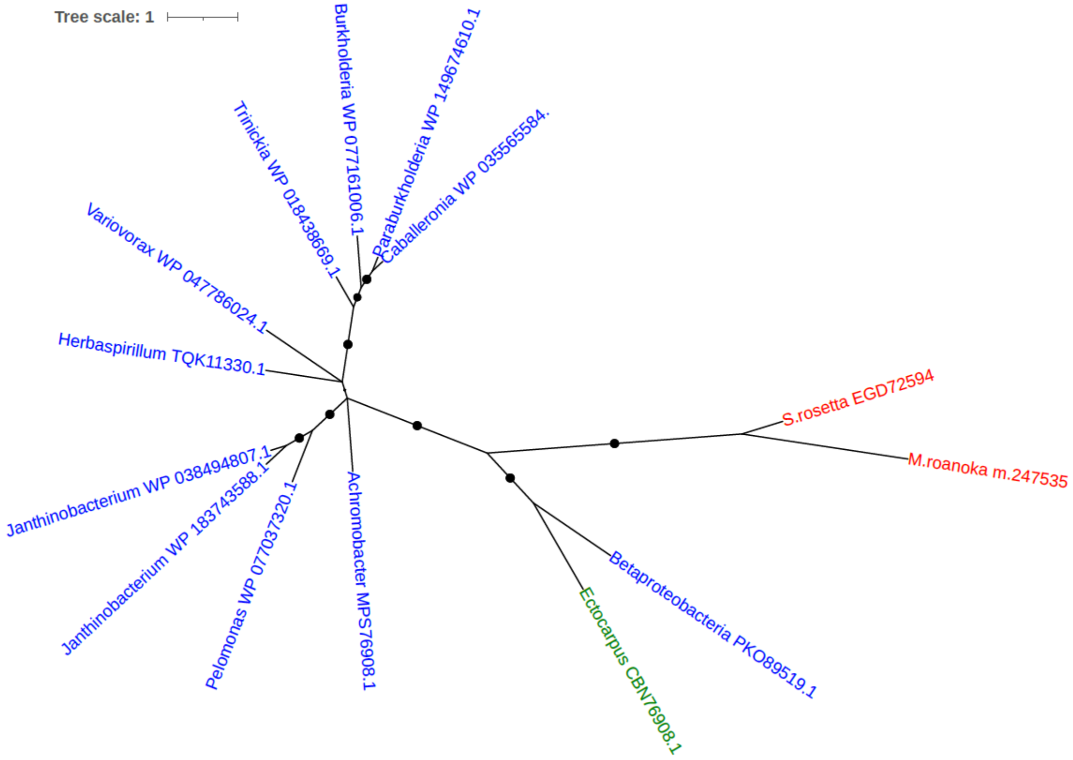

Figure S1-89. Uncharacterized protein (EGD72594)

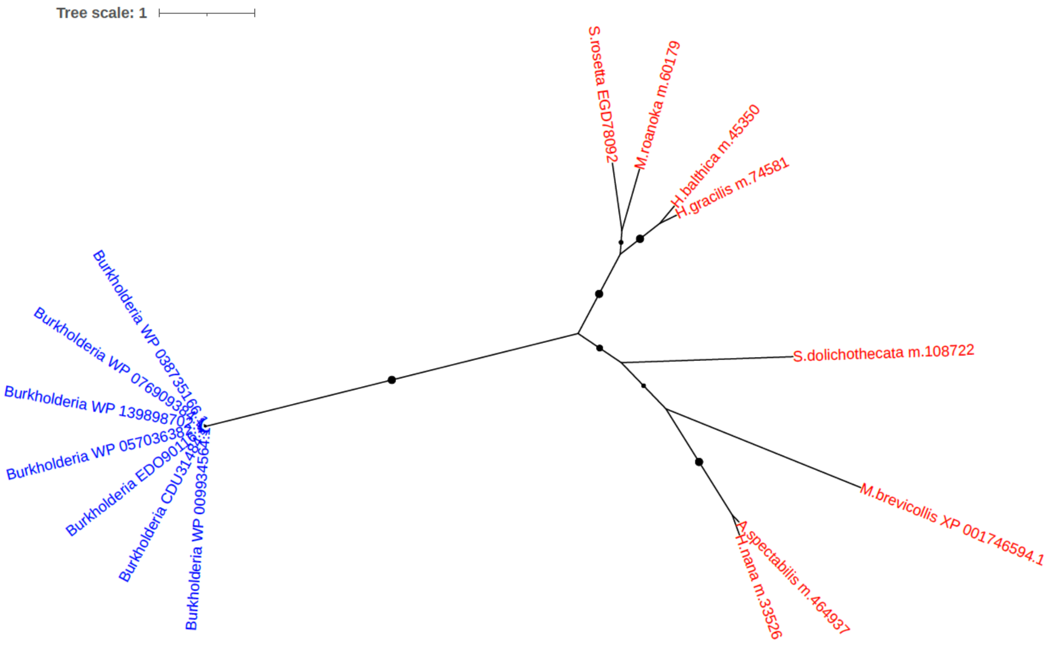

Figure S1-90. Uncharacterized protein (EGD78092)

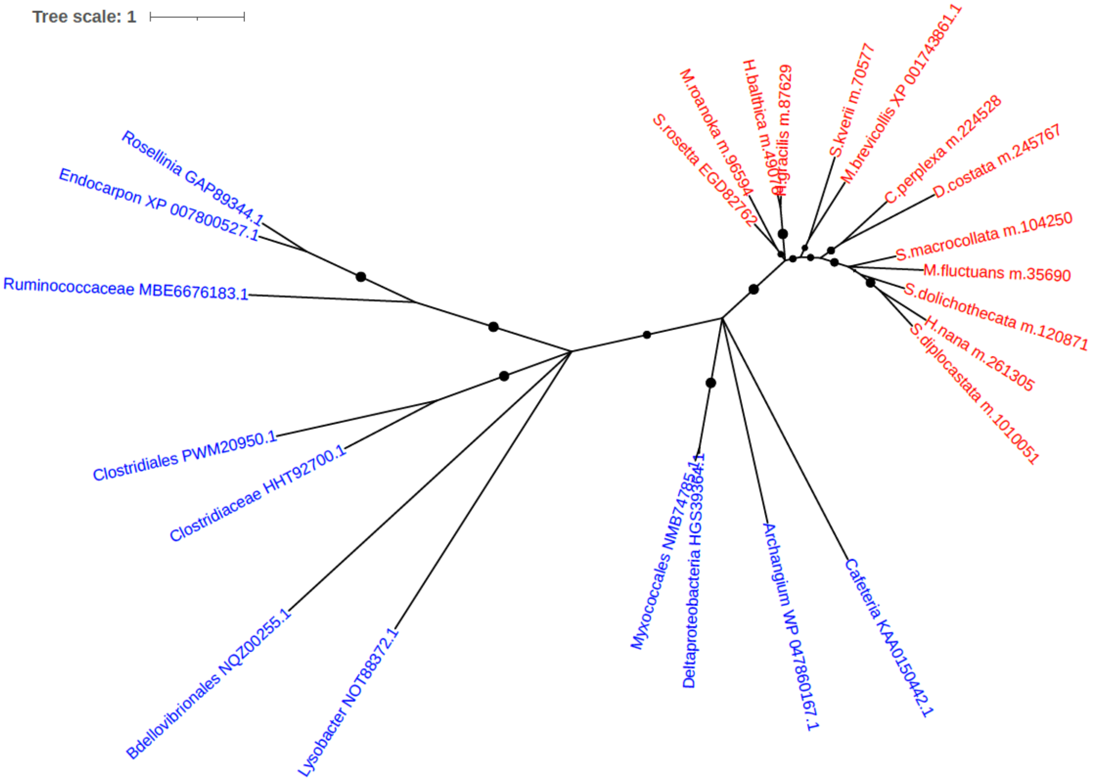

Figure S1-91. Uncharacterized protein (EGD82762)

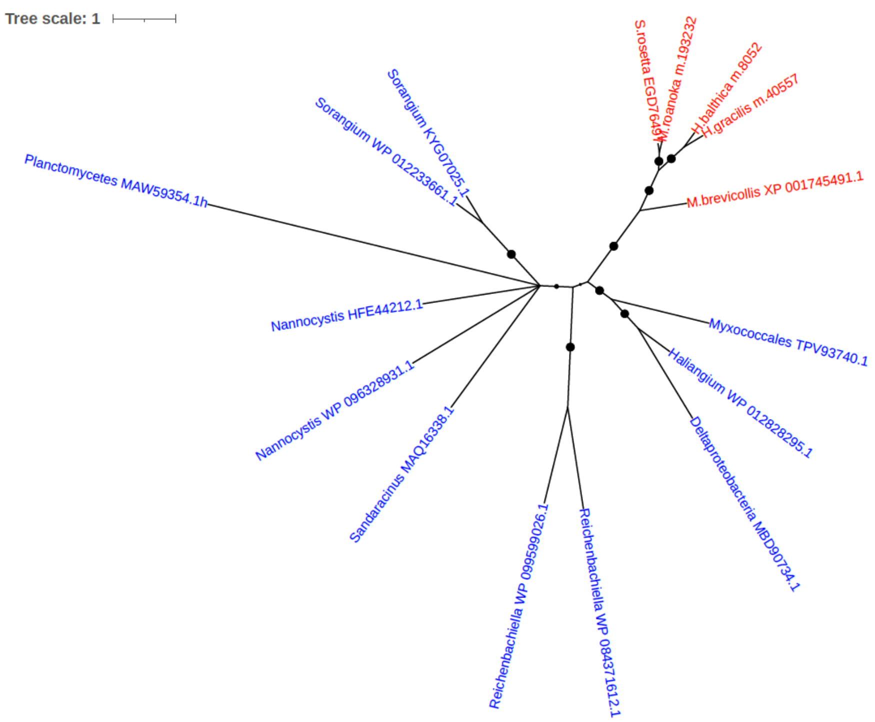

Figure S1-92. Uncharacterized protein (EGD76497)

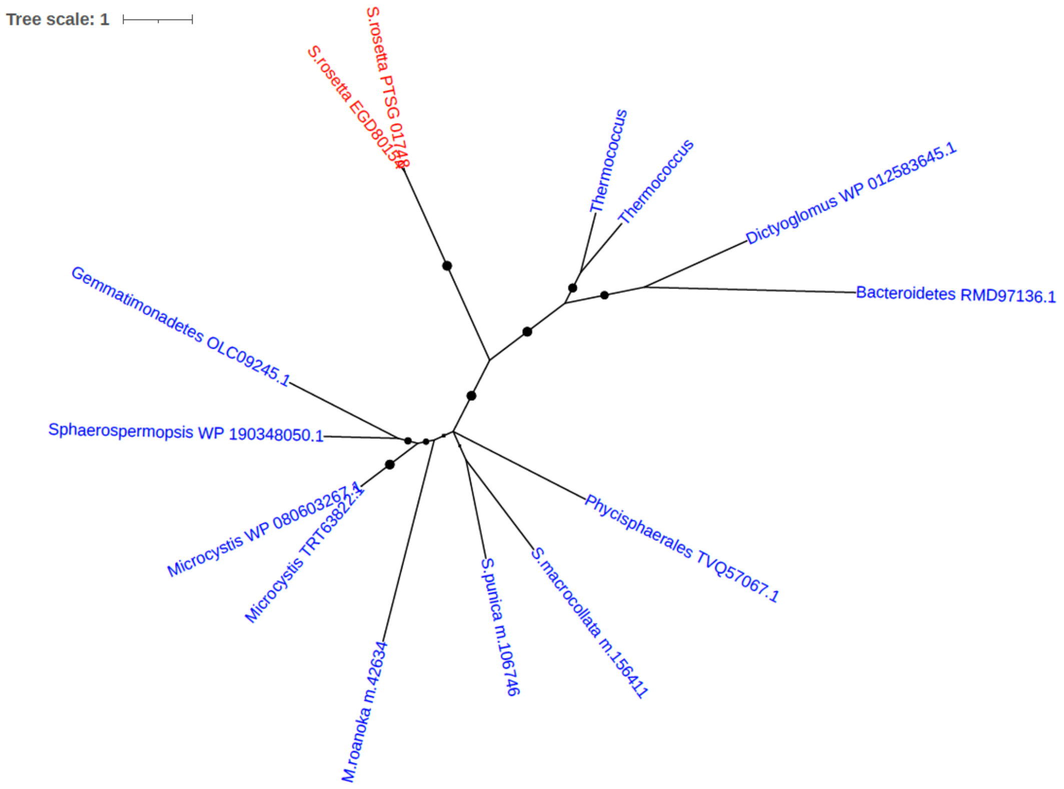

Figure S1-93. Uncharacterized protein (EGD80154)

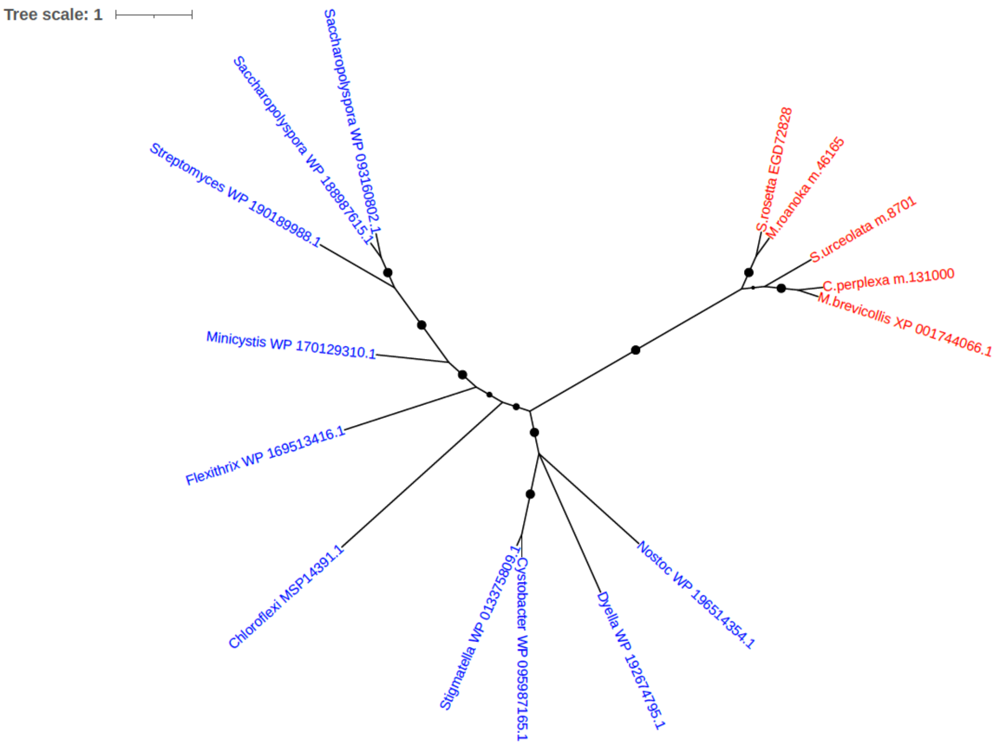

Figure S1-94. Uncharacterized protein (EGD72828)

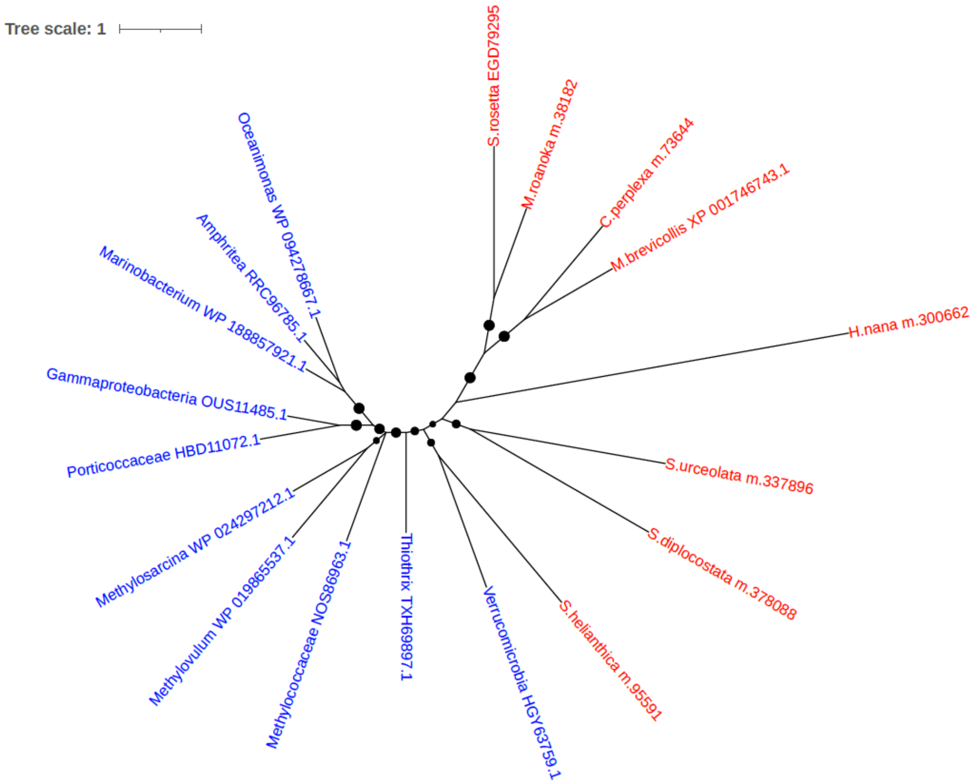

Figure S1-95. Uncharacterized protein (EGD79295)

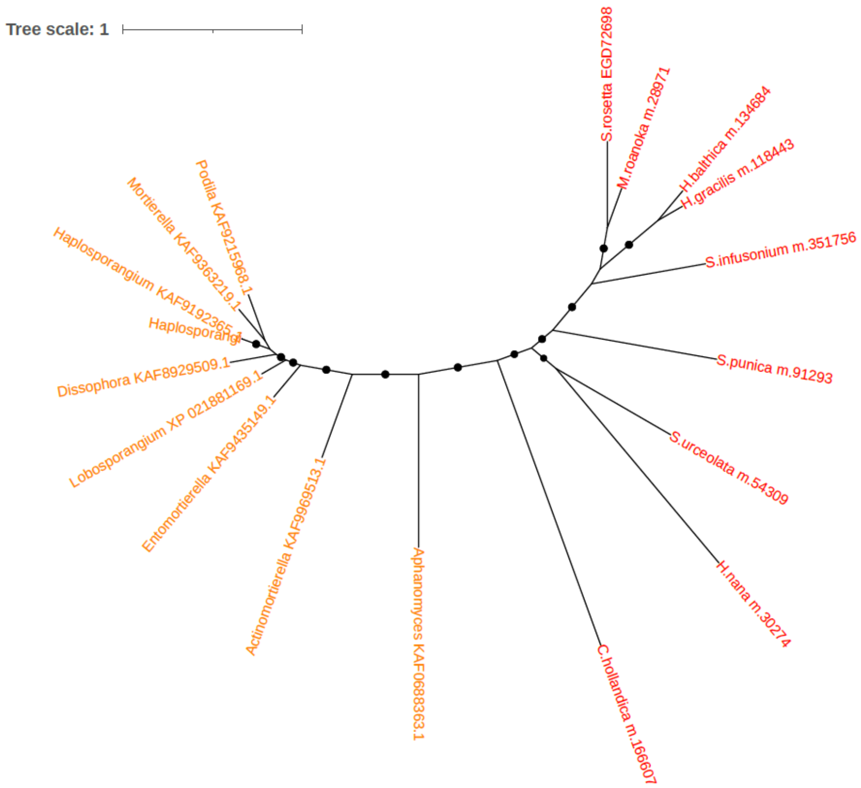

Figure S1-96. Uncharacterized protein (EGD72698)

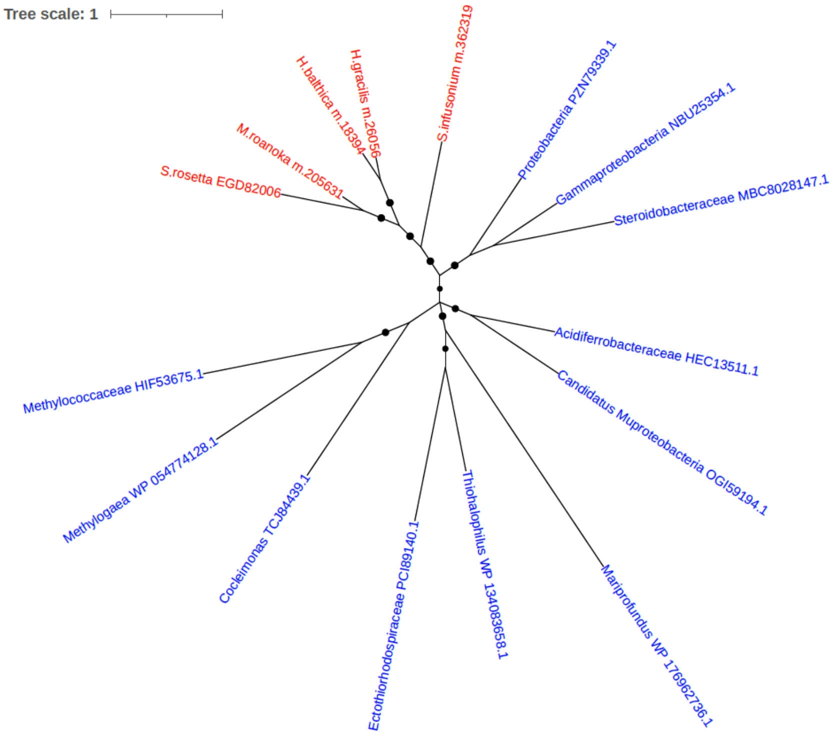

Figure S1-97. Uncharacterized protein (EGD82006)

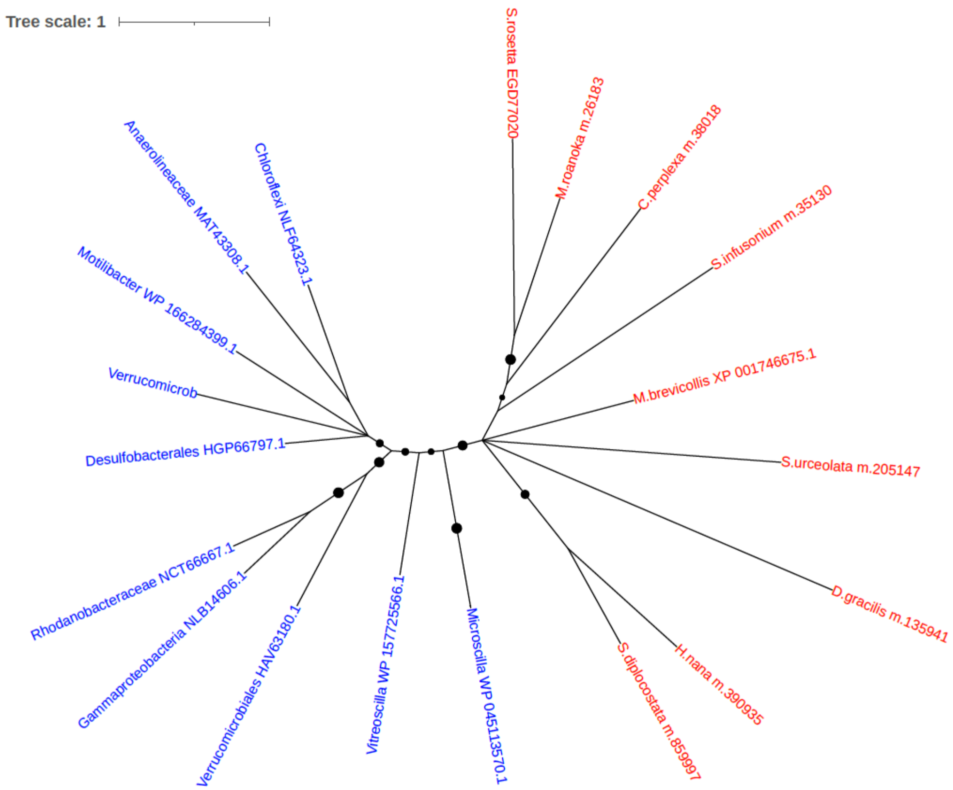

Figure S1-98. Uncharacterized protein (EGD77020)

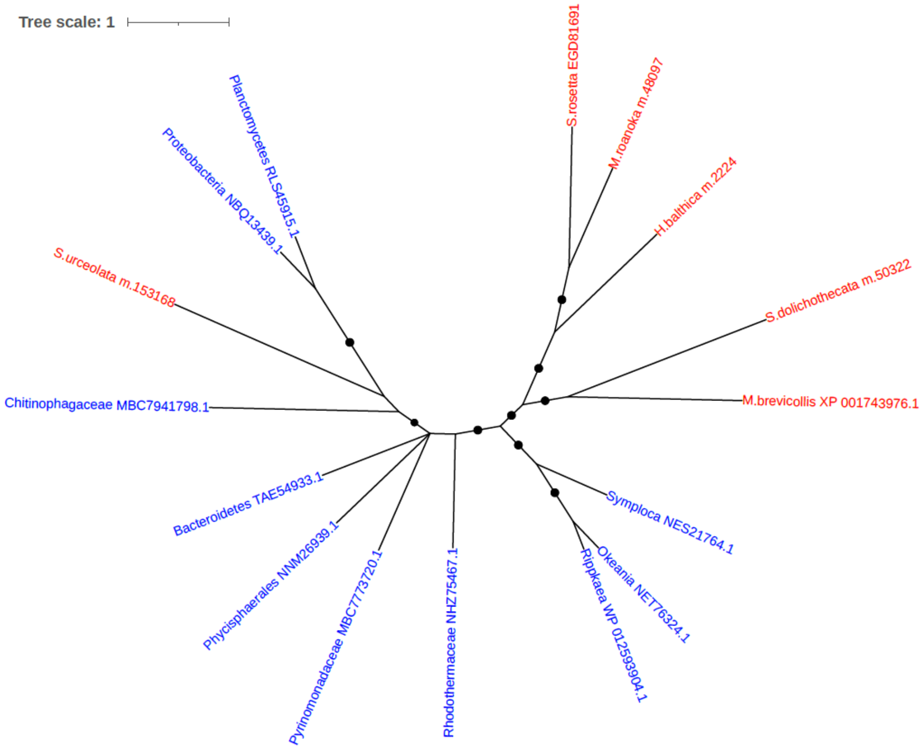

Figure S1-99. Uncharacterized protein (EGD81691)

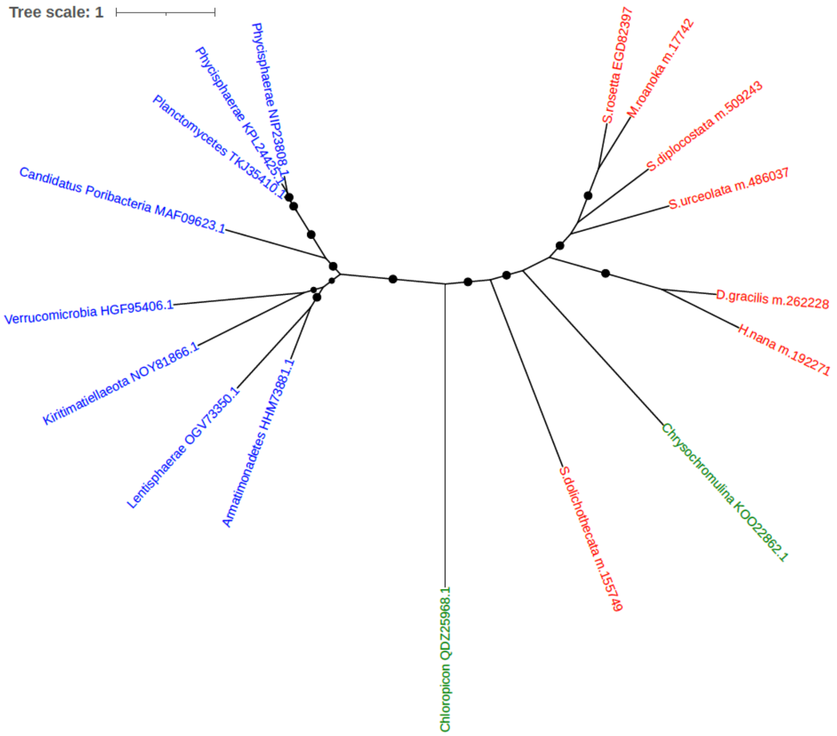

Figure S1-100. Uncharacterized protein (EGD82397)

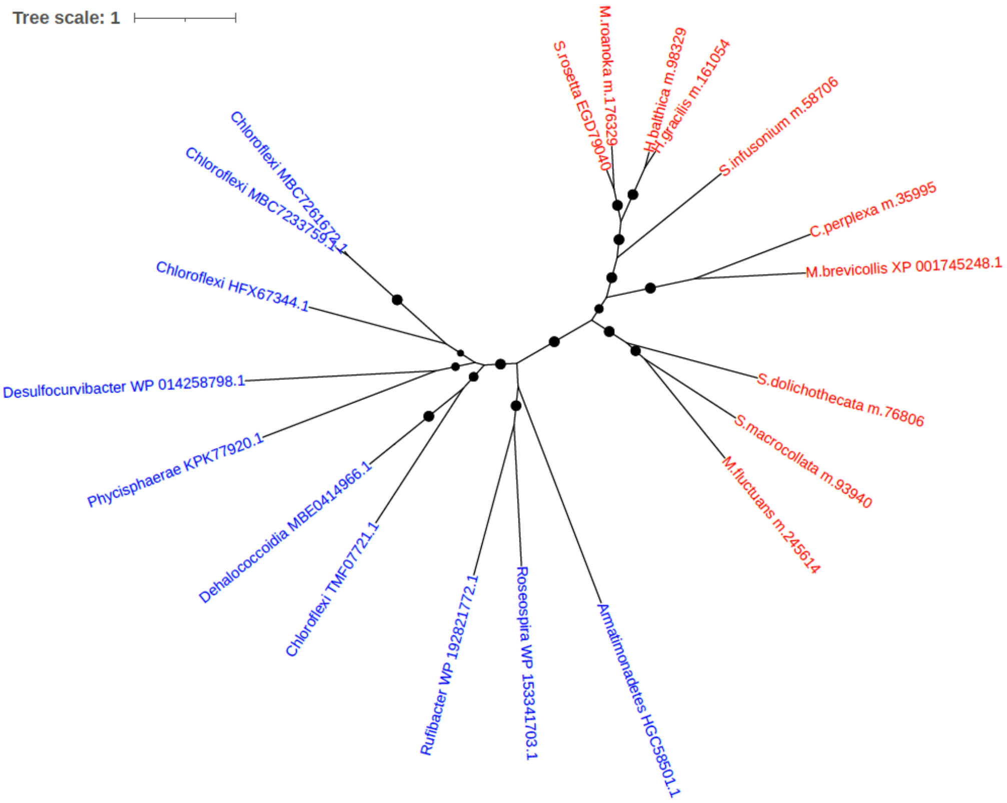

Figure S1-101. Uncharacterized protein (EGD79040)

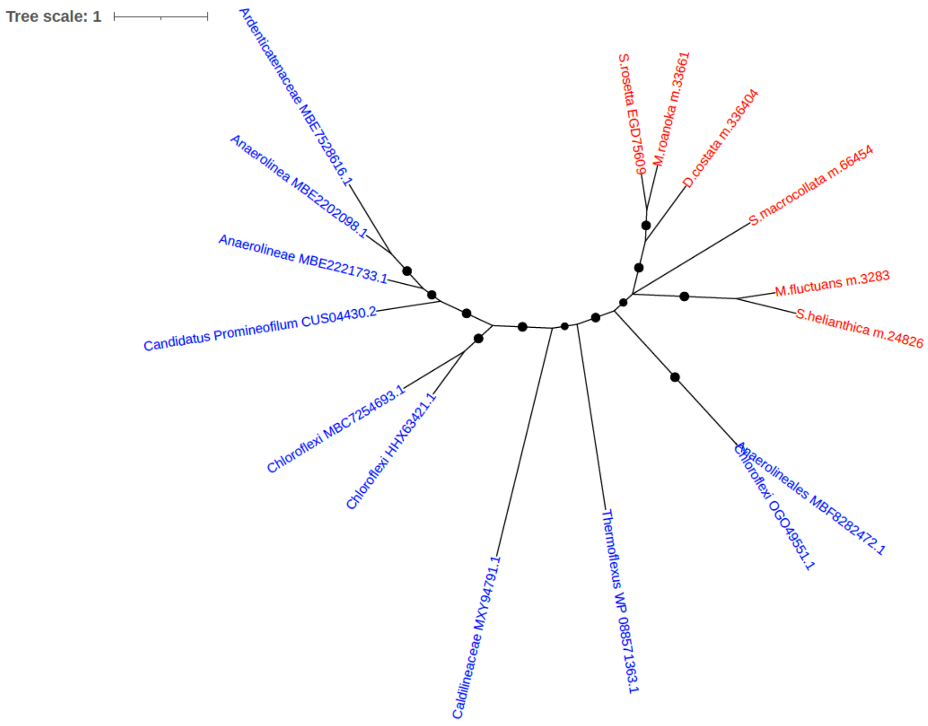

Figure S1-102. Uncharacterized protein (EGD75609)

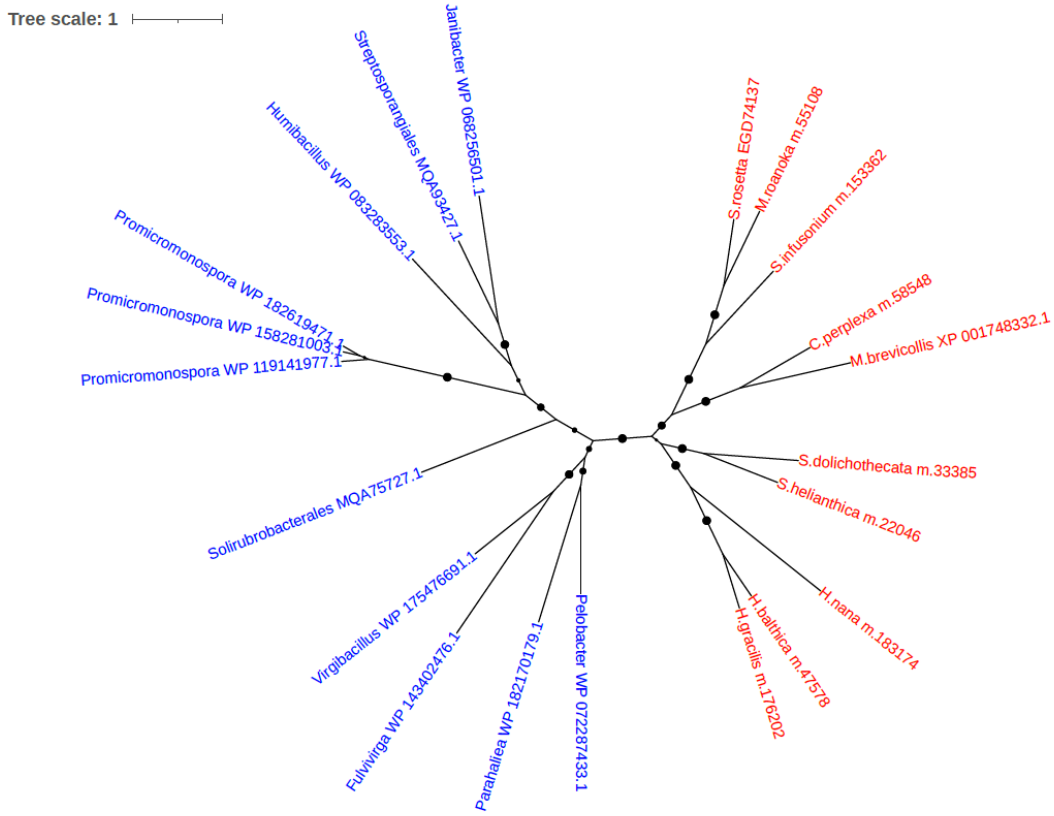

Figure S1-103. Uncharacterized protein (EGD74137)

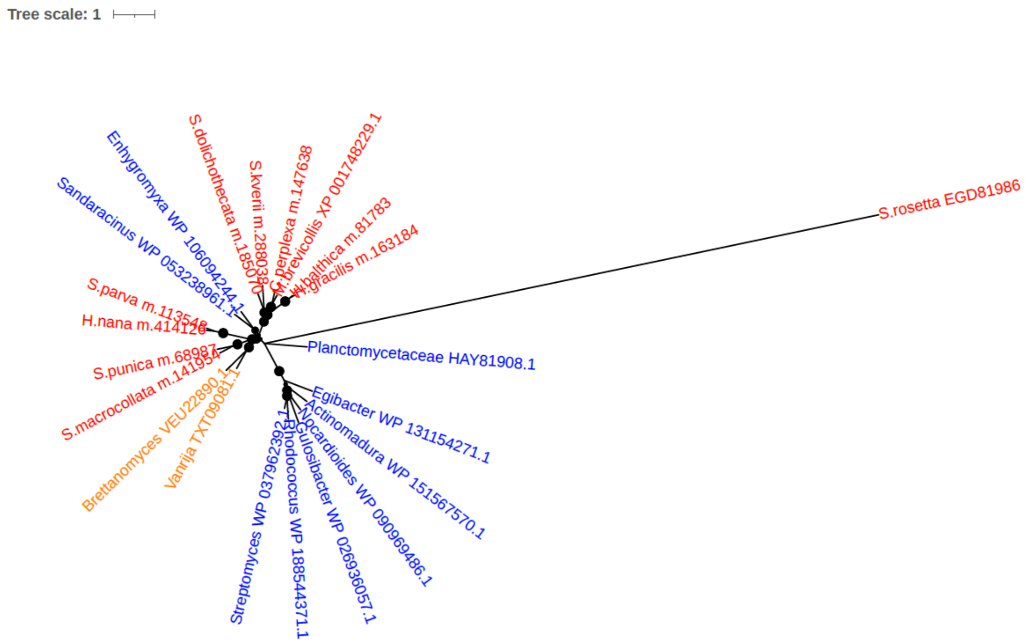

Figure S1-104. Uncharacterized protein (EGD81986)

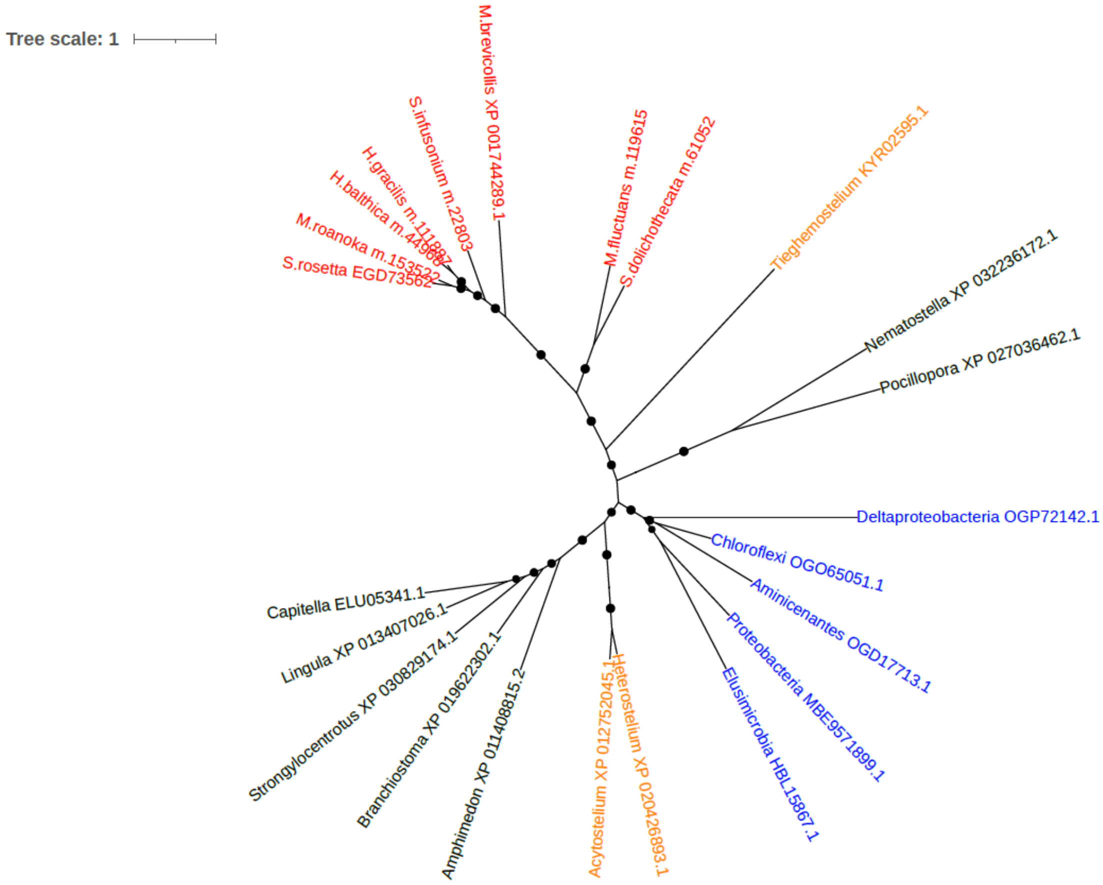

Figure S1-105. Uncharacterized protein (EGD73562)

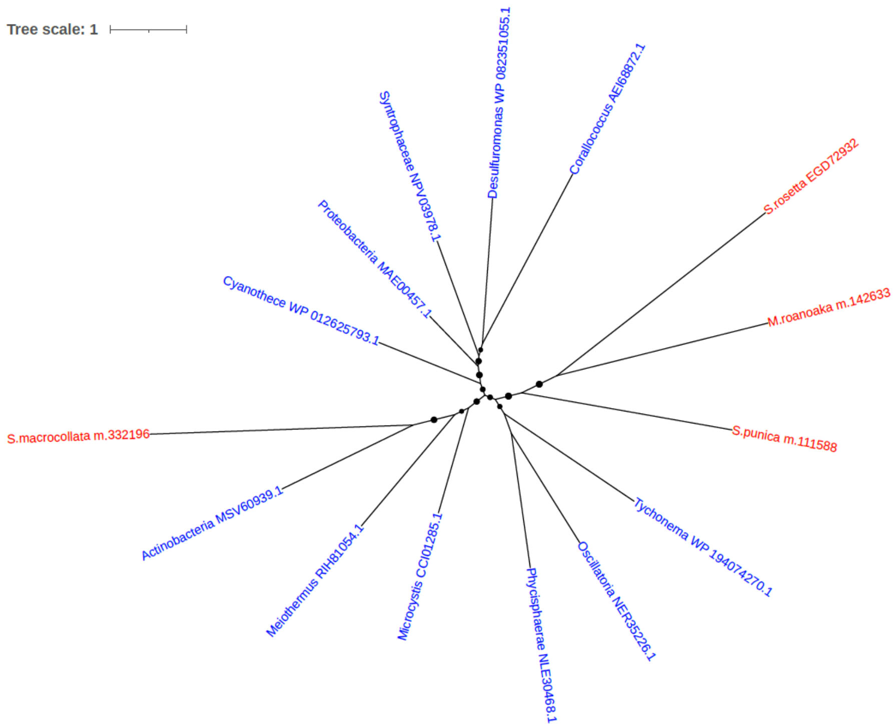

Figure S1-106. Uncharacterized protein (EGD73562)

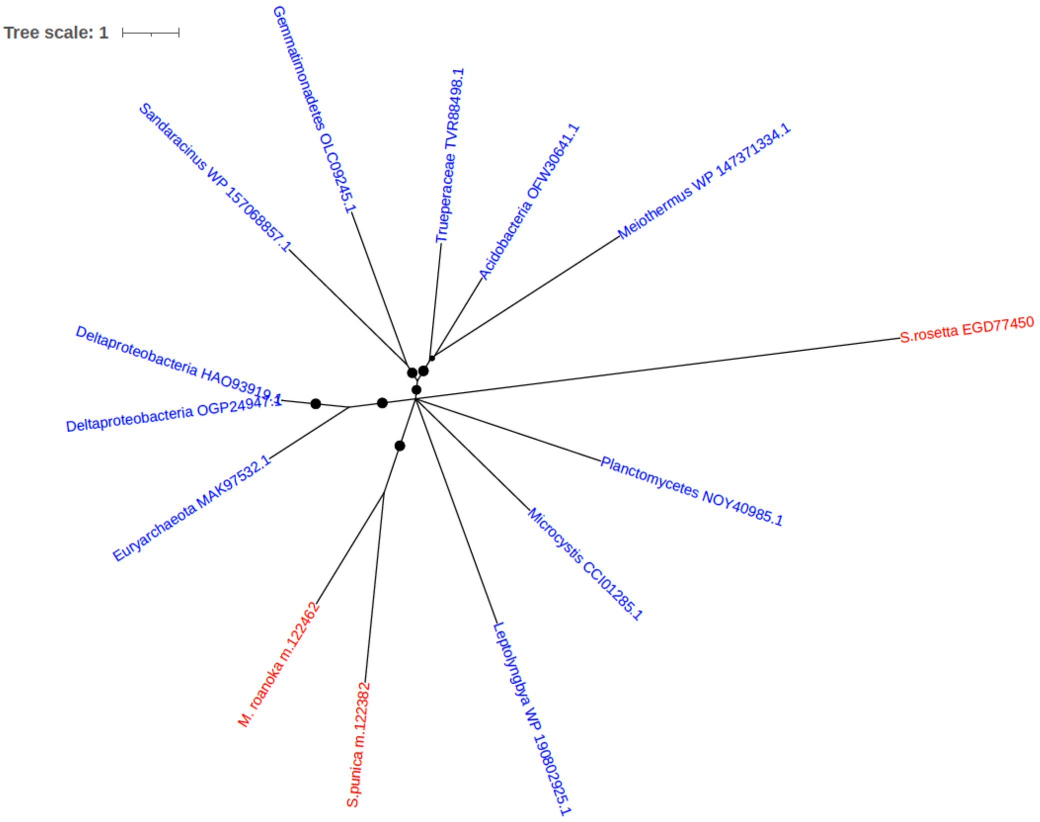

Figure S1-107. Uncharacterized protein (EGD77450)

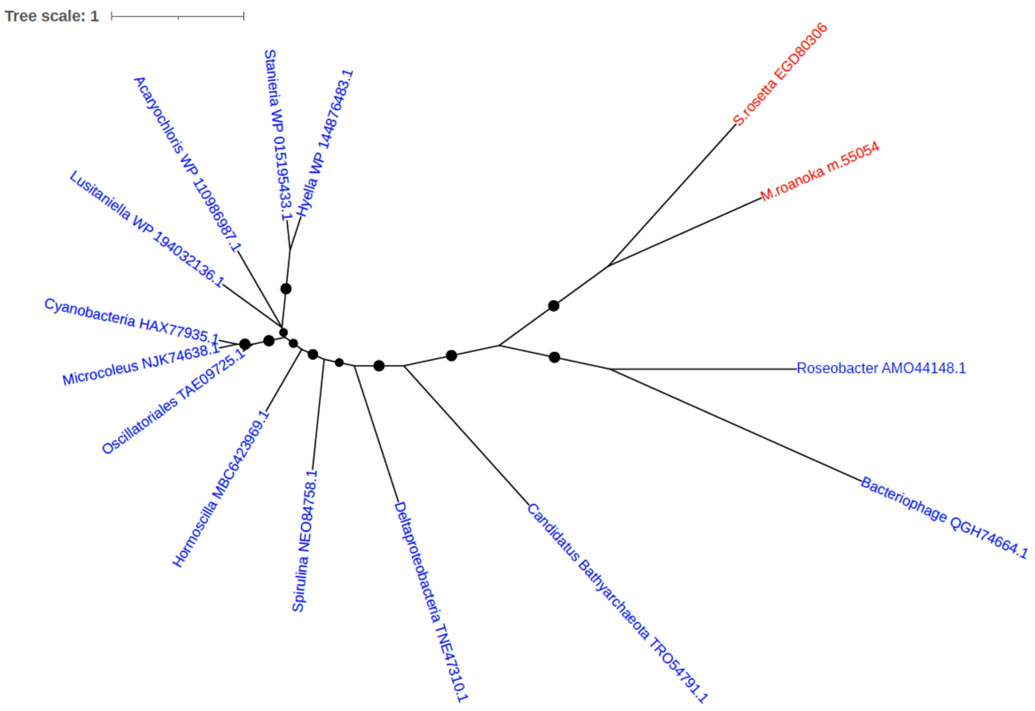

Figure S1-108. Uncharacterized protein (EGD80306)

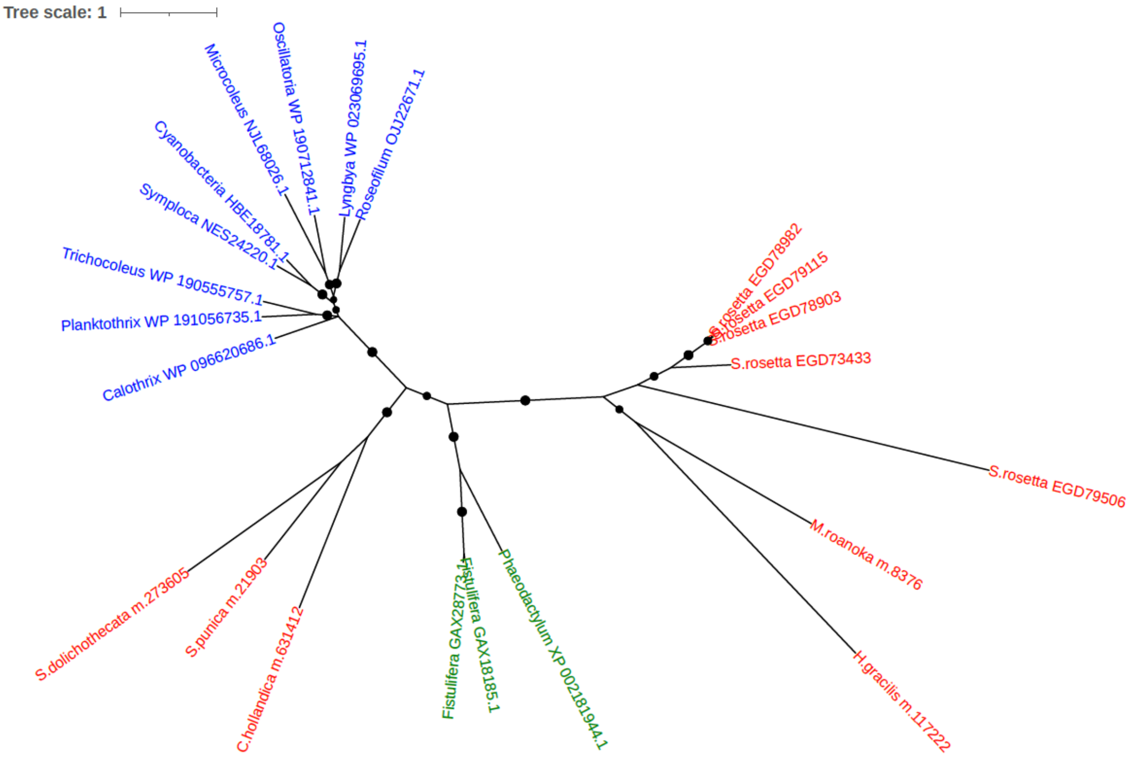

Figure S1-109. Uncharacterized protein (EGD78982, EGD79115, EGD78903, EGD73433, EGD79506)

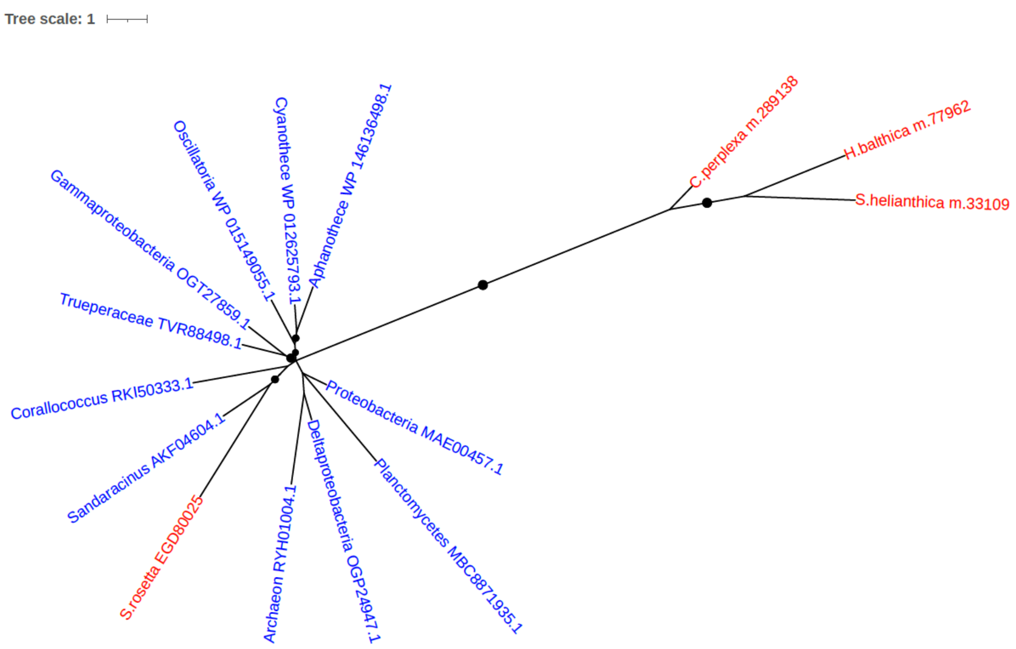

Figure S1-110. Uncharacterized protein (EGD80025)

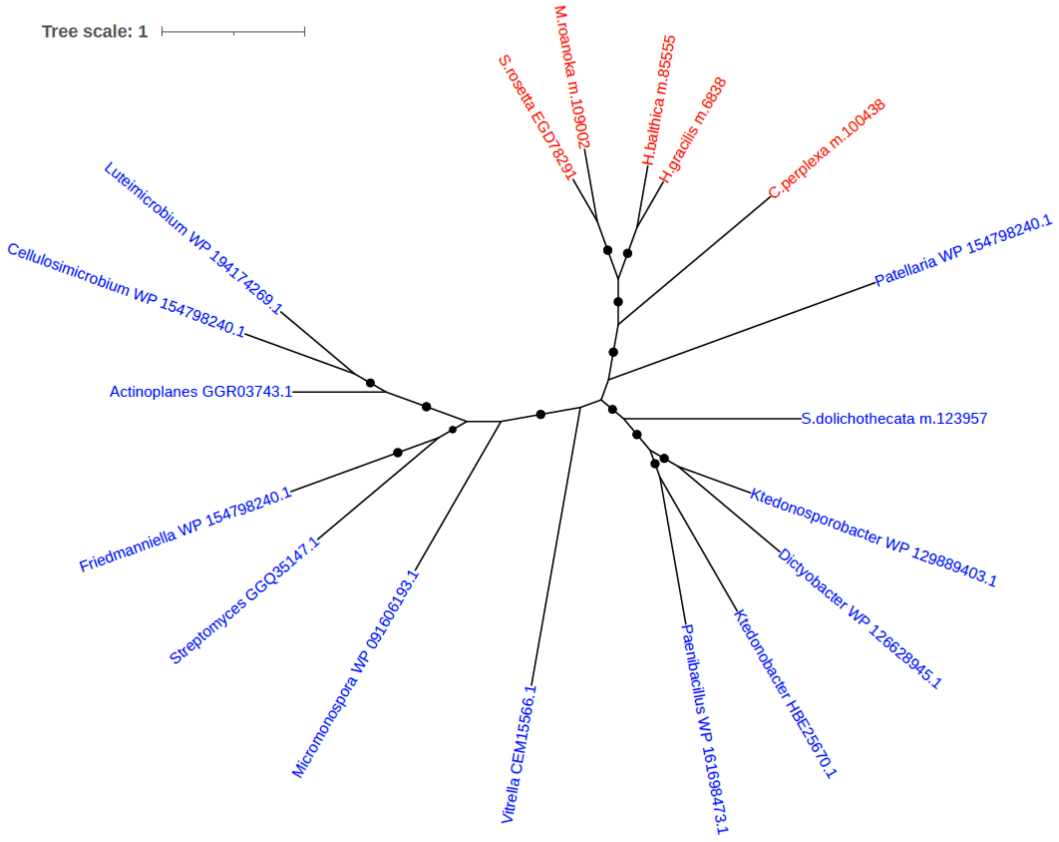

Figure S1-111. Uncharacterized protein (EGD78291)

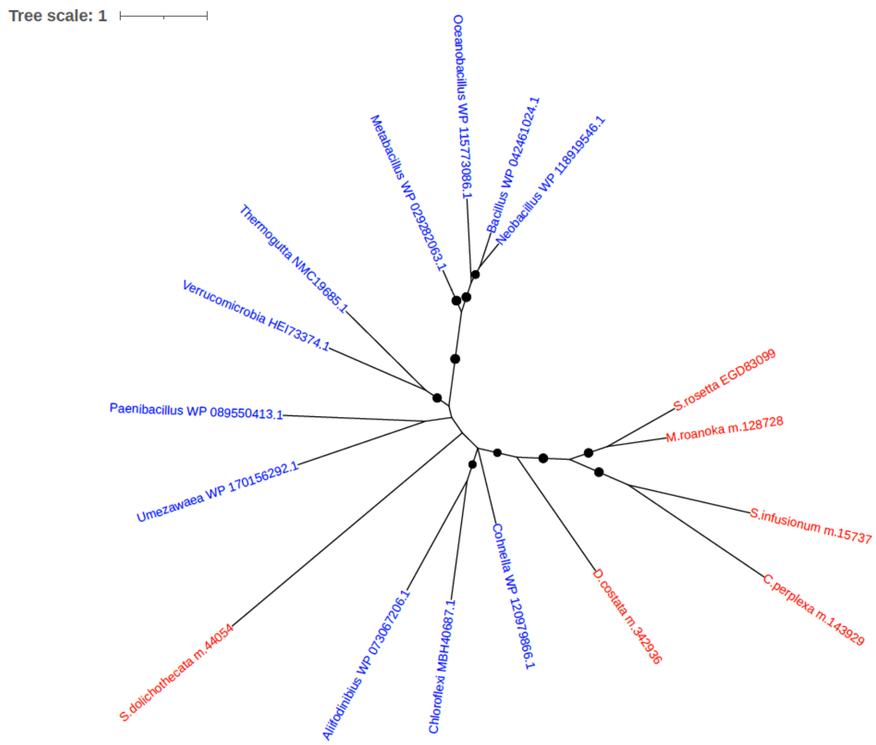

Figure S1-112. Uncharacterized protein (EGD78291)

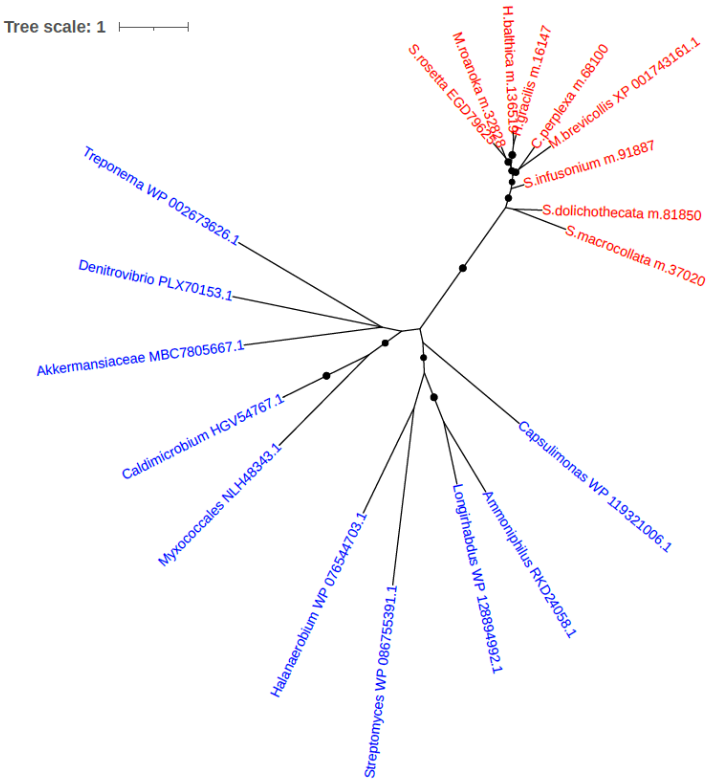

Figure S1-113. Uncharacterized protein (EGD79625)

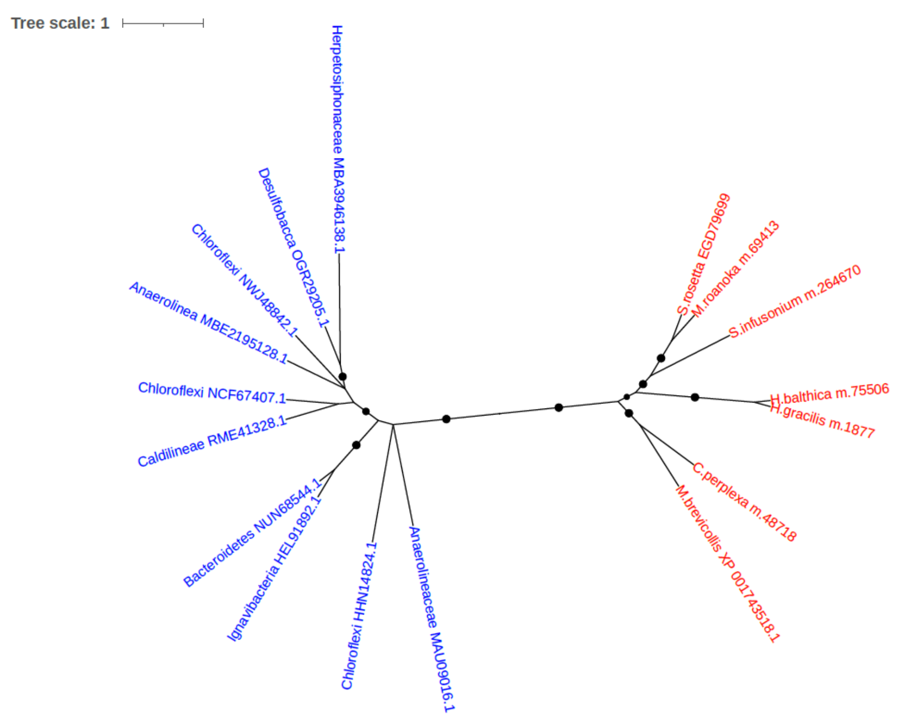

Figure S1-114. Uncharacterized protein (EGD79699)

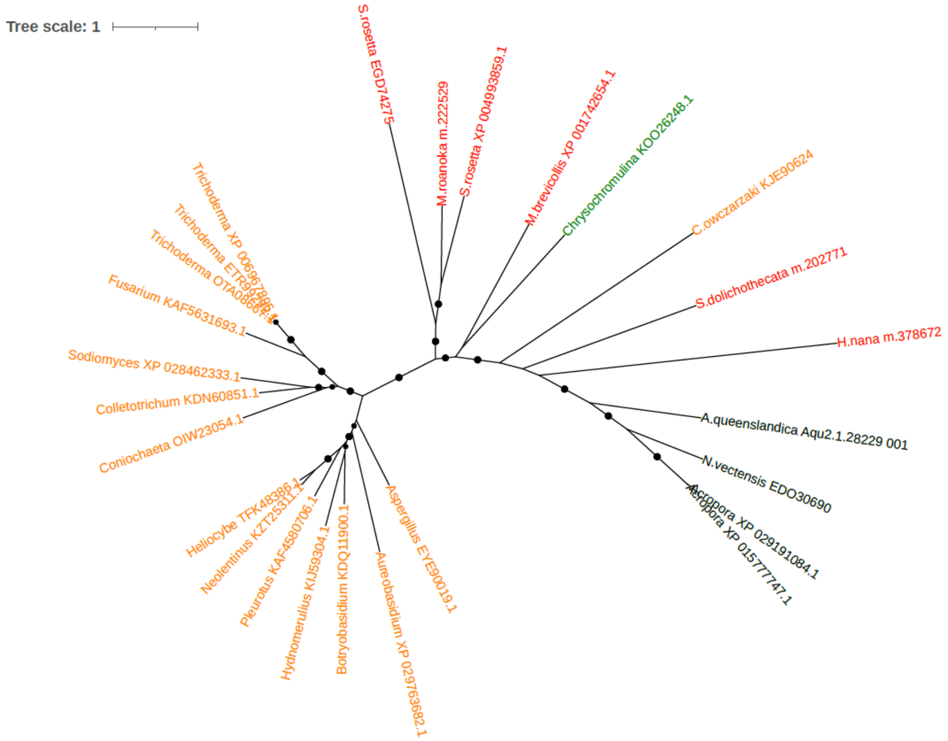

Figure S1-115. Uncharacterized protein (EGD74275)

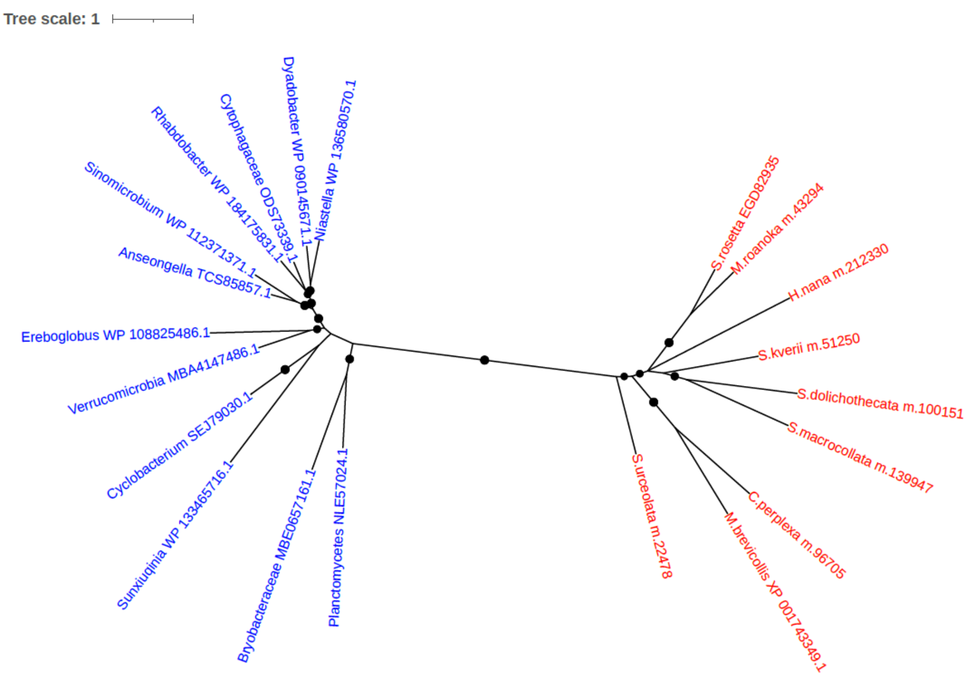

Figure S1-116. Uncharacterized protein (EGD82935)

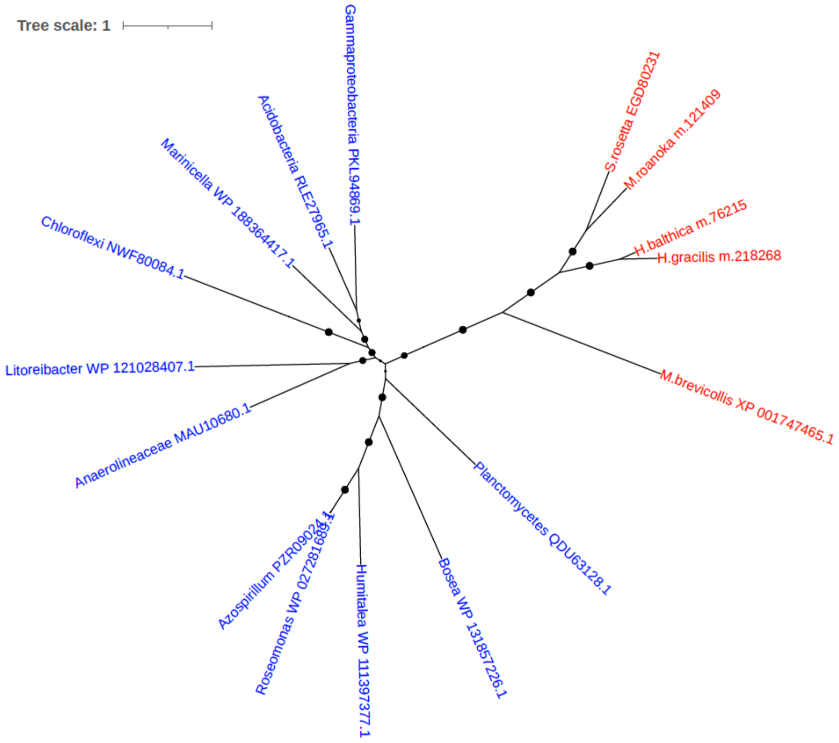

Figure S1-117. Uncharacterized protein (EGD80231)

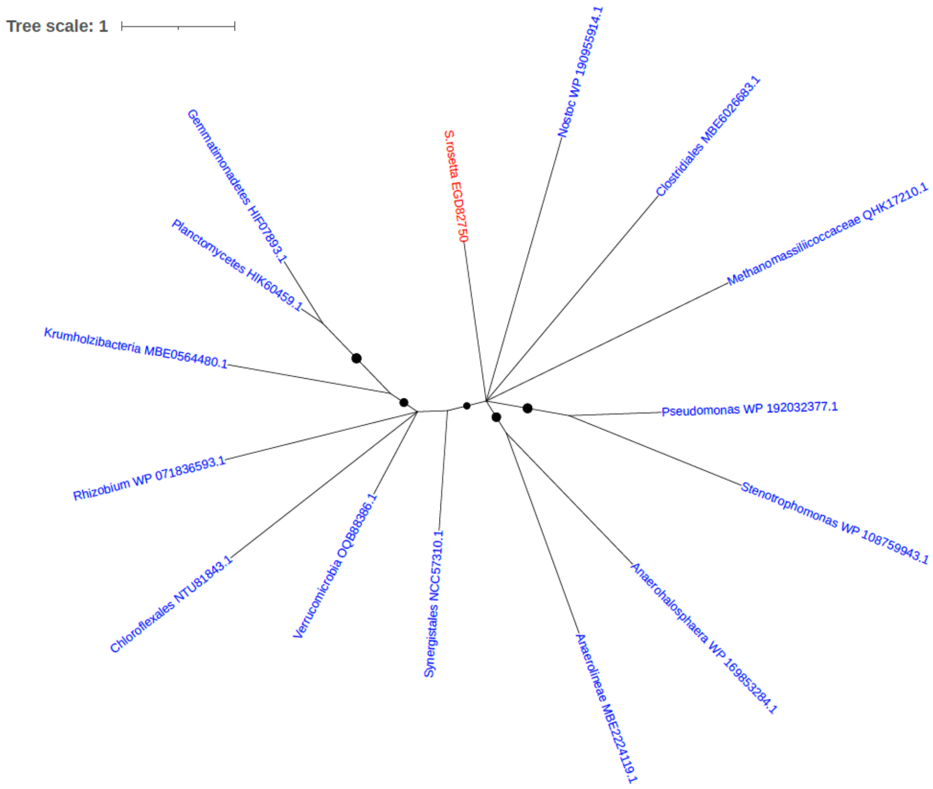

Figure S1-118. Uncharacterized protein (EGD82750)

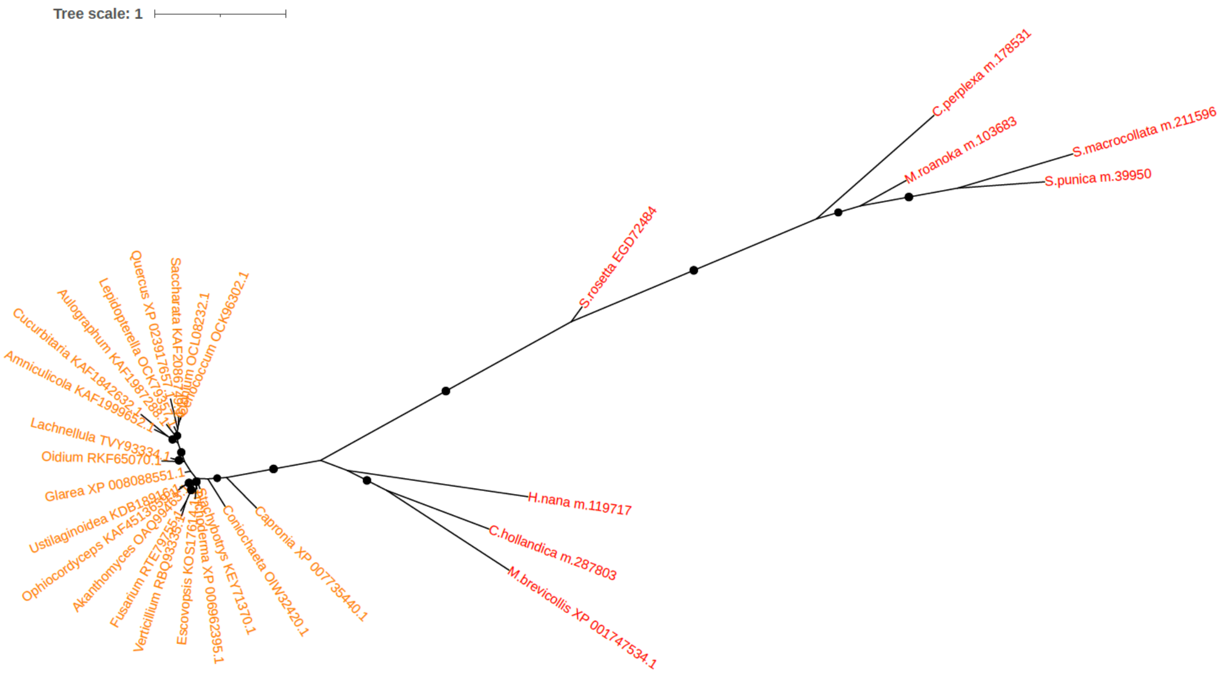

Figure S1-119. Uncharacterized protein (EGD72484)

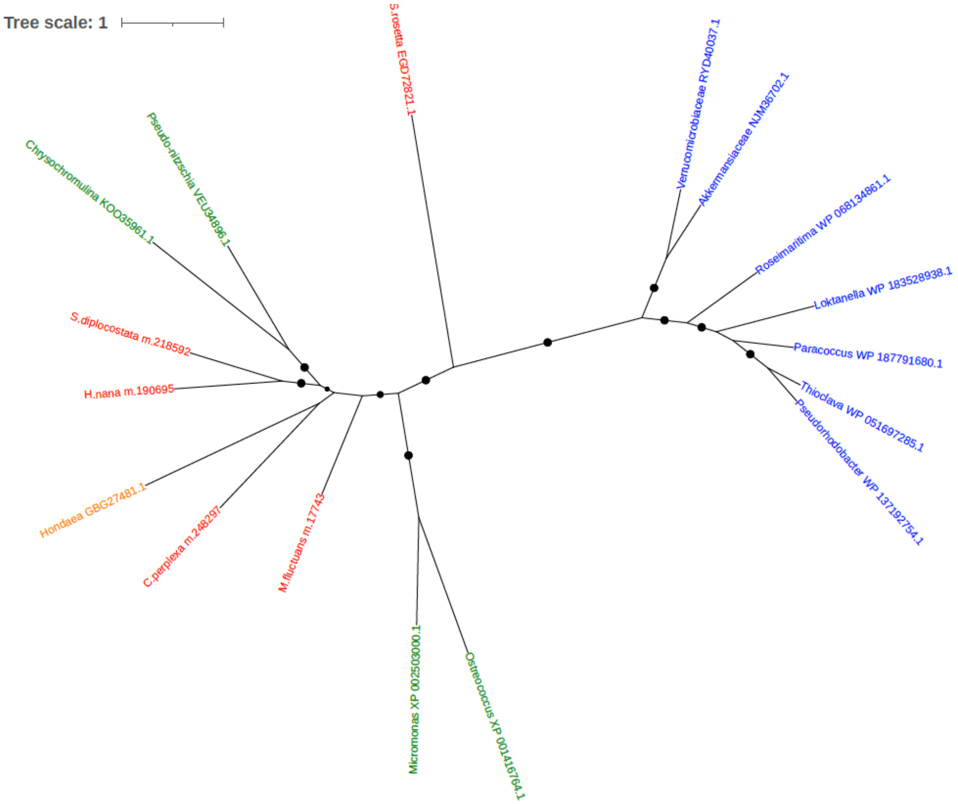

Figure S1-120. Uncharacterized protein (EGD72821)

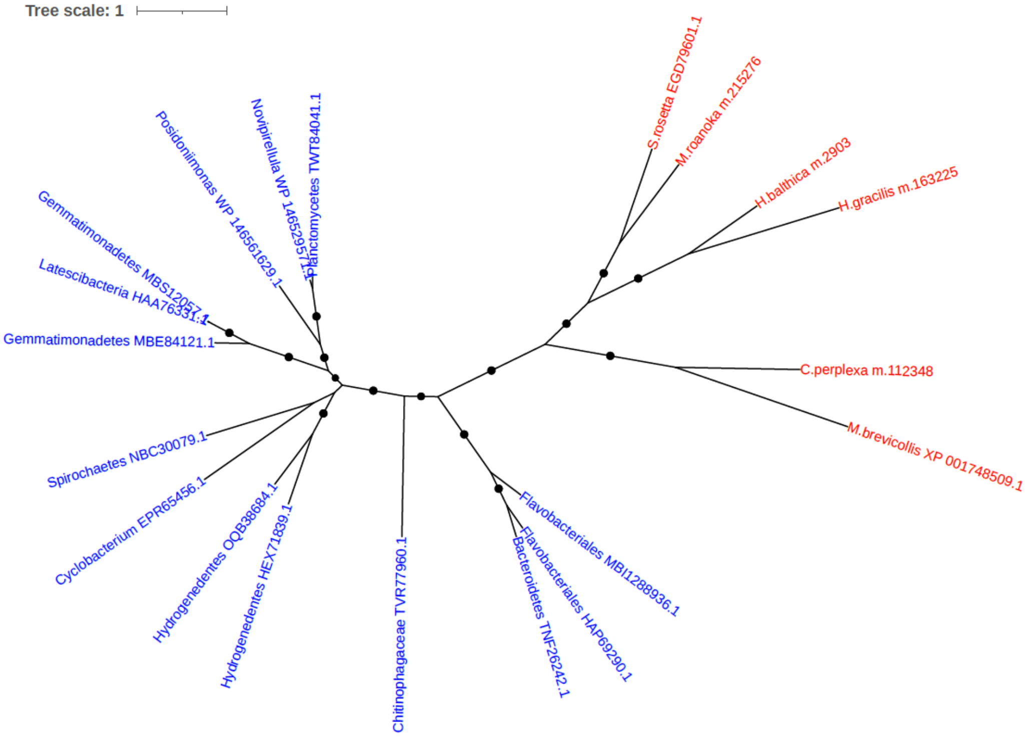

Figure S1-121. Uncharacterized protein (EGD79601)

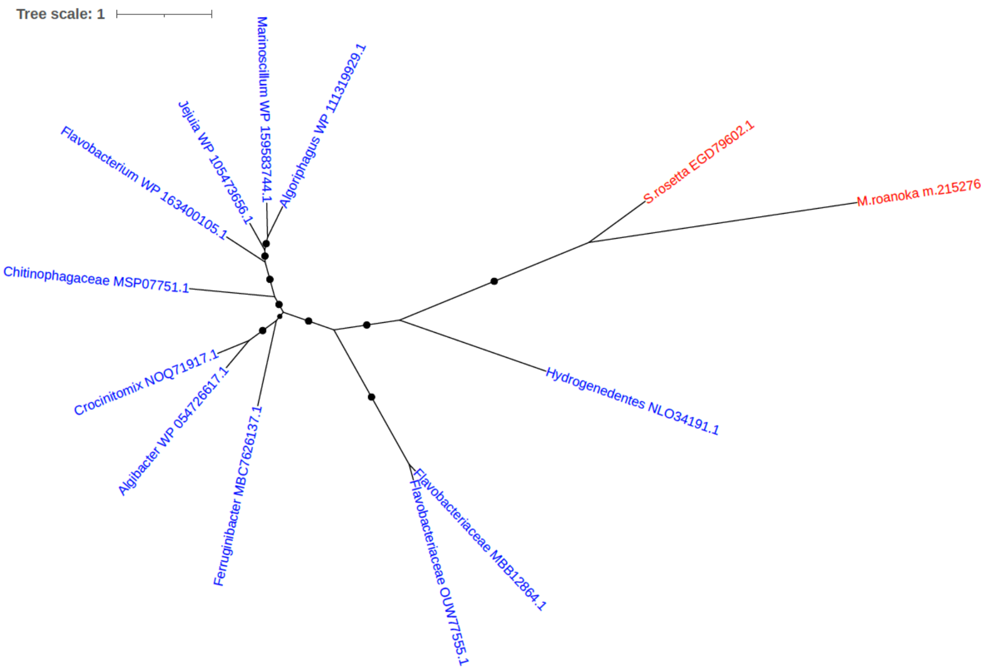

Figure S1-122. Uncharacterized protein (EGD79602)

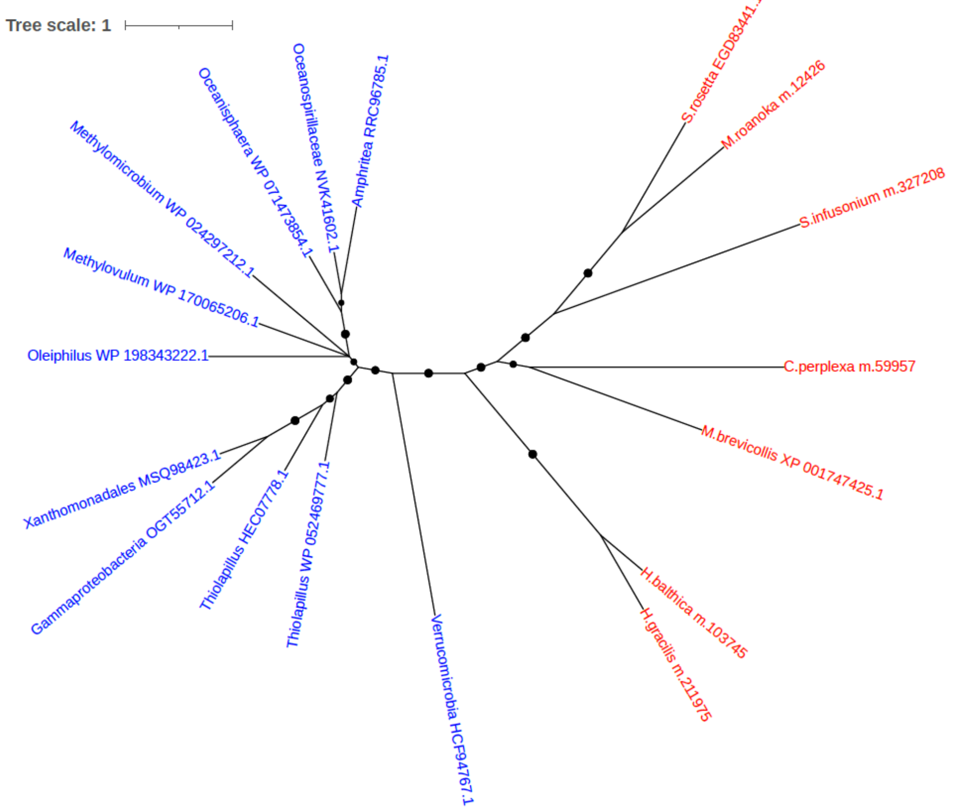

Figure S1-123. Uncharacterized protein (EGD83441)

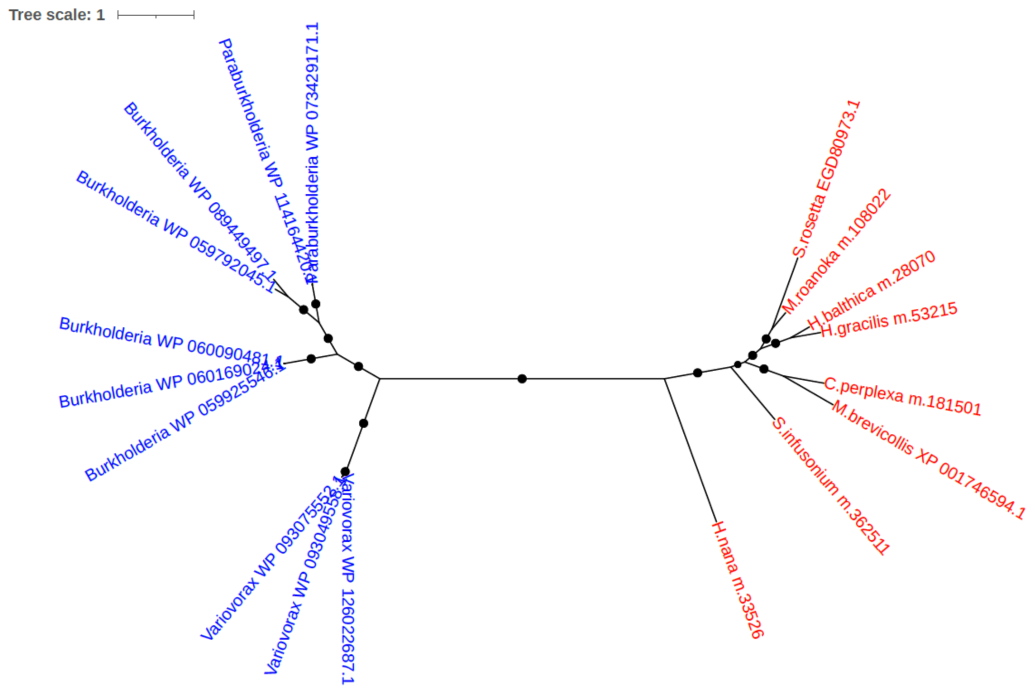

Figure S1-124. Uncharacterized protein (EGD80973)

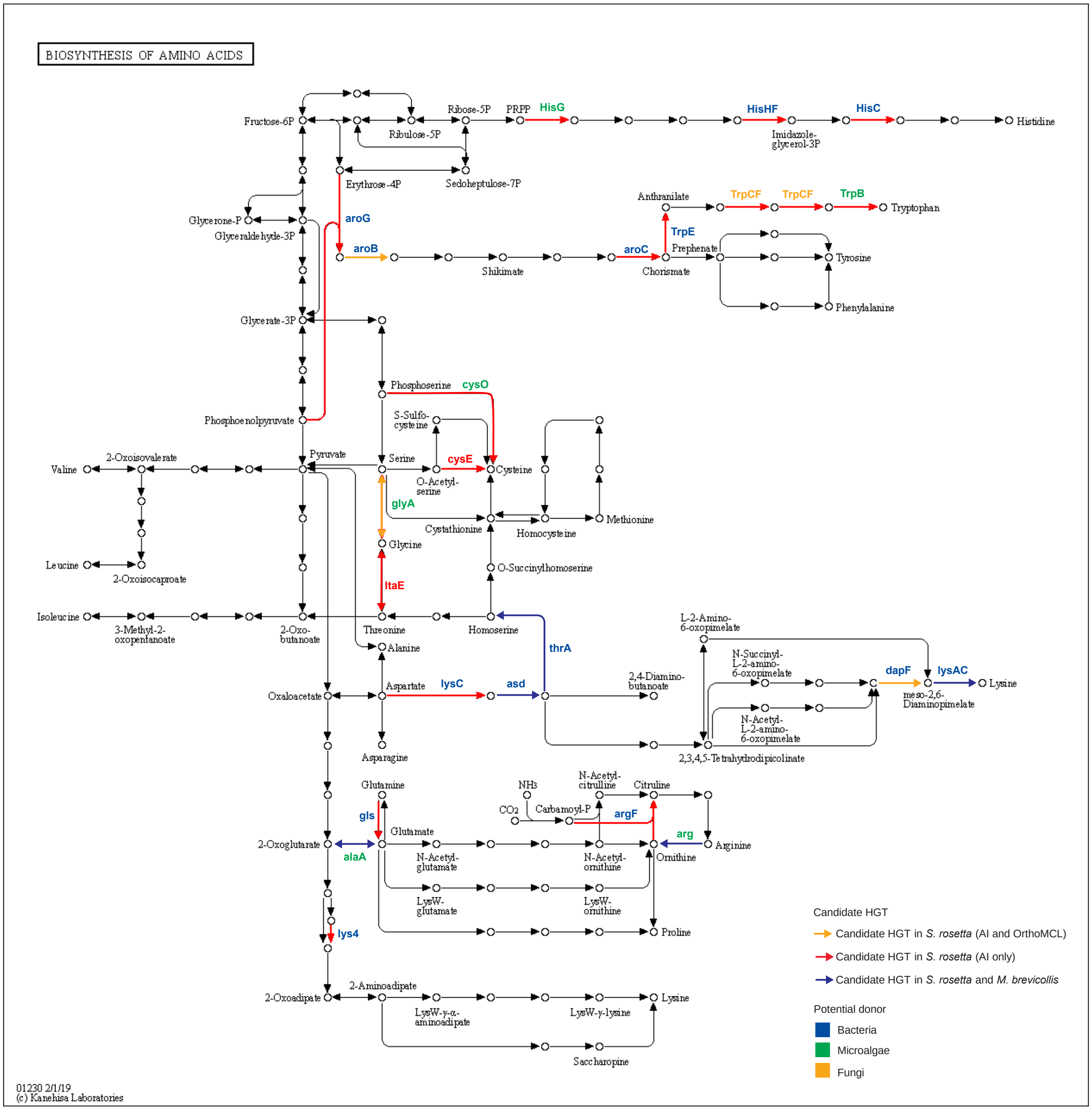

Figure S2. Candidate HGTs in amino acid biosynthetic pathways. Pathway map showing genes identified as HGTs in both *Salpingoeca rosetta* and *Monosiga brevicollis* (blue lines). Candidate HGTs in *S. rosetta* that passed the Alien Index and OrthoMCL filters are indicated by yellow lines, while those that passed only the Alien Index filter are indicated by red lines. Text colors indicate potential donors of candidate HGTs (blue, bacteria; green, microalgae; yellow, fungi). The pathway map image was obtained from the Kyoto Encyclopedia of Genes and Genomes[4] (https://www.kegg.jp/kegg/) and edited using Adobe Illustrator version 24.2.1.

References

1. Richter, D. J., Fozouni, P., Eisen, M. B. & King, N. Gene family innovation, conservation and loss on the animal stem lineage. *Elife* **7**, (2018).
2. Ronquist, F. *et al.* Mrbayes 3.2: Efficient bayesian phylogenetic inference and model choice across a large model space. *Syst. Biol.* **61**, 539–542 (2012).
3. Letunic, I. & Bork, P. Interactive Tree of Life (iTOL) v4: Recent updates and new developments. *Nucleic Acids Res.* **47**, (2019).
4. Kanehisa, M. & Goto, S. KEGG: Kyoto Encyclopedia of Genes and Genomes. *Nucleic Acids Research* (2000). doi:10.1093/nar/28.1.27
